# Supplementary material for: The impact of self-isolation on psychological wellbeing in adults and how to reduce it: A systematic review
Source: PLoS One. 2025 Mar 28;20(3):e0310851. doi: 10.1371/journal.pone.0310851 (PMC11952258; doi:10.1371/journal.pone.0310851)
Supplement: S2 Appendix — (PDF) [file pone.0310851.s002.pdf]

Supplementary materials 2

The impact of self-isolation on psychological wellbeing and how to reduce it: a systematic review

Alex F. Martin[1, 2*], Louise E. Smith[1, 2], Samantha K. Brooks[1, 2], Madeline V. Stein[1], Rachel Davies[1], Richard Amlôt[2, 3], Neil Greenberg[1, 2], G James Rubin[1,2]

[1] King's College London, Institute of Psychiatry, Psychology and Neuroscience, London, UK

[2] NIHR Health Protection Research Unit in Emergency Preparedness and Response, London, UK

[3] UK Health Security Agency, Chief Scientific Officer's Group, UK

Contents

S2 APPENDIX: STUDY PROTOCOL AND DEVIATIONS FROM THE PROTOCOL PUBLISHED ON PROSPERO 2

S2.1 Table. Study protocol and deviations from the protocol published on PROSPERO 2

S2 Appendix: Study protocol and deviations from the protocol published on PROSPERO

S2.1 Table. Study protocol and deviations from the protocol published on PROSPERO

Additional exclusion criteria and clarification of the application of existing criteria	The initial screening against the criteria specified in the protocol produced 164 citations for inclusion, deemed too many for effective synthesis. To reduce the number of studies, we examined heterogeneity in the study populations. We excluded studies that examined populations of children and students (N=23), health care workers (N=23), managed isolation (for example, hotels and self-isolation due to travel because usually this was in a hotel or other institution) N=82. Note that excluding isolation in a hospital setting was already specified in the pre-registration.
	We also excluded studies, or specific findings from studies, where the wellbeing outcome was not specified in the search terms, which could lead to non-identification of all studies related to this outcome. Two quantitative studies were excluded because the outcome was intimate partner violence.
	During the pilot training set, many studies were identified where the definition of isolation and quarantine did not match our definition for the purpose of this review. For example, isolation could refer to self-imposed isolation due to vulnerability, or lockdown measures. Therefore, additional guidance for reviewers was added:<br>- For full-text screening, the abstract must, in principle, have captured self-isolation as defined for this study.<br>- Once full text-screening was complete, if it remained unclear whether self-isolation matched our definition, we contacted the authors. If it remained unclear, we excluded the study from the review.
	We also introduced additional criteria to aid screening/searching:<br>- For any of the excluded characteristics, we allowed up to 5% of participants to have that characteristic before excluding the study.<br>- Grey literature was only included if it investigated the effectiveness of an intervention to ensure only the most rigorous non-peer-reviewed studies were included and

	because of the dearth of peer-reviewed data on this specific topic.
Other deviations	We included a search of the Embase database, which was not specified in the protocol. The protocol specified the use of NIH quality assessment tools. Following consultation of the Cochrane handbook, we decided instead to use the Risk Of Bias In Non-randomized Studies (ROBINS) for exposure and intervention studies. We therefore did not collect data on funding information as planned, as this is not required for the ROBINS assessment. We used the CASP assessment tool for the qualitative studies because the Cochrane recommended ROBINS tools do not currently include a qualitative assessment.
