# Supplementary material for: The impact of self-isolation on psychological wellbeing in adults and how to reduce it: A systematic review
Source: PLoS One. 2025 Mar 28;20(3):e0310851. doi: 10.1371/journal.pone.0310851 (PMC11952258; doi:10.1371/journal.pone.0310851)
Supplement: S3 Appendix — (PDF) [file pone.0310851.s003.pdf]

Supplementary materials 3

The impact of self-isolation on psychological wellbeing and how to reduce it: a systematic review

Alex F. Martin[1, 2*], Louise E. Smith[1, 2], Samantha K. Brooks[1, 2], Madeline V. Stein[1], Rachel Davies[1], Richard Amlôt[2, 3], Neil Greenberg[1, 2], G James Rubin[1,2]

[1] King's College London, Institute of Psychiatry, Psychology and Neuroscience, London, UK

[2] NIHR Health Protection Research Unit in Emergency Preparedness and Response, London, UK

[3] UK Health Security Agency, Chief Scientific Officer's Group, UK

Contents

S3 APPENDIX: SEARCH STRATEGY 2

S3.1 Table. Database search example 2

S3.2 Table. Website search (wellbeing only, reported on the right-hand side of the PRISMA flow diagram in the main text) 3

S3.3 Table. Organisation search (wellbeing only, reported on the right-hand side of the PRISMA flow diagram in the main text) 4

S3.4 Table. Grey literature databases search (wellbeing only, reported on the right-hand side of the PRISMA flow diagram in the main text). 5

S3.5 Table. Google search (wellbeing only, reported on the right-hand side of the PRISMA flow diagram in the main text) 6

S3 Appendix: Search strategy

S3.1 Table. Database search example

#	Query
1	(coronavirus or covid* or sars-cov-2 or ncov2019).mp. or exp Coronavirus/ or exp COVID-19/ or exp SARS-CoV-2/
2	(isolat* or quarantin* or confinement).mp. or exp Patient Isolation/ or exp Quarantine/
3	2 not "social isolation".mp. [mp=title, book title, abstract, original title, name of substance word, subject heading word, floating sub-heading word, keyword heading word, organism supplementary concept word, protocol supplementary concept word, rare disease supplementary concept word, unique identifier, synonyms]
4	(adheren* or compliance or wellbeing or well-being or "quality of life" or resilien* or coping or flourish* or "positive psychology" or "posttraumatic growth" or "post-traumatic growth" or "life satisfaction" or "personal satisfaction" or "psychosocial functioning" or "mental health" or anxiety or depress* or ptsd or trauma* or psychiatric or "psychological stress" or "social stigma" or distress* or mood* or emotion* or "substance abuse" or "substance misuse" or "substance use" or "hazardous drinking" or "alcohol use" or "alcohol abuse" or "alcohol misuse" or alcoholi* or sleep or insomnia or loneliness).mp. or exp Guideline Adherence/ or exp "Treatment Adherence and Compliance"/ or exp Compliance/ or exp Patient Compliance/ or exp "Quality of Life"/ or exp Resilience, Psychological/ or exp Psychology, Positive/ or exp Posttraumatic Growth, Psychological/ or exp Personal Satisfaction/ or exp Psychosocial Functioning/ or exp Mental Health/ or exp Anxiety Disorders/ or exp Anxiety/ or exp Panic/ or exp Panic Disorder/ or exp Depression/ or exp Stress Disorders, Post-Traumatic/ or exp Psychological Trauma/ or exp Stress, Psychological/ or exp Social Stigma/ or Psychological Distress/ or exp Emotions/ or exp Sleep/ or exp "Sleep Initiation and Maintenance Disorders"/ or exp Substance Abuse, Intravenous/ or exp Substance-Related Disorders/ or exp Alcoholism/ or exp Alcohol Drinking/ or exp Loneliness/
5	1 and 3 and 4
6	limit 5 to (yr="2020 -Current")

S3.2 Table. Website search (wellbeing only, reported on the right-hand side of the PRISMA flow diagram in the main text)

Number of websites	Website name	Website link(s)	Reports sought for retrieval	Reports not retrieved	Reports assessed for eligibility	Reports included	Reports excluded
**1**	Rijksinstituut voor Volksgezondheid en Milieu (Dutch National Institute for Public Health and the Environment)	https://www.rivm.nl/en/coronavirus-covid-19/research/behaviour	21	0	21	0	21
**2**	iCARE (international COVID-19 Awareness and Responses Evaluation) Study	https://icare.mbmc-cmcm.ca/results-findings/results-1/	1	0	1	0	1
**3**		https://www.mbmc-cmcm.ca/2021/covid19/results-findings/infographics/	1	0	1	0	1
**4**		https://www.mbmc-cmcm.ca/2021/covid19/results-findings/publications/	0	0	0	0	0
**5**	CHARIS (Covid Health and Adherence Research In Scotland) Study	https://www.abdn.ac.uk/iahs/research/health-psychology/publications-documents-2174.php	26	0	26	0	26
		**TOTAL:**	**49**	**0**	**49**	**0**	**49**

S3.3 Table. Organisation search (wellbeing only, reported on the right-hand side of the PRISMA flow diagram in the main text)

	Organisation	Website link(s)	Reports sought for retrieval	Reports not retrieved	Reports assessed for eligibility	Reports included	Reports excluded
**1**	Public Health Wales	https://phw.nhs.wales/	7	0	7	0	7
**2**	HSC Public Health Agency (Northern Ireland)	https://www.publichealth.hscni.net/	0	0	0	0	0
**3**	NI Direct [GOV]	https://www.nidirect.gov.uk/	0	0	0	0	0
**4**	Office for National Statistics	https://www.ons.gov.uk/	0	0	0	0	0
**5**	Welsh Government	https://www.gov.wales/	4	0	4	0	4
**6**	StatsWales	https://statswales.gov.wales/Catalogue	0	0	0	0	0
**7**	Scottish Government	https://www.gov.scot/	16	0	16	0	16
**8**	Northern Ireland Executive	https://www.northernireland.gov.uk/publications	0	0	0	0	0
**9**	Department of Health, Northern Ireland	https://www.health-ni.gov.uk/covid-19-statistics	0	0	0	0	0
**10**	Northern Ireland Statistics and Research Agency	https://www.nisra.gov.uk/statistics/ni-summary-statistics/coronavirus-covid-19-statistics	1	0	1	0	1
**11**	Government of Ireland	https://www.gov.ie/en/	0	0	0	0	0
**12**	Central Statistics Office Ireland	https://www.cso.ie/en/index.html	1	0	1	0	1
**13**	Public Health Scotland	https://publichealthscotland.scot/	16	0	16	0	16
**14**	UK COVID-19: testing initiative evaluation programme	https://www.gov.uk/government/collections/covid-19-testing-initiative-evaluation-programme	7	0	7	0	7
		**TOTAL:**	**52**	**0**	**52**	**0**	**52**

S3.4 Table. Grey literature databases search (wellbeing only, reported on the right-hand side of the PRISMA flow diagram in the main text).

Number of database s	Database name	Website link	Reports sought for retrieval	Reports not retrieved	Reports assessed for eligibility	Reports included	Reports excluded
**1**	Opengrey.eu	https://opengrey.eu	0	0	0	0	0
**2**	WHO	https://www.who.int/en/	0	0	0	0	0
**3**	NTIS (US Department of Commerce)	https://www.ntis.gov	0	0	0	0	0
**4**	WorldCat	https://www.worldcat.org	8	0	8	0	8
**5**	Agency for Healthcare Research and Quality	https://www.ahrq.gov	0	0	0	0	0
		**TOTAL:**	**8**	**0**	**8**	**0**	**8**

Studies already identified in other searches are not included here, so as not to double count them.

S3.5 Table. Google search (wellbeing only, reported on the right-hand side of the PRISMA flow diagram in the main text)

N of websites	Author	Website link	Reports sought for retrieval	Reports not retrieved	Reports assessed for eligibility	Reports included	Reports excluded
**1**	Bahji et al. (2021)	https://www.ncbi.nlm.nih.gov/pmc/articles/PMC8652932/	*	*	*	*	*
**2**	WHO (2020)	Microsoft Word - Mental health considerations 2020-02-14e_en_19MARCH2020_marissa.docx (who.int)	1	0	1	0	1
**3**	Newbigging (2020)	What is the impact of self-isolation and quarantining on our mental health? - University of Birmingham	1	0	1	0	1
**4**	SPI-B (2020)	S0759_SPI-B__The_impact_of_financial_and_other_targeted_support_on_rates_of_self-isolation_or_quarantine_.pdf (publishing.service.gov.uk)	1	0	1	0	1
**5**	Parker et al. (2022)	JMIR Formative Research - A Brief, Daily, Online Mental Health and Well-being Intervention for University Staff During the COVID-19 Pandemic: Program Description and Outcomes Using a Mixed Methods Design	1	0	1	0	1
**6**	Brog et al. (2021)	An internet-based self-help intervention for people with psychological distress due to COVID-19: study protocol for a randomized controlled trial | Trials | Full Text (biomedcentral.com)	1	0	1	0	1
**7**	Pietrabissa & Simpson (2020)	Frontiers | Psychological Consequences of Social Isolation During COVID-19 Outbreak (frontiersin.org)	1	0	1	0	1
**8**	Dagnino et al. (2020)	Frontiers | Psychological Effects of Social Isolation Due to Quarantine in Chile: An Exploratory Study (frontiersin.org)	1	0	1	0	1
**9**	Wang et al. (2021)	https://www.nature.com/articles/s41380-021-01019-y	1	0	1	0	1
**10**	Young Minds (2020)	Looking After Your Mental Health While Self-isolating | YoungMinds	1	0	1	0	1
**11**	NHS (nd)	Mental wellbeing while staying at home - Every Mind Matters - NHS (www.nhs.uk)	1	0	1	0	1
**12**	Williams et al. (2021)	Interventions to reduce social isolation and loneliness during COVID-19 physical distancing measures: A rapid systematic review | PLOS ONE	1	0	1	0	1

N of websites	Author	Website link	Reports sought for retrieval	Reports not retrieved	Reports assessed for eligibility	Reports included	Reports excluded
**13**	Henssler et al. (2021)	Mental health effects of infection containment strategies: quarantine and isolation—a systematic review and meta-analysis | SpringerLink	*	*	*	*	*
**14**	Soklaridis et al. (2020)	Mental health interventions and supports during COVID- 19 and other medical pandemics: A rapid systematic review of the evidence - ScienceDirect	1	0	1	0	1
**15**	Schwartz et al. (2021)	COVID-19 and Student Well-Being: Stress and Mental Health during Return-to-School - Kelly Dean Schwartz, Deinera Exner-Cortens, Carly A. McMorris, Erica Makarenko, Paul Arnold, Marisa Van Bavel, Sarah Williams, Rachel Canfield, 2021 (sagepub.com)	1	0	1	0	1
**16**	Hossain et al. (2020)	Mental health outcomes of quarantine and isolation for infection prevention: a systematic umbrella review of the global evidence (e-epih.org)	*	*	*	*	*
**17**	UK Government (2022)	COVID-19 Response: Living with COVID-19 - GOV.UK (www.gov.uk)	1	0	1	0	1
**18**	Bunn (2021)	Mental health and well-being in the context of COVID-19 - POST (parliament.uk)	1	0	1	0	1
**19**	CDC (nd)	SARS | Guidance | Interventions for Community Containment | CDC	1	0	1	0	1
**20**	Scottish Government (2021)	Key findings - Compliance with self-isolation and quarantine measures: literature review - gov.scot (www.gov.scot)	1	0	1	0	1
**21**	Bauerle et al. (2020)	e-mental health intervention to support burdened people in times of the COVID-19 pandemic: CoPE It | Journal of Public Health | Oxford Academic (oup.com)	1	0	1	0	1
**22**	Suman et al. (2022)	The acceptability of a self-guided psychological intervention for patients with COVID-19 in isolation and quarantine | International Journal Of Community Medicine And Public Health (ijcmph.com)	1	0	1	0	1
**23**	CAMH (nd)	Quarantine & isolation | CAMH	1	0	1	0	1
**24**	Cherry (2020)	Protect Your Mental Health During Quarantine (verywellmind.com)	1	0	1	0	1
**25**	Jurblum et al. (2020)	RACGP - Psychological consequences of social isolation and quarantine	1	0	1	0	1

N of websites	Author	Website link	Reports sought for retrieval	Reports not retrieved	Reports assessed for eligibility	Reports included	Reports excluded
**26**	Bahji et al. (2021)	(18) (PDF) Strategies to aid self-isolation and quarantine for individuals with severe and persistent mental illness during the COVID-19 pandemic: A systematic review (researchgate.net)	*	*	*	*	*
**27**	Ankit et al. (2020)	Impact on mental health by "Living in Isolation and Quaranti... : Journal of Family Medicine and Primary Care (lww.com)	1	0	1	0	1
**28**	BMA (nd)	bma-the-impact-of-covid-19-on-mental-health-in-england.pdf	1	0	1	0	1
**29**	Soklaridis et al. (2020)	Mental health interventions and supports during COVID- 19 and other medical pandemics_ A rapid systematic review of the evidence (careknowledge.com)	1	0	1	0	1
**30**	Reagu et al. (2020)	Psychological impact of the COVID-19 pandemic within institutional quarantine and isolation centres and its sociodemographic correlates in Qatar: a cross-sectional study | BMJ Open	*	*	*	*	*
**31**	Positive Mind Works (nd)	Self-Isolation Program | Heading into Quarantine? Let us help you through (positivemindworks.co)	1	0	1	0	1
**32**	Public Health Agency Belfast (2021)	How to self-isolate in a shared house if you or someone you live with has coronavirus | HSC Public Health Agency (hscni.net)	1	0	1	0	1
**33**	European Centre for Disease Prevention and Control (nd)	Guidance on quarantine of close contacts to COVID-19 cases and isolation of COVID-19 cases, 7 January 2022 (europa.eu)	1	0	1	0	1
**34**	National Elf Service (2020)	Quarantine: infection prevention, but at what cost for mental health? - National Elf Service	1	0	1	0	1
**35**	Eraso et al. (2021)	IJERPH | Free Full-Text | Self-Isolation and Quarantine during the UK's First Wave of COVID-19. A Mixed-Methods Study of Non-Adherence (mdpi.com)	*	*	*	*	*
**36**	Lingnan University (2022)	LU designs self-assessment test to improve the psychological wellbeing of people in quarantine - Press Releases - Media - Lingnan University (ln.edu.hk)	1	0	1	0	1
**37**	Imperial College (2020)	Imperial-College-COVID19-NPI-modelling-16-03-2020.pdf	1	0	1	0	1
**38**	Center for the Study of Traumatic Stress (nd)	Psychological Effects of Quarantine (cstsonline.org)	1	0	1	0	1

N of websites	Author	Website link	Reports sought for retrieval	Reports not retrieved	Reports assessed for eligibility	Reports included	Reports excluded
**39**	Hossain et al. (2020)	Mental health outcomes of quarantine and isolation for infection prevention: A systematic umbrella review of the global evidence - Abstract - Europe PMC	*	*	*	*	*
**40**	Nuffield Trust (2021)	To solitude: Learning from other countries on how to improve compliance with self-isolation | Nuffield Trust	1	0	1	0	1
**41**	Australian Government (2022)	Pandemic (Quarantine Isolation and Testing Order) 2022 (No. 11).pdf (health.vic.gov.au)	1	0	1	0	1
**42**	Sanders (2020)	COVID-19, social isolation and loneliness | Iriss	1	0	1	0	1
**43**	Kumar et al. (2020)	Full article: COVID 19 and its mental health consequences (tandfonline.com)	1	0	1	0	1
**44**	Australian Government (2022)	nchrac-report-Mental-health-impacts-of-quarantine-and-self-isolation0620.pdf (nhmrc.gov.au)	1	0	1	0	1
**45**	Panchal et al (2023)	The Implications of COVID-19 for Mental Health and Substance Use | KFF	1	0	1	0	1
**46**	Jassim et al. (2021)	Psychological impact of COVID-19, isolation and quarantine | NDT (dovepress.com)	*	*	*	*	*
**47**	NIHR (2021)	Lonely young people have an increased risk of mental health problems years later: research suggests lockdown could have a long term effect (nihr.ac.uk)	1	0	1	0	1
**48**	Smith (2022)	Learning lessons from the UK's self-isolation programme - King's College London (kcl.ac.uk)	1	0	1	0	1
**49**	Ho et al. (2020)	Ho-et-al.-2020-Mental-health-strategies-during-Covid-19.pdf (lancsvrn.co.uk)	1	0	1	0	1
**50**	Organisation for Economic Co-operation and Development (2020)	COVID-19: Protecting people and societies (oecd.org)	1	0	1	0	1
**51**	Nuffield (2020)	Ethical-considerations-in-responding-to-the-COVID-19-pandemic.pdf (nuffieldbioethics.org)	1	0	1	0	1
**52**	Local Government Association (nd)	Public mental health and wellbeing and COVID-19 | Local Government Association	1	0	1	0	1
**53**	Queen's University Belfast (nd)	Supporting Children in Isolation | Coronavirus (COVID-19) | Queen's University Belfast (qub.ac.uk)	1	0	1	0	1

N of websites	Author	Website link	Reports sought for retrieval	Reports not retrieved	Reports assessed for eligibility	Reports included	Reports excluded
**54**	All India Occupational Therapists Federation (nd)	Role of Occupational Therapist in COVID-19 (wfot.org)	1	0	1	0	1
**55**	AMA (nd)	Ethical Use of Quarantine & Isolation | ama-coe (ama-assn.org)	1	0	1	0	1
**56**	Australian government (2020)	National Mental Health and Wellbeing Pandemic Response Plan (mentalhealthcommission.gov.au)	1	0	1	0	1
**57**	Okabe-Miyamoto et al. (2020)	Social Connection and Well-Being during COVID-19 | The World Happiness Report	1	0	1	0	1
**58**	Hester (2020)	The toll of COVID-19 and quarantine on college students' mental health (contemporarypediatrics.com)	1	0	1	0	1
**59**	Save the Children (2021)	Integrated Response Framework for Isolation and Quarantine as Non-Pharmaceutical Interventions Against COVID-19 (ready-initiative.org)	1	0	1	0	1
**60**	Louisiana Department of Health (2022)	LDH_COVID-Contact-Tracing-in-Schools.pdf (la.gov)	1	0	1	0	1
**61**	Unicef (2020)	Briefing note on addressing mental health and psycosocial aspects of COVID-19 outbreak.pdf (unicef.org)	1	0	1	0	1
**62**	Physiopedia (nd)	Mental Health Stress and Resilience in Times of COVID-19 - Physiopedia (physio-pedia.com)	1	0	1	0	1
**63**	Zweig et al. (2021)	Ensuring Rights while Protecting Health: The Importance of Using a Human Rights Approach in Implementing Public Health Responses to COVID-19 – Health and Human Rights Journal (hhrjournal.org)	*	*	*	*	*
**64**	Fuchs et al. (2021)	Assessment of a Hotel-Based COVID-19 Isolation and Quarantine Strategy for Persons Experiencing Homelessness | Infectious Diseases | JAMA Network Open | JAMA Network	*	*	*	*	*
**65**	Lockhart et al. (2020)	CYP-MH-Restoration-Recovery-Planning-Literature-Summary-v.2.0.docx (live.com)	1	0	1	0	1
**66**	California Department of Public Health (2020)	https://www.cdph.ca.gov/Programs/CID/DCDC/Pages/COVID-19/Guidance-on-Isolation-and-Quarantine-for-COVID-19-Contact-Tracing-7-30-2020.aspx	1	0	1	0	1
**67**	Lewis & Clark (2020)	COVID-19 Isolation and Quarantine Plan for the Lewis & Clark Community • Health Promotion and Wellness • Lewis & Clark (lclark.edu)	1	0	1	0	1

N of websites	Author	Website link	Reports sought for retrieval	Reports not retrieved	Reports assessed for eligibility	Reports included	Reports excluded
**68**	Cal Poly (nd)	COVID-19 - Campus Health & Wellbeing - Cal Poly, San Luis Obispo	1	0	1	0	1
**69**	Newman University (2020)	Covid-19-Resilience-Document-May-2022-Final.pdf (newman.ac.uk)	1	0	1	0	1
**70**	Vermont Department of Health (nd)	COVID-19 Symptoms & Treatment | Vermont Department of Health (healthvermont.gov)	1	0	1	0	1
**71**	Arizona Department of Health Services (2022)	ADHS 'Release from Isolation and Quarantine' Guidance (azdhs.gov)	1	0	1	0	1
**72**	FutureLearn (nd)	How to Survive Self-Isolation - Blog - FutureLearn	1	0	1	0	1
**73**	Government of Canada (nd)	COVID-19: Prevention and risks - Canada.ca	1	0	1	0	1
**74**	Vanderbilt University (nd)	Support During Quarantine and Isolation | Student Care Network | Vanderbilt University	1	0	1	0	1
**75**	New Zealand Government (2020)	COVID-19 Preliminary Psychosocial and Mental Wellbeing Recovery Plan (health.govt.nz)	1	0	1	0	1
**76**	Diamond et al. (2020)	Coronavirus disease 2019: achieving good mental health during social isolation. — Department of Psychiatry (ox.ac.uk)	*	*	*	*	*
**77**	USA Government (nd)	What is the difference between isolation and quarantine? | HHS.gov	1	0	1	0	1
**78**	US Department of Education (nd)	Supporting Students During the COVID-19 Pandemic: Maximizing In-Person Learning and Implementing Effective Practices for Students in Quarantine and Isolation | U.S. Department of Education	1	0	1	0	1
**79**	Norwegian Institue of Public Health (nd)	Coronavirus disease - NIPH (fhi.no)	1	0	1	0	1
**80**	New York (2023)	Monroe County, NY - COVID-19 Resources	1	0	1	0	1
**81**	Office for Students (2020)	Supporting student mental health - Office for Students	1	0	1	0	1
**82**	Voyae Well (nd)	Voyage Well | Virgin Voyages	1	1	0	0	0
**83**	Unknown	Revised_Travel_protocol_revised_2nd_April_2022.pdf (ncdc.gov.ng)	1	1	0	0	0

N of websites	Author	Website link	Reports sought for retrieval	Reports not retrieved	Reports assessed for eligibility	Reports included	Reports excluded
**84**	Wikipedia (nd)	COVID-19 pandemic - Wikipedia	1	0	1	0	1
**85**	Tulane University (2020)	Understanding the Effects of Social Isolation on Mental Health (tulane.edu)	1	0	1	0	1
**86**	Australian Government (2023)	CHOICE travel insurance buying guide | Smartraveller	1	0	1	0	1
**87**	Czech Government (2022)	Measures adopted by the Czech Government against the coronavirus | Government of the Czech Republic (vlada.cz)	1	0	1	0	1
**88**	State of New Jersey (2022)	https://www.cityofsummit.org/660/COVID-19	1	0	1	0	1
**89**	Cayman Islands Government (2023)	COVID-19 Frequently Asked Questions (exploregov.ky)	1	0	1	0	1
**90**	WebMD (2023)	Review Supports Continued Mask-wearing in Health Care Visits (webmd.com)	1	0	1	0	1
**91**	Johns Hopkins (2020)	Coronavirus, Social and Physical Distancing and Self-Quarantine | Johns Hopkins Medicine	1	0	1	0	1
**92**	Monson Town Government (2022)	Coronavirus / COVID19 Information | Town of Monson MA (monson-ma.gov)	1	0	1	0	1
**93**	na	Click Allow (solidcaptcha.lm.r.appspot.com)	1	1	1	0	0
**94**	US Department of Health and Human Services (nd)	SAMHSA's National Helpline | SAMHSA	1	0	1	0	1
**95**	Moustafa (nd)	Mental Health Effects of COVID-19 - Google Books	1	0	1	0	1
**96**	Viditch (nd)	Germs at Bay: Politics, Public Health, and American Quarantine - Charles Vidich - Google Books	1	0	1	0	1
**97**	Freeman (nd)	The Ethics of Public Health, Volumes I and II - Google Books	1	0	1	0	1
**98**	Bahji et al. (2021)	Strategies to aid self-isolation and quarantine for individuals with severe and persistent mental illness during the COVID-19 pandemic: A systematic review | Psychiatric Research and Clinical Practice (psychiatryonline.org)	*	*	*	*	*

N of websites	Author	Website link	Reports sought for retrieval	Reports not retrieved	Reports assessed for eligibility	Reports included	Reports excluded
**99**	Hossain et al. (2020)	Mental health outcomes of quarantine and isolation for infection prevention: a systematic umbrella review of the global evidence - PMC (nih.gov)	*	*	*	*	*
**100**	Jain et al. (2020)	Impact on mental health by "Living in Isolation and Quarantine" during COVID-19 pandemic - PMC (nih.gov)	*	*	*	*	*
		**TOTAL:**	**86**	**3**	**83**	**0**	**83**

* Study had already been identified in a previous search (database or other grey literature) and therefore was not screened again
