# Supplementary material for: The impact of self-isolation on psychological wellbeing in adults and how to reduce it: A systematic review
Source: PLoS One. 2025 Mar 28;20(3):e0310851. doi: 10.1371/journal.pone.0310851 (PMC11952258; doi:10.1371/journal.pone.0310851)
Supplement: S4 Appendix — (PDF) [file pone.0310851.s004.pdf]

Supplementary materials 4

The impact of self-isolation on psychological wellbeing and how to reduce it: a systematic review

Alex F. Martin[1, 2*], Louise E. Smith[1, 2], Samantha K. Brooks[1, 2], Madeline V. Stein[1], Rachel Davies[1], Richard Amlôt[2, 3], Neil Greenberg[1, 2], G James Rubin[1,2]

[1] King's College London, Institute of Psychiatry, Psychology and Neuroscience, London, UK

[2] NIHR Health Protection Research Unit in Emergency Preparedness and Response, London, UK

[3] UK Health Security Agency, Chief Scientific Officer's Group, UK

Contents

S4 APPENDIX: EXCLUSION REASONS FOR FULL TEXT SCREENED CITATIONS 2

S4.1 Exclusion reasons for full text screened citations 2

S4 Appendix: Exclusion reasons for full text screened citations

Citation screening was completed for this systematic review and a systematic review investigating adherence to self-isolation. Therefore, these totals include citations screened for both systematic reviews.

S4.1 Exclusion reasons for full text screened citations

Reference	Included in adherence	Included in wellbeing	Not isolation/quarantine as per study definition	Sample includes those who isolated in hospital	Full paper cannot be located	Query re inclusion and no author response / no way to contact authors	No primary data reported or analysed	ADHERENCE: No outcome	ADHERENCE: Attitudes or intentions, not behaviours	ADHERENCE - no quantitative measure	WELLBEING: No outcome	WELLBEING: no comparison group or predictor / intervention	WELLBEING: Sample includes those who isolated NOT at home	WELLBEING: Sample includes children/students	WELLBEING: Sample includes HCWs
Aaltonen, K. I., Saarni, S., Holi, M., & Paananen, M. The effects of mandatory home quarantine on mental health in a community sample during the COVID-19 pandemic. Nordic Journal of Psychiatry. https://doi.org/10.1080/08039488.2022.2061047		x						x							
Abbasi-Kangevari, M., Kolahi, A. A., Ghamari, S. H., & Hassanian-Moghaddam, H. (2021). Public Knowledge, Attitudes, and Practices Related to COVID-19 in Iran: Questionnaire Study. JMIR Public Health and Surveillance, 7(2), e21415.			x												

Abid, R., Ammar, A., Maaloul, R., Souissi, N., & Hammouda, O. (2021). Effect of COVID-19-Related Home Confinement on Sleep Quality, Screen Time and Physical Activity in Tunisian Boys and Girls: A Survey. International Journal of Environmental Research & Public Health [Electronic Resource], 18(6), 16.		x		
Abir, T., et al. (2021). "Psychological Impact of COVID-19 Pandemic in Bangladesh: Analysis of a Cross-Sectional Survey." Health Security 19(5): 468-478.	x		x	
Abolfotouh, M. A., et al. (2021). "Behavior Responses and Attitude of the Public to COVID-19 Pandemic During Movement Restrictions in Saudi Arabia." International Journal of General Medicine 14: 741-753.		x		
Abu Kamel, A. K. and E. K. Alnazly (2022). "The impact of confinement on older Jordanian adults' mental distress during the COVID-19 pandemic: A web-based cross-sectional study." Perspectives in Psychiatric Care 58(1): 17-30.		x		
Abu Shaphe, M., et al. (2021). "Psychological effects of Post-Quarantine Following Outbreak of "COVID-19"." Eurasian Journal of Medicine and Oncology 5(1): 85-87.			x	x
Adams, C., et al. (2021). "Understanding the impact of isolation due to COVID-19 on employment for Kentuckians with spinal cord injuries." Journal of Vocational Rehabilitation 54(1): 43-49.		x		
Adler, A. B., et al. (2022). "US soldiers and the role of leadership: COVID-19, mental health, and adherence to public health guidelines." BMC Public Health 22(1).		x		

Agnihotri, K., & Srivastava, S. K. (2022). Upshot of coronavirus on the teenagers of Indian subcontinent and Middle East. International Journal of Knowledge and Learning, 15(3), 233-252. https://doi.org/10.1504/ijkl.2022.123960	x										
Agrawal, S. and A. Agarwal (2021). "Mental health among patients in a COVID-19-dedicated facility." Transactions of the Royal Society of Tropical Medicine and Hygiene 115(1): 1-2.						x			x		
Aguilar, E. A., et al. (2022). "Assessment of depression and anxiety in residents of a continuing care retirement community during the COVID-19 pandemic quarantine." Journal of Investigative Medicine 70(2): 487-488.	x										
Ahmadi, S., et al. (2022). "Explaining Experiences, Challenges and Adaptation Strategies in COVID-19 Patients: A Qualitative Study in Iran." Frontiers in Public Health 9.		x									
Ahmed, M. A. M., et al. (2020). "Covid-19 in somalia: Adherence to preventive measures and evolution of the disease burden." Pathogens 9(9): 1-11.	x										
Ajmi, S., Hentati, S., Masmoudi, R., Sellami, R., Baati, I., Feki, I., & Masmoudi, J. (2021). [Conference Abstract]. European Psychiatry, 64(Supplement 1), S282-S283.	x										
Akbari, P., et al. (2021). "Housing and mental health during outbreak of COVID-19." Journal of Building Engineering 43.	x										
Akgun, S., et al. (2021). "COMPARISON OF PSYCHOLOGICAL DISTRESS AND MENTAL HEALTH LITERACY LEVELS OF HOSPITALIZED COVID-19 PATIENTS, INDIVIDUALS UNDER QUARANTINE, AND HEALTHY INDIVIDUALS OF SOCIETY IN THE PANDEMIC." Asean Journal of Psychiatry 22(8).						x					x

Akhmadullina, I. A., et al. (2020). "Pandemic Covid-19-As a factor of Quarantine Measures Implementation in Russia." Journal of Research in Medical and Dental Science 8(7): 160-165.	x		
Akinci, M., Pena-Gomez, C., Operto, G., Fuentes-Julian, S., Deulofeu, C., Sanchez-Benavides, G., Mila-Aloma, M., Grau-Rivera, O., Gramunt, N., Navarro, A., Minguillon, C., Fauria, K., Suridjan, I., Kollmorgen, G., Bayfield, A., Blennow, K., Zetterberg, H., Molinuevo, J. L., Suarez-Calvet, M., . . . Study, A. (2022). Prepandemic Alzheimer Disease Biomarkers and Anxious-Depressive Symptoms During the COVID-19 Confinement in Cognitively Unimpaired Adults [Observational Study]. Neurology, 99(14), e1486-e1498.	x		
Al Araimi, F. A. F., Langrial, S. U., & Al Awaidy, S. T. (2021). Public health messages for social behavior change in the wake of COVID-19: A qualitative study. Oman Medical Journal, 36(2) (no pagination), Article e254.	x		
Al Joboory, S., Fernandez, V. A., Remingol, L., Brulin-Solignac, D., & Bouchard, J. P. (2020). [COVID-19, the psychopathological effects of the pandemic]. Soins - Psychiatrie, 41(331), 12-15.	x		
Al Mukhaini, A., Al-Huseini, S., Al Kaabi, S., Chan, M. F., Al Balushi, R., Ganguly, S. S., Al-Adawi, S., & Anwar, H. (2022). Psychological and sleep-related factors among quarantined Omani students returning from abroad. Biological Rhythm Research, 53(8), 1273-1284. https://doi.org/10.1080/09291016.2021.1922826		x	x

Al Zabadi, H., Haj-Yahya, M., Yaseen, N., & Alhroub, T. (2022). Socioeconomic Inequalities in Times of COVID-19 Lockdown: Prevalence and Related-Differences in Measures of Anxiety and Stress in Palestine. Frontiers in Psychology, 13, Article 898845. https://doi.org/10.3389/fpsyg.2022.898845	x
Al Zabadi, H., Yaseen, N., Alhroub, T., & Haj-Yahya, M. (2021). Assessment of Quarantine Understanding and Adherence to Lockdown Measures During the COVID-19 Pandemic in Palestine: Community Experience and Evidence for Action. Frontiers in Public Health, 9, 570242.	x
Al-Awaida, W., et al. (2022). "Psychological effects of quarantine on Syrian refugees, compared to the Jordanian populations." International Migration 60(1): 219-227.	x
Al-Mulla, N. A., & Mahfoud, Z. R. (2022). The Impact of the COVID-19 Lockdown "Home Quarantine" on the Physical Activity and Lifestyle of Children in Qatar [Research Support, Non-U.S. Gov't]. Frontiers in Public Health, 10, 877424.	x
Al-Musharaf, S. (2020). Prevalence and Predictors of Emotional Eating among Healthy Young Saudi Women during the COVID-19 Pandemic. Nutrients, 12(10), 24.	x
Al-Musharaf, S., Aljuraiban, G., Bogis, R., Alnafisah, R., Aldhwayan, M., & Tahrani, A. (2021). Lifestyle changes associated with COVID-19 quarantine among young Saudi women: A prospective study [Research Support, Non-U.S. Gov't]. PLoS ONE [Electronic Resource], 16(4), e0250625.	x

Al-rawi, M. B. A., et al. (2022). "EVALUATION OF KNOWLEDGE ON THE PREVENTIVE PRACTICE OF COVID-19 AMONG MIDDLE-AGED ADULTS IN SAUDI ARABIA-A CROSS-SECTIONAL QUESTIONNAIRE BASED STUDY." Farmacia 70(5): 890-896.			x	x	
Alafif, N. O., Abdelfattah, E. H., Al Hadi, R. A., Alanazi, S. B., Alkabaa, R. I., Alsalem, F. A., Aljeldah, T. M., Aldriweesh, K. K., & Albati, A. A. (2021). Effect of quarantine on eating behaviors and weight change among King Saud University students in Riyadh. Journal of King Saud University Science, 33(8), Article 101609. https://doi.org/10.1016/j.jksus.2021.101609	x				
Alagili, D. E., & Bamashmous, M. (2021). The Health Belief Model as an explanatory framework for COVID-19 prevention practices. Journal of Infection and Public Health, 14(10), 1398-1403.	x				
Alam, M. M. D., Alam, M. Z., Rahman, S. A., & Taghizadeh, S. K. (2021). Factors influencing mHealth adoption and its impact on mental well-being during COVID-19 pandemic: A SEM-ANN approach. Journal of Biomedical Informatics, 116, 103722.		x		x	
Alamri, H. S., et al. (2020). "Prevalence of Depression, Anxiety, and Stress among the General Population in Saudi Arabia during Covid-19 Pandemic." International Journal of Environmental Research and Public Health 17(24).		x			x

Alateeq, D. A., Almughera, H. N., Almughera, T. N., Alfedeah, R. F., Nasser, T. S., & Alaraj, K. A. (2021). The impact of the coronavirus (COVID-19) pandemic on the development of obsessive-compulsive symptoms in Saudi Arabia. Saudi Medical Journal, 42(7), 750-760.	x		
Alateeq, D., Aljabri, A., Aldogiam, G., Alajmi, H., Alsoqaih, H., Alfadhly, R., & Alshahrani, R. (2022). Loneliness and Depression Among Female University Students During the COVID-19 Pandemic: A Cross-Sectional Study in Riyadh, Saudi Arabia, 2020. International Journal of Public Health, 67, 1604885.	x		
Alateeq, D., et al. (2021). "Quarantine-related depression and anxiety during coronavirus disease (COVID-19) outbreak." European Psychiatry 64: S656-S656.		x	x
Alaujan, F. Y. and A. Ghamdi (2020). "Comparison of the Psychological Impact of COVID-19 Pandemic on Saudi Nationals Arriving from Abroad During Institutional Quarantine and a Year Later: An Analytical Cross-Sectional and Follow-Up Study." Journal of Pharmaceutical Research International 33(45A): 455-465.		x	x
Alaujan, F. Y. and A. Ghamdi (2020). "Comparison of the Psychological Impact of COVID-19 Pandemic on Saudi Nationals Arriving from Abroad During Institutional Quarantine and a Year Later: An Analytical Cross-Sectional and Follow-Up Study." Journal of Pharmaceutical Research International 33(45A): 455-465.		x	x

Albano, G., Bonfanti, R. C., Gullo, S., Salerno, L., & Lo Coco, G. (2021). The psychological impact of COVID-19 on people suffering from dysfunctional eating behaviours: A linguistic analysis of the contents shared in an online community during the lockdown. Research in Psychotherapy: Psychopathology, Process and Outcome, 24(3), 263-274.	x												
Alcala-Sanchez, I. G. (2021). SELF-REPORTED BELIEFS ABOUT EMOTIONAL DISTRESS DURING COVID-19 QUARANTINE IN MEXICAN LAW STUDENTS AND PROFESSORS. Annals of Behavioral Medicine, 55, S411-S411. <Go to ISI>://WOS:000648922701025			x										
Alfaifi, A., et al. (2022). "The Psychological Impact of Quarantine During the COVID-19 Pandemic on Quarantined Non-Healthcare Workers, Quarantined Healthcare Workers, and Medical Staff at the Quarantine Facility in Saudi Arabia." Psychology Research and Behavior Management 15: 1259-1270.						x							x
Alhamidi, S. A. and S. M. Alyousef (2020). "Perceptions of the Phenomena of Quarantine as Experienced by Saudi Arabian COVID-19 Patients." World Family Medicine 18(12): 82-90.						x					x		
Alhazmi, A., et al. (2020). "Knowledge, attitudes and practices among people in Saudi Arabia regarding COVID-19: A cross-sectional study." Journal of Public Health Research 9(3): 345-353.							x		x				

Alhusban, A., Alzoubi, K. H., Al-Azzam, S., & Nuseir, K. Q. (2022). Evaluation of Vulnerability Factors for Developing Stress and Depression due to COVID-19 Spread and its Associated Lockdown. Clinical Practice and Epidemiology in Mental Health, 18(1) (no pagination), Article e174501792209291.			x									
Ali-Saleh, O., & Obeid, S. Compliance with COVID-19 Preventive Guidelines Among Minority Communities: the Case of Israeli Arabs. Journal of Racial and Ethnic Health Disparities. https://doi.org/10.1007/s40615-022-01344-0	x										x	
Ali, A. M., Alkhamees, A. A., Abd Elhay, E. S., Taha, S. M., & Hendawy, A. O. (2022). COVID-19-Related Psychological Trauma and Psychological Distress Among Community-Dwelling Psychiatric Patients: People Struck by Depression and Sleep Disorders Endure the Greatest Burden. Frontiers in Public Health, 9, Article 799812. https://doi.org/10.3389/fpubh.2021.799812								x				x
Alkaabi, I., Abita, M., Mahdi, Y., Ouda, A., & Malki, M. I. (2022). Knowledge, Attitude, Practices, and Sources of Information (KAPS) Toward COVID-19 During the Second Wave Pandemic Among University Population in Qatar: A Cross-Sectional Study. Frontiers in Public Health, 10, 906159.			x									
Alkhaldi, G., Aljuraiban, G. S., Alhurishi, S., De Souza, R., Lamahewa, K., Lau, R., & Alshaikh, F. (2021). Perceptions towards COVID-19 and adoption of preventive measures among the public in Saudi Arabia: a cross sectional study. BMC Public Health, 21(1), 1251.									x		x	
Alkhaldi, G., et al. (2021). BMC Public Health 21(1): 1251.									x		x	

Alkhamees, A. A., et al. (2020). "Psychological distress in quarantine designated facility during covid-19 pandemic in saudi arabia." Risk Management and Healthcare Policy 13: 3103-3120.			x		x
Alleaume, C., Verger, P., Peretti-Watel, P., & Group, C. (2021). Psychological support in general population during the COVID-19 lockdown in France: Needs and access [Research Support, Non-U.S. Gov't]. PLoS ONE [Electronic Resource], 16(5), e0251707.		x			
Allen, H. K., Cohen-Winans, S., Armstrong, K., Clark, N. C., & Ford, M. A. (2021). COVID-19 exposure and diagnosis among college student drinkers: links to alcohol use behavior, motives, and context [Research Support, Non-U.S. Gov't]. Translational Behavioral Medicine, 11(7), 1348-1353.			x	x	
Allen, S. F., Stevenson, J., Lazuras, L., & Akram, U. (2022). The role of the COVID-19 pandemic in altered psychological well-being, mental health and sleep: an online cross-sectional study. Psychology Health & Medicine, 27(2), 343-351.		x			
Almaghrabi, M. K. (2021). "Public Awareness, Attitudes, and Adherence to COVID-19 Quarantine and Isolation in Saudi Arabia." International Journal of General Medicine 14: 4395-4403.	x			x	
Almarwani, A. M., et al. (2021). "Perceived quarantine competence, attitudes and practices among the public during the coronavirus pandemic: A Saudi descriptive study." International Journal of Clinical Practice 75(7): e14242.		x			

Almayahi, Z. K. and N. Al Lamki (2022). "Psychological effects of, and compliance with, self-isolation among COVID-19 patients in South Batinah Governorate, Oman: a cross-sectional study." Egyptian Journal of Neurology, Psychiatry and Neurosurgery 58(1) (no pagination).	x					x
Almoayad, F. A., et al. (2021). "Preventive practices against COVID-19 among residents of Riyadh, Saudi Arabia." Journal of Infection in Developing Countries 15(6): 780-786.				x		
Alnamnakani, M., Alenezi, S., Temsah, H., Alothman, M., Murshid, R. E., Alonazy, H., & Alqurashi, H. (2022). OviTI - Psychosocial Impact of Lockdown on Children due to COVID-19: A Cross-Sectional Study. Clinical Practice and Epidemiology in Mental Health, 18 (no pagination), Article e174501792203210.			x			
Aloba, O. and T. Opakunle (2021). "Psychological and illness-related correlates of insomnia in mildly symptomatic Nigerian COVID-19 adult patients during self-isolation." International Medicine 3(5): 145-151.		x			x	
Alodhayani, A. A., et al. (2021). "Predictors of Mental Health Status among Quarantined COVID-19 Patients in Saudi Arabia." Healthcare 9(10).					x	x
Alomari, M. A., Alzoubi, K. H., Khabour, O. F., & Darabseh, M. Z. (2021). Sleeping habits during COVID-19 induced confinement: A study from Jordan. Heliyon, 7(12), Article e08545. https://doi.org/10.1016/j.heliyon.2021.e08545			x			

Alpers, S. E., Skogen, J. C., Maeland, S., Pallesen, S., Rabben, A. K., Lunde, L. H., & Fadnes, L. T. (2021). Alcohol Consumption during a Pandemic Lockdown Period and Change in Alcohol Consumption Related to Worries and Pandemic Measures. International Journal of Environmental Research & Public Health [Electronic Resource], 18(3), 29.		x	x
Alqudah, A., Al-Smadi, A., Oqal, M., Qnais, E. Y., Wedyan, M., Abu Gneam, M., Alnajjar, R., Alajarmeh, M., Yousef, E., & Gammoh, O. (2021). About anxiety levels and anti-anxiety drugs among quarantined undergraduate Jordanian students during COVID-19 pandemic. International Journal of Clinical Practice, 75(7), e14249.	x		
AlRasheed, M. M., et al. (2021). "The Impact of Quarantine on Sleep Quality and Psychological Distress During the COVID-19 Pandemic." Nature and Science of Sleep 13: 1037-1048.		x	x
Alremeithi, H. M., et al. (2021). "Knowledge, Attitude, and Practices Toward SARS-COV-2 Infection in the United Arab Emirates Population: An Online Community-Based Cross-Sectional Survey." Frontiers in Public Health 9.	x		
Alrubaysh, M. A., Alkahtani, A. A., Alluwaymi, Z. S., Alanazi, S. M., Alanazi, A. M., & Al Harbi, K. M. (2021). Lifestyle changes that can increase the risk of cardiovascular disease during the COVID-19 pandemic: a cross-sectional study. World Family Medicine, 19(7), 44-53. https://doi.org/10.5742/mewfm.2021.94076	x		

Alshammari, T. K., Alkhodair, A. M., Alhebshi, H. A., Rogowska, A. M., Albaker, A. B., Al-Damri, N. T., Bin Dayel, A. F., Alonazi, A. S., Alrasheed, N. M., & Alshammari, M. A. (2022). Examining Anxiety, Sleep Quality, and Physical Activity as Predictors of Depression among University Students from Saudi Arabia during the Second Wave of the COVID-19 Pandemic. International Journal of Environmental Research & Public Health [Electronic Resource], 19(10), 21.			x	x	
Alshehri, A. S., & Alghamdi, A. H. (2021). Post-traumatic Stress Disorder Among Healthcare Workers Diagnosed With COVID-19 in Jeddah, Kingdom of Saudi Arabia, 2020 to 2021. Cureus, 13(8), Article e17371. https://doi.org/10.7759/cureus.17371			x		x
Alshiha, A. A. (2021). Quarantine Hotels: Integration between Hospitality and Healthcare Services to Promote Health Behavior and Quality of Life [Research Support, Non-U.S. Gov't]. American Journal of Health Behavior, 45(6), 1059-1078.		x			
Alvarado-Aravena, C., et al. (2022). "Effect of Confinement on Anxiety Symptoms and Sleep Quality during the COVID-19 Pandemic." Behavioral Sciences 12(10).	x				
Alyahya, K. I. (2021). The impact of Saudi lockdown to counter COVID-19. International Journal of Advanced and Applied Sciences, 8(3), 78-87. https://doi.org/10.21833/ijaas.2021.03.011	x				

Ambelu, A., Birhanu, Z., Yitayih, Y., Kebede, Y., Mecha, M., Abafita, J., Belay, A., & Fufa, D. (2021). Psychological distress during the COVID-19 pandemic in Ethiopia: an online cross-sectional study to identify the need for equal attention of intervention. Annals of General Psychiatry, 20(1), Article 22. https://doi.org/10.1186/s12991-021-00344-4	x	
Amirudin, A., et al. (2021). "The impact of the covid-19 self-isolation policy on the occupations of vulnerable groups." International Journal of Environmental Research and Public Health 18(12) (no pagination).		x
Ammerman, B. A., et al. (2021). "Preliminary investigation of the association between COVID-19 and suicidal thoughts and behaviors in the U.S." Journal of Psychiatric Research 134: 32-38.	x	
Antsiferova, A. A., et al. (2022). Profilakticheskaya Meditsina 25(2): 19-25.	x	
Anunciacao, L., Portugal, A., Andrade, L., Marques, L., & Landeira-Fernandez, J. Disentangling crucial factors of the pandemic in Brazil: Effect of lockdown restrictions on mental health. Health & Social Care in the Community. https://doi.org/10.1111/hsc.13878	x	
Araujo-Hernandez, M., Garcia-Navarro, E. B., & Caceres-Titos, M. J. (2022). Dietary Behaviours of University Students during the COVID-19 Pandemic. A Comparative Analysis of Nursing and Engineering Students. Foods, 11(12), Article 1715. https://doi.org/10.3390/foods11121715	x	

Arillotta, D., Guirguis, A., Corkery, J. M., Scherbaum, N., & Schifano, F. (2021). COVID-19 Pandemic Impact on Substance Misuse: A Social Media Listening, Mixed Method Analysis. Brain Sciences, 11(7), Article 907. https://doi.org/10.3390/brainsci11070907			x										
Asare, B. Y., et al. (2021). "Mental Well-Being during COVID-19: A Cross-Sectional Study of Fly-In Fly-Out Workers in the Mining Industry in Australia." International Journal of Environmental Research & Public Health [Electronic Resource] 18(22): 22.								x					x
Ashiq, K., Ashiq, S., Bajwa, M. A., Tanveer, S., & Qayyum, M. (2020). Knowledge, attitude and practices among the inhabitants of lahore, pakistan towards the COVID-19 pandemic: An immediate online based cross-sectional survey while people are under the lockdown. Bangladesh Journal of Medical Science, 19(Special issue), S69-S76.			x										
Asieieva, Y., Sytnik, S., Babchuk, O., Heina, O., & Dementieva, K. (2022). Peculiarities of the mental state of student youth of Ukraine during quarantine restrictions. Amazonia Investiga, 11(50), 9-15. https://doi.org/10.34069/ai/2022.50.02.1			x										
Aslaner, H., et al. (2022). "Death and COVID-19 Anxiety in Home-Quarantined Individuals Aged 65 and Over During the Pandemic." Omega - Journal of Death & Dying 85(1): 246-258.	x	x											
Asmundson, G. J., et al. (2020). "Do pre-existing anxiety-related and mood disorders differentially impact COVID-19 stress responses and coping?" Journal of Anxiety Disorders Vol 74 2020, ArtID 102271 74.			x										

Astoyants, M. S., et al. (2020). "RUSSIAN POPULATION UNDER THE CONDITIONS OF SELF-ISOLATION: ANALYSIS OF PROBLEMS AND TRANSFORMATIONAL CONTRADICTIONS OF SOCIAL BEHAVIOR IN THE ASPECT OF VIRTUALITY." Laplage Em Revista 6: 152-160.	x
Atkinson, L., Kite, C., McGregor, G., James, T., Clark, C. C. T., Randeva, H. S., & Kyrou, I. (2021). Uncertainty, anxiety and isolation: Experiencing the covid-19 pandemic and lockdown as a woman with polycystic ovary syndrome (pcos). Journal of Personalized Medicine, 11(10) (no pagination), Article 952.	x
Aubert, C., & Augeraud-Veron, E. (2021). The relative power of individual distancing efforts and public policies to curb the COVID-19 epidemics [Research Support, Non-U.S. Gov't]. PLoS ONE [Electronic Resource], 16(5), e0250764.	x
Aung, M. N., Stein, C., Chen, W. T., Garg, V., Saraswati Sitepu, M., Thu, N. T. D., Gundran, C. P. D., Hassan, M. R., Suthutvoravut, U., Soe, A. N., Nour, M., Gyi, K. K., Brandl, R., & Yuasa, M. (2021). Community responses to COVID-19 pandemic first wave containment measures: a multinational study. Journal of Infection in Developing Countries, 15(8), 1107-1116.	x
Awadalla, N. J., Alsabaani, A. A., Alsaleem, M. A., Alsaleem, S. A., Alshaikh, A. A., Al-Fifi, S. H., & Mahfouz, A. A. (2022). Increased mental stress among undergraduate medical students in south-western Saudi Arabia during the COVID-19 pandemic. PeerJ, 10 (no pagination), Article e13900	x

Awais, M. A., Chaudhery, M. M., Khan, M. S., Butt, A. U. A., Malik, A. R., Khan, M. N., Khalid, A., Mahmood, M., Afzal, M. T., & Waseem, M. H. (2021). Factors contributing to distress among school and college-going adolescents during COVID-19 Lockdown: A cross-sectional study conducted in Sibi Balochistan, Pakistan. Journal of Education and Health Promotion, 10(1), Article 317. https://doi.org/10.4103/jehp.jehp_1313_20	x		
Ayhan, F., et al. (2022). "The effects of compulsory isolation measures during the COVID-19 pandemic: The example of prison workers." International Journal of Health Planning & Management 37(5): 2905-2917.		x	x
Bafunno, D., Romito, F., Lagattolla, F., Delvino, V. A., Minoia, C., Loseto, G., Dellino, M., Guarini, A., Catino, A., Montrone, M., Longo, V., Pizzutilo, P., Galetta, D., Giotta, F., Latorre, A. C., Russo, A., Lorusso, V., & Cormio, C. (2021). Psychological well-being in cancer outpatients during COVID-19. Journal of B.U.ON., 26(3), 1127-1134.	x		
Bagheri Lankarani, K., Hemyari, C., Honarvar, B., Khaksar, E., Shaygani, F., Rahmanian Haghighi, M. R., & Shaygani, M. R. (2022). Domestic violence and associated factors during COVID-19 epidemic: an online population-based study in Iran [Research Support, Non-U.S. Gov't]. BMC Public Health, 22(1), 774.	x		
Bahar Moni, A. S., et al. (2021). "Psychological distress, fear and coping among Malaysians during the COVID-19 pandemic." PLoS ONE [Electronic Resource] 16(9): e0257304.		x	x

Bahrami-Samani, S., Firouzbakht, M., Azizi, A., & Omidvar, S. (2022). Anxiety, Depression, and Predictors amongst Iranian Students Aged 8 to 18 Years during the COVID-19 Outbreak First Peak. Iranian Journal of Psychiatry, 17(2), 187-195.	x		
Bakkar, B., Mohsen, F., Armashi, H., Marrawi, M., & Aldaher, N. (2022). A cross-sectional survey of COVID-19: attitude and prevention practice among Syrians. Heliyon, 8(3), Article e09124. https://doi.org/10.1016/j.heliyon.2022.e09124	x		
Baloch, G. M., Sundarasen, S., Chinna, K., Nurunnabi, M., Kamaludin, K., Khoshaim, H. B., Hossain, S. F. A., & AlSukayt, A. (2021). COVID-19: Exploring impacts of the pandemic and lockdown on mental health of Pakistani students. PeerJ, 9 (no pagination), Article 10612.	x		
Balsamo, M., Murdock, K. K., & Carlucci, L. (2022). Psychological factors in adherence to COVID-19 public health restrictions in Italy: A path model testing depressed mood, anxiety, and co-rumination via cellphone. PLoS ONE [Electronic Resource], 17(12), e0278628.	x		
Baminiwatta, A., Dayabandara, M., De Silva, J., Gadambanathan, T., Ginige, P., Premarathne, I., Rajapaksha, H., Wickramasinghe, A., Sivayokan, S., & Wijesinghe, C. (2022). Perceived Impact of the COVID-19 Pandemic on Psychiatric Training Among Final-Year Medical Undergraduates in Sri Lanka: an Online Survey of Students from Eight Universities. Academic Psychiatry, 46(6), 729-734.		x	x

Bankovskaya, S., Maddahi, J., & Khachaki, T. L. (2021). From Isolation to Violence: Changes of the Domestic Environment in the Iranian Family under COVID-19. Sociologiceskoe Obozrenie, 20(4), 86-110. https://doi.org/10.17323/1728-192x-2021-4-86-110		x	
Bannour, R., et al. (2021). "Compliance with preventive measures before and during home quarantine among a Tunisian cohort of COVID-19 patients." Antimicrobial Resistance and Infection Control. Conference: 6th International Conference on Prevention and Infection Control, ICPIC 10(SUPPL 1).	x		x
Bara'a, H. M. I., Nori, M. M. M., Abdallah, W. S., & Ali, S. M. (2021). Coronavirus 2019-like illness and public adherence to preventive measures, Sudan 2020. Journal of Preventive Medicine & Hygiene, 62(2), E305-E310.	x		x
Barbato, M., & Thomas, J. (2021). In this together: Psychological wellbeing of foreign workers in the United Arab Emirates during the COVID-19 pandemic. International Journal of Psychology, 56(6), 825-833.		x	
Barcin-Guzeldere, H. K., & Devrim-Lanpir, A. (2022). The Association Between Body Mass Index, Emotional Eating and Perceived Stress during COVID-19 Partial Quarantine in Healthy Adults. Public Health Nutrition, 25(1), 43-50.		x	
Barone, B., De Luca, L., Napolitano, L., Reccia, P., Crocetto, F., Creta, M., Vitale, R., Caputo, V. F., Martino, R., Cirillo, L., Fusco, G. M., Trivellato, M., Celentano, G., La Rocca, R., Prezioso, D., & Longo, N. (2022). Lower urinary tract symptoms and mental health during COVID-19 pandemic. Archivio Italiano di Urologia, Andrologia, 94(1), 46-50.		x	

Bartoszek, A., Walkowiak, D., Bartoszek, A., & Kardas, G. (2020). Mental Well-Being (Depression, Loneliness, Insomnia, Daily Life Fatigue) during COVID-19 Related Home-Confinement-A Study from Poland. International Journal of Environmental Research and Public Health, 17(20), Article 7417. https://doi.org/10.3390/ijerph17207417	x
Bastoni, S., Wrede, C., Ammar, A., Braakman-Jansen, A., Sanderman, R., Gaggioli, A., Trabelsi, K., Masmoudi, L., Boukhris, O., Glenn, J. M., Bouaziz, B., Chtourou, H., & van Gemert-Pijnen, L. (2021). Psychosocial Effects and Use of Communication Technologies during Home Confinement in the First Wave of the COVID-19 Pandemic in Italy and The Netherlands. International Journal of Environmental Research & Public Health [Electronic Resource], 18(5), 05.	x
Behisi, M. A., Altaweel, H. M., Gassas, R. F., Aldehaiman, M., & Alkhamees, A. A. (2021). COVID-19 Pandemic and Mental Health Status of Saudi Citizens Living Abroad. International Journal of Environmental Research & Public Health [Electronic Resource], 18(15), 25.	x
Belasheva, I. V., Gapich, A. E., Yesayan, M. L., Polshakova, I. N., & Soloveva, E. V. (2021). PSYCHO-EMOTIONAL STATE OF STUDENTS DURING COVID-19 PANDEMIC SELF-ISOLATION PERIOD IN THE CONTEXT OF SOCIO-DEMOGRAPHIC FACTORS. Revista on Line De Politica E Gestao Educacional, 25, 981-1000. https://doi.org/10.22633/rpge.v25iesp.2.15281	x

Ben-Ezra, M., et al. (2020). "The association of being in quarantine and related COVID-19 recommended and non-recommended behaviors with psychological distress in Chinese population." Journal of Affective Disorders 275: 66-68.						x					x
Benham, J. L., et al. (2021). "Attitudes, current behaviours and barriers to public health measures that reduce COVID-19 transmission: A qualitative study to inform public health messaging." PLoS ONE 16(2 February 2021) (no pagination).								x	x		
Benke, C., Autenrieth, L. K., Asselmann, E., & Pane-Farre, C. A. (2020). Lockdown, quarantine measures, and social distancing: Associations with depression, anxiety and distress at the beginning of the COVID-19 pandemic among adults from Germany. Psychiatry Research, 293, 113462.				x							
Berard, M., Rattaz, C., Peries, M., Loubersac, J., Munir, K., & Baghdadli, A. (2021). Impact of containment and mitigation measures on children and youth with ASD during the COVID-19 pandemic: Report from the ELENA cohort [Research Support, Non-U.S. Gov't]. Journal of Psychiatric Research, 137, 73-80.	x										
Berg, M. B. and L. Lin (2020). "Prevalence and predictors of early COVID-19 behavioral intentions in the United States." Translational Behavioral Medicine 10(4): 843-849.							x		x		
Bhardwaj, P., et al. (2021). "Analysis of facility and home isolation strategies in covid 19 pandemic: Evidences from jodhpur, india." Infection and Drug Resistance 14: 2233-2239.					x						

Bianchi, D., Baiocco, R., Pompili, S., Lonigro, A., Di Norcia, A., Cannoni, E., Longobardi, E., Zammuto, M., Di Tata, D., & Laghi, F. (2022). Binge Eating and Binge Drinking in Emerging Adults During COVID-19 Lockdown in Italy: An Examination of Protective and Risk Factors. Emerging Adulthood, 10(1), 291-303. https://doi.org/10.1177/21676968211058501	x		
Bin Abdulrahman, A. K., et al. (2021). "Response of Saudi Population to Strict Preventive Measures against COVID-19." International Journal of Environmental Research & Public Health [Electronic Resource] 18(24): 20.	x		
Bin Halim, S. F., Mridha, S., Nasrin, N., Islam, M. K., & Hossain, M. T. (2022). Socioeconomic crisis and mental health stress among the middle-income group during the covid-19 pandemic. Sociological Spectrum, 42(2), 119-134. https://doi.org/10.1080/02732173.2022.2081639	x		
Bin Helayel, H., et al. (2022). "Quarantine-related traumatic stress, views, and experiences during the first wave of Coronavirus pandemic: A mixed-methods study among adults in Saudi Arabia." PLoS ONE [Electronic Resource] 17(1): e0261967.		x	x
Bivia-Roig, G., Boldo-Roda, A., Blasco-Sanz, R., Serrano-Raya, L., DelaFuente-Diez, E., Muzquiz-Barbera, P., & Lison, J. F. (2021). Impact of the COVID-19 Pandemic on the Lifestyles and Quality of Life of Women With Fertility Problems: A Cross-Sectional Study [Research Support, Non-U.S. Gov't]. Frontiers in Public Health, 9, 686115.	x		

Blbas, H. T. A., et al. (2022). "Phenomenon of depression and anxiety related to precautions for prevention among population during the outbreak of COVID-19 in Kurdistan Region of Iraq: based on questionnaire survey." Journal of Public Health-Heidelberg 30(3): 567-571.		x
Blithikioti, C., Nuno, L., Paniello, B., Gual, A., & Miquel, L. (2021). Impact of COVID-19 lockdown on individuals under treatment for substance use disorders: Risk factors for adverse mental health outcomes [Research Support, Non-U.S. Gov't]. Journal of Psychiatric Research, 139, 47-53.	x	
Bodas, M., & Peleg, K. (2020). Income assurances are a crucial factor in determining public compliance with self-isolation regulations during the COVID-19 outbreak - cohort study in Israel. Israel Journal of Health Policy Research, 9(1), 54.	x	
Bodas, M., & Peleg, K. (2020). Self-Isolation Compliance In The COVID-19 Era Influenced By Compensation: Findings From A Recent Survey In Israel. Health Affairs, 39(6), 936-941.	x	
Bodas, M., & Peleg, K. (2021). Pandemic Fatigue: The Effects Of The COVID-19 Crisis On Public Trust And Compliance With Regulations In Israel. Health Affairs, 40(8), 1225-1233.	x	
Boelen, P. A., Eisma, M. C., Smid, G. E., de Keijser, J., & Lenferink, L. I. M. (2021). Remotely Delivered Cognitive Behavior Therapy for Disturbed Grief During the COVID-19 Crisis: Challenges and Opportunities. Journal of Loss & Trauma, 26(3), 211-219. https://doi.org/10.1080/15325024.2020.1793547	x	

Bohn, L., Barros, D., Borges-Machado, F., Carrapatoso, S., Pizarro, A. N., & Carvalho, J. (2021). Active Older Adults Keep Aerobic Capacity and Experience Small Reductions in Body Strength During Confinement Due to COVID-19 Outbreak [Research Support, Non-U.S. Gov't]. Journal of Aging & Physical Activity, 29(6), 1034-1041.	x		
Boiko, II, Herus, O. I., Klymanska, L. D., Savka, V. Y., Klos, L. Y., Shkoliar, M. V., & Vitale, V. (2021). Covid-19 Pandemic Lifestyle: Students Mental Health Challenges. Wiadomosci Lekarskie, 74(11 cz 1), 2723-2727.	x		
Bokhan, T. G., Galazhinsky, E. V., Leontiev, D. A., Rasskazova, E. I., Terekhina, O. V., Ulyanich, A. L., Shabalovskaya, M. V., Bogomaz, S. A., & Vidyakina, T. A. (2021). COVID-19 AND SUBJECTIVE WELL-BEING: PERCEIVED IMPACT, POSITIVE PSYCHOLOGICAL RESOURCES AND PROTECTIVE BEHAVIOR. Psychology-Journal of the Higher School of Economics, 18(2), 259-275. https://doi.org/10.17323/1813-8918-2021-2-259-275	x		
Bonkalo, T. I., Marinova, T. Y., Feoktistova, S. V., & Shmeleva, S. V. (2020). Dyadic Coping Strategies of Spouses as a Factor in Latent Dysfunctional Relationships in the Family: an Empirical Study in a Pandemic. *Social Psychology and Society*, *11*(3), 35-50. https://doi.org/10.17759/sps.2020110303		x	x

Bonsaksen, T., Heir, T., Schou-Bredal, I., Ekeberg, O., Skogstad, L., & Grimholt, T. K. (2020). Post-Traumatic Stress Disorder and Associated Factors during the Early Stage of the COVID-19 Pandemic in Norway. *International Journal of Environmental Research & Public Health [Electronic Resource]*, *17*(24), 09.	x						x			
Book, J., Broichhaus, L., Grune, B., Niessen, J., Wiesmuller, G. A., Joisten, C., & Kossow, A. (2022). Adherence to stay-at-home orders: awareness, implementation and difficulties of officially ordered quarantine measures in the context of the COVID-19 pandemic in Cologne - a retrospective cohort study. *BMJ Open*, *12(11) (no pagination)*, Article e063358.								x		x
Bourne, T., Kyriacou, C., Shah, H., Ceusters, J., Preisler, J., Metzger, U., Landolfo, C., Lees, C., & Timmerman, D. (2022). Experiences and well-being of healthcare professionals working in the field of ultrasound in obstetrics and gynaecology as the SARS-CoV-2 pandemic were evolving: a cross-sectional survey study [Research Support, Non-U.S. Gov't]. *BMJ Open*, *12*(2), e051700.		x								
Brailovskaia, J., Schneider, S., & Margraf, J. (2021). To vaccinate or not to vaccinate!? Predictors of willingness to receive Covid-19 vaccination in Europe, the U.S., and China [Research Support, Non-U.S. Gov't]. *PLoS ONE [Electronic Resource]*, *16*(12), e0260230.		x								

Brankston, G., Merkley, E., Fisman, D. N., Tuite, A. R., Poljak, Z., Loewen, P. J., & Greer, A. L. (2021). Socio-demographic disparities in knowledge, practices, and ability to comply with COVID-19 public health measures in Canada [Research Support, Non-U.S. Gov't]. *Canadian Journal of Public Health. Revue Canadienne de Sante Publique*, *112*(3), 363-375.			x	x
Braule Pinto, A. L. C., Serpa, A. L. O., de Paula, J. J., Costa, D. S., Robis, K., Diaz, A. P., Joaquim, R. M., da Silva, A. G., de Miranda, D. M., & Malloy-Diniz, L. F. (2021). Increased risk of health professionals to feel traumatized during the COVID-19 pandemic [Research Support, Non-U.S. Gov't]. *Scientific Reports*, *11*(1), 18286.	x			
Brett-Major, D. M., Cates, D. S., Lawler, J. V., Vokoun, C., Hewlett, A. L., Johnson, D. W., Schnaubelt, E. R., Wadman, M. C., Kratochvil, C. J., & Broadhurst, M. J. (2021). Long-Term Assessment of the Effects of COVID-19 and Isolation Care on Survivor Disability and Anxiety [Research Support, Non-U.S. Gov't]. *American Journal of Tropical Medicine & Hygiene*, *105*(3), 737-739.		x		x
Bridgen, J. R. E., Jewell, C. P., & Read, J. M. (2021). Social mixing patterns in the UK following the relaxation of COVID-19 pandemic restrictions: a cross-sectional online survey. doi:10.1101/2021.10.22.21265371	x			
Brindal, E., Ryan, J. C., Kakoschke, N., Golley, S., Zajac, I. T., & Wiggins, B. (2022). Individual differences and changes in lifestyle behaviours predict decreased subjective well-being during COVID-19 restrictions in an Australian sample. *Journal of Public Health*, *44*(2), 450-456.	x			

Brocious, H., Trawver, K., & Demientieff, L. X. (2021). Managed alcohol: one community's innovative response to risk management during COVID-19. *Harm Reduction Journal*, *18*(1), 125.			x		x
Broodryk, T., & Robinson, K. (2022). Dataset describing Aotearoa New Zealand young adults' psychological well-being and behaviour during nationwide lockdown. *Data in Brief*, *40*, Article 107808. https://doi.org/10.1016/j.dib.2022.107808		x			
Bryson, H., Mensah, F., Price, A., Gold, L., Mudiyanselage, S. B., Kenny, B., Dakin, P., Bruce, T., Noble, K., Kemp, L., & Goldfeld, S. (2021). Clinical, financial and social impacts of COVID-19 and their associations with mental health for mothers and children experiencing adversity in Australia [Research Support, Non-U.S. Gov't]. *PLoS ONE [Electronic Resource]*, *16*(9), e0257357.	x				
Bu, F., Steptoe, A., & Fancourt, D. (2022). Longitudinal changes in home confinement and mental health implications: A 17-month follow-up study in england during the covid-19 pandemic. *Psychological Medicine*, No Pagination Specified.	x				
Bucak, I. H., Almis, H., Tasar, S. O., Uygun, H., & Turgut, M. (2021). Have the sleep habits in children of health workers been more affected during the COVID-19 pandemic? *Sleep Medicine*, *83*, 235-240.	x				
Buhler, A., & Willmund, G. D. (2021). Adherence and Psychosocial Well-Being During Pandemic-Associated Pre-deployment Quarantine. *Frontiers in Public Health*, *9*, 802180.				x	x

Buhr, L., Schicktanz, S., & Nordmeyer, E. (2022). Attitudes Toward Mobile Apps for Pandemic Research Among Smartphone Users in Germany: National Survey [Research Support, Non-U.S. Gov't]. *JMIR MHealth and UHealth*, *10*(1), e31857.		x	x	
Burkova, V. N., Butovskaya, M. L., Randall, A. K., Fedenok, J. N., Ahmadi, K., Alghraibeh, A. M., Allami, F. B. M., Alpaslan, F. S., Al-Zu'bi, M. A. A., Al-Mseidin, K. I. M., Bicer, D. F., Cetinkaya, H., David, O. A., Donato, S., Dural, S., Erickson, P., Ermakov, A. M., Ertugrul, B., Fayankinnu, E. A., . . . Zinurova, R. I. (2022). Factors Associated With Highest Symptoms of Anxiety During COVID-19: Cross-Cultural Study of 23 Countries. *Frontiers in Psychology*, *13*, Article 805586. https://doi.org/10.3389/fpsyg.2022.805586	x			
Buselli, R., Corsi, M., Veltri, A., Baldanzi, S., Chiumiento, M., Del Lupo, E., Marino, R., Necciari, G., Caldi, F., Perretta, S., Foddis, R., Guglielmi, G., & Cristaudo, A. (2022). Quarantine and Mental Health Challenges for Occupational Medicine: The Case Report of a Nurse Infected With SARS-CoV-2 [Case Reports]. *Workplace Health & Safety*, *70*(1), 43-49.		x	x	
Buyukbayram, Z., Aksoy, M., & Sayan, G. (2022). Analysis of the Spiritual Orientations and the Hopelessness Levels of the Patients Diagnosed with COVID-19: A Cross-Sectional Study. *Florence Nightingale Journal of Nursing*, *30*(2), 182-189. https://doi.org/10.54614/fnjn.2022.21068		x		x

Byun, J. A., Sim, T. J., Lim, T. Y., Jang, S. I., & Kim, S. H. (2022). Association of compliance with COVID-19 public health measures with depression [Research Support, Non-U.S. Gov't]. *Scientific Reports*, *12*(1), 13464.	x
Cabot, J., & Bushnik, T. (2022). Compliance with precautions to reduce the spread of COVID-19 in Canada. *Health Reports*, *33*(9), 3-10.	x
Cai, S., Zhang, T., Robin, C., Sawyer, C., Rice, W., Smith, L. E., Amlot, R., Rubin, G. J., Yardley, L., Hickman, M., Oliver, I., & Lambert, H. (2022). Learning about COVID-19 across borders: public health information and adherence among international travellers to the UK. *Public Health*, *203*, 9-14.	x
Cai, X., Hu, X. P., Ekumi, I. O., Wang, J. C., An, Y. W., Li, Z. W., & Yuan, B. (2020). Psychological Distress and Its Correlates Among COVID-19 Survivors During Early Convalescence Across Age Groups. *American Journal of Geriatric Psychiatry*, *28*(10), 1030-1039. https://doi.org/10.1016/j.jagp.2020.07.003	x
Cakmak, G., Ozturk, E., & Ozturk, Z. A. (2021). Being a caregiver in the palliative care unit in the pandemic: who can do it better? *Iranian Red Crescent Medical Journal*, *23(8) (no pagination)*, Article e950.	x
Caliskan, F., & Dost, B. (2020). The evaluation of knowledge, attitudes, depression and anxiety levels among emergency physicians during the COVID-19 pandemic. *Signa Vitae*, *16(1)*, 163-171.	x

Cansel, N., Ucuz, I., Arslan, A. K., Tetik, B. K., Colak, C., Melez, S. N. I., Gumustakim, R. S., Ceylan, S., Ozturk, G. Z., Ozturk, Y. K., Cadirci, D., & Akca, A. S. D. (2021). Prevalence and predictors of psychological response during immediate COVID-19 pandemic. *International Journal of Clinical Practice*, *75*(5), Article e13996. https://doi.org/10.1111/ijcp.13996		x				
Caralis, P. (2021). The Use of Telemedicine for Patients with Mental Illness and COVID-19 Infection. *Psychiatric Annals*, *51*(6), 283-286. https://doi.org/10.3928/00485713-20210510-01		x				
Cardenal-Munoz, E., Nabbout, R., Boronat, S., Lara-Herguedas, J., Villanueva, V., & Aibar, J. A. (2021). Impact of COVID-19 on Spanish patients with Dravet syndrome and their caregivers: consequences of lockdown [Comment]. *Revista de Neurologia*, *73*(2), 57-65.		x				
Cardwell, K., O'Neill, S. M., Tyner, B., Broderick, N., O'Brien, K., Smith, S. M., Harrington, P., Ryan, M., & O'Neill, M. (2022). A rapid review of measures to support people in isolation or quarantine during the Covid-19 pandemic and the effectiveness of such measures [Research Support, Non-U.S. Gov't Review]. *Reviews in Medical Virology*, *32*(1), e2244.				x		
Carlsen, E. O., Caspersen, I. H., Trogstad, L., Gjessing, H., & Magnus, P. (2020). Public adherence to governmental recommendations regarding quarantine and testing for COVID-19 in two Norwegian cohorts. doi:10.1101/2020.12.18.20248405	x					x

Carlucci, L., D'Ambrosio, I., & Balsamo, M. (2020). Demographic and attitudinal factors of adherence to quarantine guidelines during COVID-19: The Italian model. *Frontiers in Psychology Vol 11 2020, ArtID 559288*, *11*.	x			
Carter, H., Weston, D., Greenberg, N., Oliver, I., Robin, C., Rubin, G. J., Wessely, S., Gauntlett, L., & Amlot, R. (2021). Experiences of supported isolation in returning travellers during the early COVID-19 response: a qualitative interview study [Research Support, Non-U.S. Gov't]. *BMJ Open*, *11*(7), e050405.		x	x	
Cates, D. S. (2021). Minimizing Psychological Distress and Promoting Resilience During Quarantine: Piloting the Town Hall Model. *Professional Psychology-Research and Practice*, *52*(3), 260-271. https://doi.org/10.1037/pro0000382		x	x	
Cebrian-Cuenca, A., Mira, J. J., Caride-Miana, E., Fernandez-Jimenez, A., & Orozco-Beltran, D. (2021). Sources of psychological distress among primary care physicians during the COVID-19 pandemic's first wave in Spain: a cross-sectional study [Research Support, Non-U.S. Gov't]. *Primary Health Care Research & Development*, *22*, e55.	x			
Cekic, S., Karali, Z., Cicek, F., Canitez, Y., & Sapan, N. (2021). The Impact of the COVID-19 Pandemic in Adolescents with Asthma. *Journal of Korean Medical Science*, *36*(49), e339.	x			
Cetinkaya, S., Todil, T., & Kara, M. (2022). Future anxiety and coping methods of nursing students during COVID-19 pandemic: A cross-sectional study. *Medicine*, *101*(9), e28989.		x		x

Chacon, F. R., Doval, J. M., Rodriguez, V. I., Quintero, A., Mendoza, D. L., Mejia, M. D., Omana, O. D., Contreras, M. B., Gasparini, S., Gonzalez, C. J., Camejo-Avila, N. A., Romero, S. R., Flora-Noda, D. M., Maricuto, A. L., Velasquez, V. L., Guevara, R. N., Carballo, M., Caldera, J., Redondo, M. C., . . . Forero-Pena, D. A. (2021). Knowledge, attitudes, and practices related to COVID-19 among patients at Hospital Universitario de Caracas triage tent: A cross-sectional study. *Biomedica*, *41*(Sp. 2), 48-61.		x		
Chakeri, A., Jalali, E., Ghadi, M. R., & Mohamadi, M. (2020). Evaluating the effect of nurse-led telephone follow-ups (tele-nursing) on the anxiety levels in people with coronavirus. *Journal of Family Medicine and Primary Care*, *9*(10), 5351-5354. https://doi.org/10.4103/jfmpc.jfmpc_847_20	x		x	
Chakma, T., Thomas, B. E., Kohli, S., Moral, R., Menon, G. R., Periyasamy, M., Venkatesh, U., Kulkarni, R. N., Prusty, R. K., Balu, V., Grover, A., Kishore, J., Viray, M., Venkateswaran, C., Mathew, G., Ketharam, A., Balachandar, R., Singh, P. K., Jakhar, K., . . . Panda, S. (2021). Psychosocial impact of COVID-19 pandemic on healthcare workers in India & their perceptions on the way forward - A qualitative study [Research Support, Non-U.S. Gov't]. *Indian Journal of Medical Research*, *153*(5&6), 637-648.			x	x

Chan, A. Y., Ting, C., Chan, L. G., & Hildon, Z. J. L. (2022). "The emotions were like a roller-coaster": a qualitative analysis of e-diary data on healthcare worker resilience and adaptation during the COVID-19 outbreak in Singapore. *Human Resources for Health*, *20*(1), Article 60. https://doi.org/10.1186/s12960-022-00756-7		x			x
Chan, L. G., Tan, P. L. L., Sim, K., Tan, M. Y., Goh, K. H., Su, P. Q., Tan, A. K. H., Lee, E. S., Tan, S. Y., Lim, W. P., Aw, C. H., Goh, Y. Z., Sadarangani, S., & Chow, A. (2021). Psychological impact of repeated epidemic exposure on healthcare workers: findings from an online survey of a healthcare workforce exposed to both SARS (severe acute respiratory syndrome) and COVID-19. *BMJ Open*, *11*(11), e051895.	x				
Chandra, M., Rai, C. B., Sandhu, V. K., Kumari, N., Vishnoi, S., Aman, S., & Gautam, N. (2021). Mhealth based mental health support counselling service for covid-19 suspect and positive patients in isolation facilities. *Journal, Indian Academy of Clinical Medicine*, *22(1-2)*, 6-11.		x	x		
Chandra, R., Kumar, S., Supehia, S., Das, A., & Agarwal, D. (2022). Psychological distress and well-being assessment among Indian people during COVID-19 pandemic. *Journal of Family Medicine and Primary Care*, *11*(4), 1341-1347. https://doi.org/10.4103/jfmpc.jfmpc_1203_21		x		x	

Chandran, N., Vinuprasad, V. G., Sreedevi, C., Sathiadevan, S., & Deepak, K. S. (2022). COVID-19-related Stigma Among the Affected Individuals: A Cross-Sectional Study From Kerala, India. *Indian Journal of Psychological Medicine*, *44*(3), 279-284. https://doi.org/10.1177/02537176221086983	x		x	
Chandran, P., Lilabi, M. P., Thomas, B., George, B., Hafeez, N., George, A. M., & Cyriac, M. E. (2022). COVID-19 infection among health care workers in a tertiary care teaching hospital in Kerala - India. *Journal of Family Medicine and Primary Care*, *11*(1), 245-250. https://doi.org/10.4103/jfmpc.jfmpc_1095_21	x	x		
Chang, D. G., Park, J. B., Baek, G. H., Kim, H. J., Bosco, A., Hey, H. W. D., & Lee, C. K. (2020). The impact of COVID-19 pandemic on orthopaedic resident education: a nationwide survey study in South Korea. *International Orthopaedics*, *44*(11), 2203-2210.	x	x		
Chatterjee, S. S., Chakrabarty, M., Banerjee, D., Grover, S., Chatterjee, S. S., & Dan, U. (2021). Stress, sleep and psychological impact in healthcare workers during the early phase of COVID-19 in India: A factor analysis. *Frontiers in Psychology Vol 12 2021, ArtID 611314*, *12*.	x			x
Chen, D., Song, F., Tang, L., Zhang, H., Shao, J., Qiu, R., Wang, X., & Ye, Z. (2020). Quarantine experience of close contacts of COVID-19 patients in China: A qualitative descriptive study. *General Hospital Psychiatry*, *66*, 81-88.	x		x	

Chen, H., Zhao, X., Li, L., Yan, S. Y., Shang, X. F., & Li, X. H. (2022). Mental health and physical symptoms of people quarantined during the COVID-19 outbreak. *Journal of Infection*, *84*(1), E11-E12. https://doi.org/10.1016/j.jinf.2021.09.009			x	x
Chen, L. L., Wang, D. D., Xia, Y. X., & Zhou, R. L. (2022). The Association Between Quarantine Duration and Psychological Outcomes, Social Distancing, and Vaccination Intention During the Second Outbreak of COVID-19 in China. *International Journal of Public Health*, *67*, Article 1604096. https://doi.org/10.3389/ijph.2022.1604096	x			
Chen, L. L., Zhao, H., Razin, D., Song, T. T., Wu, Y., Ma, X. P., Aji, H., Wang, G., Wang, M. L., & Yan, L. (2021). Anxiety levels during a second local COVID-19 pandemic breakout among quarantined people: A cross sectional survey in China. *Journal of Psychiatric Research*, *135*, 37-46. https://doi.org/10.1016/j.jpsychires.2020.12.067	x			
Chen, W. T., Ju, Y. M., Liu, B. S., Huang, M., Yang, A. P., Zhou, Y., Wang, M., Liao, M., Shu, K. L., Liu, J. Y., & Zhang, Y. (2021). Negative Appraisals of the COVID-19 Social Impact Associated With the Improvement of Depression and Anxiety in Patients After COVID-19 Recovery. *Frontiers in Psychiatry*, *12*, Article 585537. https://doi.org/10.3389/fpsyt.2021.585537		x		

Chen, X., Xu, Q., Lin, H., Zhu, J., Chen, Y., Zhao, Q., Fu, C., & Wang, N. (2021). Quality of life during the epidemic of COVID-19 and its associated factors among enterprise workers in East China. *BMC Public Health*, *21*(1), 1370.	x			
Chen, Y. L., Jin, J. H., Zhang, X. Y., Zhang, Q., Dong, W. Z., & Chen, C. (2021). Reducing Objectification Could Tackle Stigma in the COVID-19 Pandemic: Evidence From China. *Frontiers in Psychology*, *12*, Article 664422. https://doi.org/10.3389/fpsyg.2021.664422	x			
Chen, Y., Zhang, Z. H., & Wang, T. (2022). Dire Straits: How tourists on the Diamond Princess cruise endured the COVID-19 crisis. *Tourism Management*, *91*, Article 104503. https://doi.org/10.1016/j.tourman.2022.104503		x		x
Cheng, H. J., Liao, C. S., Huang, Y. W., & Li, C. Y. (2023). Associations between psychological responses and quality of life at early and late time of quarantine among residents of a collective quarantine facility in central Taiwan. *Journal of Psychosomatic Research*, *164 (no pagination)*, Article 111076.		x		x
Cheng, H. Y., Chueh, Y. N., Chen, C. M., Jian, S. W., Lai, S. K., & Liu, D. P. (2021). Taiwan's COVID-19 response: Timely case detection and quarantine, January to June 2020. *Journal of the Formosan Medical Association*, *120(6)*, 1400-1404.		x	x	
Chew, C. C., Lim, X. J., Chang, C. T., Rajan, P., Nasir, N., & Low, W. Y. (2021). Experiences of social stigma among patients tested positive for COVID-19 and their family members: a qualitative study. *BMC Public Health*, *21*(1), 1623.		x		x

Chew, K. S., Ibrahim, N., Fazillah, N. A. M., Subramaniam, D., Sibey, A. V., Silaiman, F. N., Woa, I. M., & Rukyno, N. A. (2022). Lived experiences and coping responses toward mandatory quarantine among Malaysian healthcare workers during COVID-19 pandemic: A qualitative analysis. *Geografia-Malaysian Journal of Society & Space*, *18*(2), 200-209. https://doi.org/10.17576/geo-2022-1802-15		x	x
Chidiac, M., Ross, C., Marston, H. R., & Freeman, S. (2022). Age and Gender Perspectives on Social Media and Technology Practices during the COVID-19 Pandemic. *International Journal of Environmental Research & Public Health [Electronic Resource]*, *19*(21), 27.	x		
Chin, I., Karb, R., & Goldman, R. E. (2022). Rhode Island's Response for COVID-Positive Individuals Experiencing Homelessness and Housing Insecurity: A Qualitative Process Evaluation. *Journal of Health Care for the Poor & Underserved*, *33*(2), 857-869.	x		
Chudzicka-Czupala, A., Chiang, S. K., Grabowski, D., Zywiolek-Szeja, M., Quek, M., Pudelek, B., Teopiz, K., Ho, R., & McIntyre, R. S. (2022). Predictors of Psychological Distress across Three Time Periods during the COVID-19 Pandemic in Poland. *International Journal of Environmental Research & Public Health [Electronic Resource]*, *19*(22), 21.	x		
Chung, M. C., Wang, Y. B., Wu, X. L., Wang, N., Liu, F. S., Ye, Z. L., & Peng, T. Comparison between emerging adults and adults in terms of contamination fear, post-COVID-19 PTSD and psychiatric comorbidity. *Current Psychology*. https://doi.org/10.1007/s12144-022-03719-2	x		

Chuyko, H., Koltunovych, T., Chaplak, Y., & Komisaryk, M. (2022). Students' Defense Mechanisms and Coping Strategies in Terms of COVID-19 Pandemic. *Revista Romaneasca Pentru Educatie Multidimensionala*, *14*(1), 115-138. https://doi.org/10.18662/rrem/14.1/510		x		x
Cineka, A., & Raj, J. M. (2020). Dance and Music as a Therapy to Heal Physical and Psychological Pain: An Analytical Study of COVID-19 Patients during Quarantine. *European Journal of Molecular and Clinical Medicine*, *7(6)*, 99-109.		x	x	
Codreanu, T. A., Ngeh, S., Trewin, A., & Armstrong, P. K. (2021). Successful control of an onboard COVID-19 outbreak using the cruise ship as a quarantine facility, Western Australia, Australia. *Emerging Infectious Diseases*, *27(5)*, 1279-1287.	x			
Cohen-Louck, K. (2022). Differences in post-traumatic growth: Individual quarantine, COVID-19 duration and gender. *Frontiers in Psychology*, *13*, Article 920386. https://doi.org/10.3389/fpsyg.2022.920386	x			
Colak, S., Gurlek, B., Onal, O., Yilmaz, B., & Hocaoglu, C. (2021). The level of depression, anxiety, and sleep quality in pregnancy during coronavirus disease 2019 pandemic. *Journal of Obstetrics & Gynaecology Research*, *47*(8), 2666-2676.	x			
Collaborative, T. M. G. H. G. C. (2021). Psychological Impacts and Post-Traumatic Stress Disorder among People under COVID-19 Quarantine and Isolation: A Global Survey [Multicenter Study]. *International Journal of Environmental Research & Public Health [Electronic Resource]*, *18*(11), 26.		x	x	

Collins, H. N., Golden, M. R., Kubiak, R. W., Avoundjian, T., Kern, E., Meacham, E., Baldwin, M., Stewart, S., & Hood, J. (2022). EVALUATION of the COVID-19 CONTACT TRACING PROGRAM in KING COUNTY, WA, USA [Conference Abstract]. *Topics in Antiviral Medicine*, *30(1 SUPPL)*, 347.	x		
Cooper, A. B., Pauletti, R. E., & DiDonato, C. A. (2020). You, me, and no one else: Degree of social distancing and personality predict psychological wellness and relationship quality during the COVID-19 pandemic (Pre-Print). PsyArXiv Preprints.	x		
Costantini, A., & Mazzotti, E. (2020). Italian validation of CoViD-19 Peritraumatic Distress Index and preliminary data in a sample of general population [Validation Study]. *Rivista di Psichiatria*, *55*(3), 145-151. 8	x		
Costenaro, P., Di Chiara, C., Boscolo, V., Barbieri, A., Tomasello, A., Cantarutti, A., Cozzani, S., Liberati, C., Oletto, S., Giaquinto, C., & Dona, D. (2022). Perceived Psychological Impact on Children and Parents of Experiencing COVID-19 Infection in One or More Family Members. *Children-Basel*, *9*(9), Article 1370. https://doi.org/10.3390/children9091370		x	x
Csikos, G., Törő, K. D., Mokos, J., Sándor, R., Éva, H., Andrea, K., & Rita, F. (2020). Examining perceptions of stress, wellbeing and fear among Hungarian adolescents and their parents under lockdown during the COVID-19 pandemic.	x		

Cui, X. L., He, Y. Q., Gong, J. B., Luo, X. R., & Liu, J. B. (2021). Epidemiology of Sleep Disturbances and Their Effect on Psychological Distress During the COVID-19 Outbreak: A Large National Study in China. *Frontiers in Psychology*, *12*, Article 615867. https://doi.org/10.3389/fpsyg.2021.615867	x												
Curtis, S. J., Trewin, A., McDermott, K., Were, K., Walczynski, T., Notaras, L., & Walsh, N. (2022). An outdoor hotel quarantine facility model in Australia: best practice with optimal outcomes. *Australian & New Zealand Journal of Public Health*, *46*(5), 633-639.	x												
Czeisler, M. E., Howard, M. E., Robbins, R., Barger, L. K., Facer-Childs, E. R., Rajaratnam, S. M. W., & Czeisler, C. A. (2021). Early public adherence with and support for stay-at-home COVID-19 mitigation strategies despite adverse life impact: a transnational cross-sectional survey study in the United States and Australia. *BMC Public Health*, *21*(1), 503.	x												
d'Andrea, V., Gallotti, R., Castaldo, N., & De Domenico, M. (2022). Individual risk perception and empirical social structures shape the dynamics of infectious disease outbreaks. *PLoS Computational Biology*, *18*(2), e1009760.							x		x				
D'Onise, K., Meena, S., Venugopal, K., Currie, M., Kirkpatrick, E., Hurley, J., Nolan, R., Brayley, J., Atherton, B., & Spurrier, N. (2021). Holistic approach supporting mental wellbeing of people in enforced quarantine in South Australia during the COVID-19 pandemic. *Australian & New Zealand Journal of Public Health*, *45*(4), 325-329.						x					x		

Daly, Z., Slemon, A., Richardson, C. G., Salway, T., McAuliffe, C., Gadermann, A. M., Thomson, K. C., Hirani, S., & Jenkins, E. K. (2021). Associations between periods of COVID-19 quarantine and mental health in Canada [Research Support, Non-U.S. Gov't]. *Psychiatry Research*, *295*, 113631.	x			x		
Danilevska, N. V. (2022). Classification of stressful factors associated with COVID-19 pandemic and quarantine among Ukrainian military personnel. *Zaporozhye Medical Journal*, *24*(1), 56-60. https://doi.org/10.14739/2310-1210.2022.1.240366		x				
Danilina, N. O., Klimenko, A. S., Gulova, S., Seryapov, V. A., & Nazyuta, S. V. (2020). Mass hysteria and incidence rate control in the organized groups (RUDN University approach). *Rudn Journal of Sociology-Vestnik Rossiiskogo Universiteta Druzhby Narodov Seriya Sotsiologiya*, *20*(4), 929-938. https://doi.org/10.22363/2313-2272-2020-20-4-929-938			x			
Daoust, J. F. (2020). Elderly people and responses to COVID-19 in 27 Countries. *PLoS ONE [Electronic Resource]*, *15*(7), e0235590.				x	x	
Das, S., Goel, N. K., Tyagi, S., Paul, F. A., Gaga, J. K., Bhandari, P., Kaur, S., Rohilla, R., & Grover, P. K. (2022). Stress, Coping, and Resilience Among Home-Quarantined Persons During the COVID-19 Pandemic: Findings From a Community Study. *The Primary Care Companion to CNS Disorders*, *24*(2), 24.				x		x

Davis, A., Munari, S., Doyle, J., Sutton, B., Cheng, A., Hellard, M., & Gibbs, L. (2022). Quarantine preparedness - the missing factor in COVID-19 behaviour change? Qualitative insights from Australia [Research Support, Non-U.S. Gov't]. *BMC Public Health*, *22*(1), 1806.						x					x		
Dawes, J., May, T., Fancourt, D., & Burton, A. (2022). The impact of the COVID-19 pandemic on people experiencing homelessness: a qualitative interview study in the UK. *Lancet (London, England)*, *400(Supplement 1)*, S35.	x												
De Ridder, D., Sandoval, J., Vuilleumier, N., Stringhini, S., Spechbach, H., Joost, S., Kaiser, L., & Guessous, I. (2020). Geospatial digital monitoring of COVID-19 cases at high spatiotemporal resolution [Note]. *The Lancet Digital Health*, *2(8)*, e393-e394.					x								
Demerdash, H. M., Omar, E., & Arida, E. (2021). Evaluation of copeptin and psychological stress among healthcare providers during COVID-19 pandemic. *Egyptian Journal of Anaesthesia*, *37(1)*, 227-233.						x							x
Demirdogen, E. S., Orak, I., Cansever, O. M., Warikoo, N., & Yavuz, M. (2022). The associations between metacognition problems, mindfulness, and internalizing symptoms in university students quarantined due to Covid-19 in Turkey. *Perspectives in Psychiatric Care*, *58*(2), 560-567.						x					x		
Denford, S., Martin, A. F., Love, N., Ready, D., Oliver, I., Amlot, R., Yardley, L., & Rubin, G. J. (2021). Engagement With Daily Testing Instead of Self-Isolating in Contacts of Confirmed Cases of SARS-CoV-2: A Qualitative Analysis. *Frontiers in Public Health*, *9*, Article 714041. https://doi.org/10.3389/fpubh.2021.714041						x			x				

Denford, S., Morton, K. S., Lambert, H., Zhang, J., Smith, L. E., Rubin, G. J., Cai, S. H., Zhang, T. T., Robin, C., Lasseter, G., Hickman, M., Oliver, I., & Yardley, L. (2021). Understanding patterns of adherence to COVID-19 mitigation measures: a qualitative interview study. *Journal of Public Health*, *43*(3), 508-516. https://doi.org/10.1093/pubmed/fdab005			x	x		
Denford, S., Morton, K., Horwood, J., de Garang, R., & Yardley, L. (2021). Preventing within household transmission of Covid-19: is the provision of accommodation to support self-isolation feasible and acceptable? [Research Support, Non-U.S. Gov't]. *BMC Public Health*, *21*(1), 1641.		x		x		
Denford, S., Towler, L., Ali, B., Treneman-Evans, G., Bloomer, R., Peto, T. E. A., Young, B. C., & Yardley, L. (2022). Feasibility and acceptability of daily testing at school as an alternative to self-isolation following close contact with a confirmed case of COVID-19: a qualitative analysis. *BMC Public Health*, *22*(1), Article 742. https://doi.org/10.1186/s12889-022-13204-x			x	x		
Deng, Y. H., Shi, L. S., Li, J. X., Jiang, Z. P., Xie, C. J., Luo, S. L., Ling, L., Lin, H. L., Chen, Z. Q., Zhao, Y. L., Kang, L., Yuan, J., & Wen, W. P. (2021). The Trends of Psychological Status of People Entering from High-Risk Areas of COVID-19 Coronavirus During the Quarantine in Dedicated Hotels: A Longitudinal Survey Study from Guangzhou, China. *Risk Management and Healthcare Policy*, *14*, 5005-5014. https://doi.org/10.2147/rmhp.S331735	x					x

Deng, Y., & Yang, J. (2021). Psychological Status of Frontline Healthcare Professionals at the Outbreak of COVID-19 in Wuhan: A Narrative Case Series. *Psychiatry and Clinical Psychopharmacology*, *31(2)*, 233-237.	x			
Deng, Y., Li, H. M., & Park, M. (2022). Emotional Experiences of COVID-19 Patients in China: A Qualitative Study. *International Journal of Environmental Research and Public Health*, *19*(15), Article 9491. https://doi.org/10.3390/ijerph19159491		x		
Deng, Y., Wang, L., Yang, J., Xie, L., & Chen, Y. (2021). How COVID-19 patient narratives concerning reinfection mirror their mental health: A case series. *Psychiatria Danubina*, *33*(1), 114-119.		x		
Deng, Y., Yang, J., & Wan, W. (2021). Embodied metaphor in communication about lived experiences of the COVID-19 pandemic in Wuhan, China [Research Support, Non-U.S. Gov't]. *PLoS ONE [Electronic Resource]*, *16*(12), e0261968.	x			
Desdiani, D., Sutarto, A. P., Kharisma, A. N., Safitri, H., Hakim, A. F., & Rusyda, S. H. (2021). Sequelae in COVID-19 patients 3 months after hospital discharge or completion of self-isolation [Letter]. *Health Science Reports*, *4(4) (no pagination)*, Article e444.		x		
Desie, Y., Habtamu, K., Asnake, M., Gina, E., & Mequanint, T. (2021). Coping strategies among Ethiopian migrant returnees who were in quarantine in the time of COVID-19: a center-based cross-sectional study. *BMC psychology*, *9*(1), 192.			x	x

Dey, R. K., Mansoor, S., Hilmy, A. I., Moosa, S., Rahman, S. A., Latheef, R., Rasheed, N., Hassan, F. G., Zaadhee, A., Ibrahim, A., & Usman, S. K. (2022). Emotional distress in COVID-19 patients in Maldives. *BMC Psychiatry*, *22*(1), 184.		x											
Dharra, S., Kumar, R., 2021. Promoting Mental Health of Nurses During the Coronavirus Pandemic: Will the Rapid Deployment of Nurses' Training Programs During COVID-19 Improve Self-Efficacy and Reduce Anxiety? Cureus Journal of Medical Science. 13, e15213. https://doi.org/10.7759/cureus.15213.						x							x
Diaz Hernandez, L., Giezendanner, S., Fischer, R., Zeller, A., 2021. The effect of COVID-19 on mental well-being in Switzerland: a cross-sectional survey of the adult Swiss general population. BMC Family Practice. 22, 181.	x												
Diaz-Quijano, F. A., Ribeiro, T. B., Da Rosa, A. V., Reis, R., Aith, F., & Ventura, D. F. L. (2021). The impact of legislation on Covid-19 mortality in a Brazilian federative unit was mediated by social isolation. doi:10.1101/2021.06.16.21259057	x												
Diaz, C.E.O., Chacha, K.A.G., Ramos, E.L.H., Castillo, J.P.P., Vizuete, J.R.C., 2021. THE RIGHT TO HEALTH AND THE NON-COMPLIANCE WITH THE ISOLATION OF COVID-19 INFECTED PERSONS. Revista Universidad Y Sociedad. 13, 490-494.					x								
Diaz, F., Henriquez, P.A., 2021. Social sentiment segregation: Evidence from Twitter and Google Trends in Chile during the COVID-19 dynamic quarantine strategy. Plos One. 16, e0254638. https://doi.org/10.1371/journal.pone.0254638.	x												

Dienes, K.A., Kyle, R., Griffiths, S., Davies, A., Isherwood, K., Bailey, J., Williams, S., 2022. Public Perceptions of Test Trace Protect Wales: Understanding and Improving Self-Isolation Adherence. Psychosomatic Medicine. 84(5), A79.		x	x	
Dincer, O., Gillanders, R., 2021. Shelter in place? Depends on the place: Corruption and social distancing in American states. Social Science & Medicine. 269, 113569.	x			
Dinger, U., Morschek, L., Stangl, L., Israel, D., Schopper, A., Thanbichler, E., Zumbaum-Fischer, F., Hippchen, T., Merle, U., Tarbet, K., Nikendei, C., 2022. Psychosomatic-psychotherapeutic support for Covid-19 patients in domestic quarantine: a content analysis of supportive telephone calls. [German]. Zeitschrift fur Psychosomatische Medizin und Psychotherapie. 68(3), 283-296.		x		x
Dinic, B.M., Bodroza, B., 2021. COVID-19 protective behaviors are forms of prosocial and unselfish behaviors. Frontiers in Psychology Vol 12 2021, ArtID 647710. 12.	x			
Ditekemena, J.D., Nkamba, D.M., Muhindo, H.M., Siewe, J.N.F., Luhata, C., Van den Bergh, R., Tshefu Kitoto, A., Van Damme, W., Muyembe, J.J., Colebunders, R., 2021. Factors associated with adherence to COVID-19 prevention measures in the Democratic Republic of the Congo (DRC): results of an online survey. BMJ Open. 11, e043356.	x			

Djillali, S., Ouandelous, N.-N., Zouani, N., Crettaz Von Roten, F., de Roten, Y., 2022. Incidence and predictors of peri-traumatic reactions in an Algerian population faced with COVID-19. Annales Medico-Psychologiques. 180, S23-S28.			x												
Domenghino	x	x													
Dowthwaite, L., Fischer, J., Perez Vallejos, E., Portillo, V., Nichele, E., Goulden, M., McAuley, D., 2021. Public Adoption of and Trust in the NHS COVID-19 Contact Tracing App in the United Kingdom: Quantitative Online Survey Study. Journal of Medical Internet Research. 23, e29085.	x										x				
Droste, M. C., Stock, J., & Atkeson, A. (2020). Economic Benefits of COVID-19 Screening Tests. doi:10.1101/2020.10.22.20217984							x								
Duc, N.T.M., Alhady, ..., Mumtaz, S.U., Collaborative, T.M.G.H.G.C., 2021. Psychological Impacts and Post-Traumatic Stress Disorder among People under COVID-19 Quarantine and Isolation: A Global Survey. International Journal of Environmental Research and Public Health. 18, 5719. https://doi.org/10.3390/ijerph18115719.			x												
Duong-Quy, S., Tran-Duc, S., Hoang-Chau-Bao, D., Bui-Diem, K., Vu-Tran-Thien, Q., Nguyen-Nhu, V., 2022. Tiredness, depression, and sleep disorders in frontline healthcare workers during COVID-19 pandemic in Vietnam: A field hospital study. Frontiers in Psychiatry. 13 (no pagination), 984658.								x							x

Dyer, M.L., Sallis, H.M., Khouja, J.N., Dryhurst, S., Munafo, M.R., Associations between COVID-19 risk perceptions and mental health, wellbeing, and risk behaviours. Journal of Risk Research. https://doi.org/10.1080/13669877.2022.2127849.	x			
Ebert, C., Steinert, J.I., 2021. Prevalence and risk factors of violence against women and children during COVID-19, Germany. Bulletin of the World Health Organization. 99, 429-438.		x	x	
Ebrahimi, O.V., Bauer, D.J., Hoffart, A., Johnson, S.U., 2022. A critical period for pandemic adaptation: The evolution of depressive symptomatology in a representative sample of adults across a 17-month period during COVID-19. Journal of Psychopathology and Clinical Science. 131, 881-894.		x		x
Eichenberg, C., Grossfurthner, M., Andrich, J., Hubner, L., Kietaibl, S., Holocher-Benetka, S., 2021. The Relationship Between the Implementation of Statutory Preventative Measures, Perceived Susceptibility of COVID-19, and Personality Traits in the Initial Stage of Corona-Related Lockdown: A German and Austrian Population Online Survey. Frontiers in Psychiatry. 12 (no pagination), 596281.	x			

Ekpenyong, B.N., Abu, E.K., Langsi, R., Osuagwu, U.L., Oloruntoba, R., Ovenseri-Ogbomo, G., Timothy, C.G., Charwe, D.D., Nwaeze, O., Goson, C.P., Miner, C.A., Ishaya, T., Mashige, K.P., Agho, K.E., 2022. Public awareness and perception towards COVID-19 in Sub-Saharan African countries during the lockdown. Health Promotion Perspectives. 12, 200-211. https://doi.org/10.34172/hpp.2022.25.		x	x	
Ekpenyong, B.N., Osuagwu, U.L., Miner, C.A., Ovenseri-Ogbomo, G.O., Abu, E.K., Goson, P.C., Langsi, R., Nwaeze, O., Chikasirimobi, T.G., Charwe, D.D., Mashige, K.P., Oloruntoba, R., Ishaya, T., Agho, K.E., 2021. Knowledge, Attitudes, and Perceptions of COVID-19 among Healthcare and Non-Healthcare Workers in Sub-Saharan Africa: A Web-Based Survey. Health Security. 19, 393-404. https://doi.org/10.1089/hs.2020.0208.		x	x	
El Keshky, M.E.S., Alsabban, A.M., Basyouni, S.S., 2021. The psychological and social impacts on personal stress for residents quarantined for COVID-19 in Saudi Arabia. Archives of Psychiatric Nursing. 35, 311-316.		x		x
Elaraby, A., Shahein, M., Bekhet, A.H., Perrin, P.B., Gorgey, A.S., 2022. The COVID-19 pandemic impacts all domains of quality of life in Egyptians with spinal cord injury: a retrospective longitudinal study. Spinal Cord. 60, 757-762.	x		x	

Elgendy, M.O., Abdelrahman, M.A., Osama, H., El-Gendy, A.O., Abdelrahim, M.E.A., 2021. Role of repeating quarantine instructions and healthy practices on COVID-19 patients and contacted persons to raise their awareness and adherence to quarantine instructions. International Journal of Clinical Practice. 75, e14694.									x		x	
Elhadi, M., Msherghi, A., Khaled, A., Alsoufi, A., Alhadi, A., Kareem, A., Ashini, A., Alsharif, T., Alhodiri, A., Altaeb, E., Hamed, M., Itrunbah, A., Mohmmed, S., Alameen, H., Idheiraj, H., Shuwayyah, A., Alhudhairy, S., Alansari, A., Abraheem, W., Akl, H., Nagib, T., Almugaddami, A., Aljameel, B., Muamr, S., Alsuwiyah, S., Alsghair, A., Soula, E., Buzreg, A., Alagelli, F., Aldireewi, A., Bareem, A., Alshareea, E., Gemberlo, A., Zaid, A., 2022. Impact of lockdown due to the COVID-19 pandemic on mental health among the Libyan population. PLoS ONE [Electronic Resource]. 17, e0267426.								x				x
Ellwardt, L., Prag, P., 2021. Heterogeneous mental health development during the COVID-19 pandemic in the United Kingdom. Scientific Reports. 11, 15958.			x									
Enticott, J., Slifirski, W., Lavoie, K.L., Bacon, S.L., Teede, H.J., Boyle, J.A., i, C.S.T., 2021. Knowledge, Attitude, and Self-Reported Practice Towards Measures for Prevention of the Spread of COVID-19 Among Australians: A Nationwide Online Longitudinal Representative Survey. Frontiers in Public Health. 9, 630189. https://doi.org/10.3389/fpubh.2021.630189.	x										x	

Eraso, Y., Hills, S., 2021. Self-Isolation and Quarantine during the UK's First Wave of COVID-19. A Mixed-Methods Study of Non-Adherence. International Journal of Environmental Research & Public Health [Electronic Resource]. 18, 30.	x			x
Erdelyi-Hamza, B., Elek, L., Kulig, B., Kovacs, I., Fountoulakis, K.N., Smirnova, D., Gonda, X., 2021. P.0640 Factors influencing changes in anxiety during the COVID-19 related lockdown: modifiable targets for prevention and intervention. European Neuropsychopharmacology. 53(Supplement 1), S471.		x		
Erdem, Y., Ekinci, A.P., Altunay, I.K., Sivaz, O., Inal, S., Gokalp, M.O., Pehlivan, G., Ozkaya, E., 2021. The impact of COVID-19 pandemic on the management of patients with chronic urticaria: An observational two-center study from Turkey. Dermatologic Therapy. 34, e14652. https://doi.org/10.1111/dth.14652.		x		
Eslamzadeh, M., Bordbar, M.R.F., Ghalibaf, A.M., Modaresi, F., Emadzadeh, M., Farhoudi, F., 2022. The role of personality traits in following quarantine orders during the COVID-19 pandemic. International Clinical Psychopharmacology. 37, 173-178. https://doi.org/10.1097/yic.0000000000000410.	x			x
Esmaeili, M., Shahmari, M., Ghobadi, A., 2022. A qualitative study of COVID-19 home quarantine in public. Journal of Nursing and Midwifery Sciences. 9, 198-204. https://doi.org/10.4103/jnms.jnms_98_21.			x	x

Etcioglu, E., Aydin, A., Ozen, F., Kose, E., Aydin, M.R., Muratdagi, G., Kilincel, O., Kilincel, S., Ekerbicer, H.C., 2021. Anxiety and loneliness levels of quarantined citizens who brought from abroad: An example from Turkey. Erciyes Medical Journal. 43(4), 385-392.								x					x
Exploring the Mechanisms of Influence on COVID-19 Preventive Behaviors in China's Social Media Users			x										
Faizah, I., Kartini, Y., Sari, R.Y., Rohmawati, R., Afiyah, R.K., Rahman, F.S., 2021. Social support and acceptance commitment therapy on subjective well-being and mental health of covid-19 patient. Open Access Macedonian Journal of Medical Sciences. 9(G), 238-243.				x									
Farooq, A., Sheikh, T.K., Syed, F., Mustafa, T., 2021. Paediatric Contacts of Adult COVID-19 Patients: Clinical Parameters, Risk Factors, and Outcome. International Journal of Pediatrics (United Kingdom). 2021 (no pagination), 2141128.	x											x	
Flores-Torres, M. H., et al. (2021). "Prevalence and Correlates of Mental Health Outcomes During the SARS-Cov-2 Epidemic in Mexico City and Their Association With Non-adherence to Stay-At-Home Directives, June 2020." International Journal of Public Health 66.		x						x					
Foroozanfar, Z., et al. (2020). "Isolation compliance and associated factors among covid-19 patients in north-west iran: A cross-sectional study." International Journal of General Medicine 13: 1697-1703.	x											x	
Francis, J., et al. (2020). "Awareness of self-quarantine- a survey." European Journal of Molecular and Clinical Medicine 7(1): 2236-2247.			x										

Freytag, S., et al. (2021). "Gewalt und Gewaltpravention in der stationaren Altenpflege wahrend der COVID-19-Pandemie." Pflege 34(5): 241-249.		x					
Frolli, A., et al. (2020). Current Pediatric Research 24(6): 290-297.			x				
Fuchs, J. D., et al. (2021). "Assessment of a Hotel-Based COVID-19 Isolation and Quarantine Strategy for Persons Experiencing Homelessness." JAMA Network Open 4(3): e210490.	x				x		
Gainza Perez, M. A., et al. (2022). "Influence of suicidality on adult perceptions of COVID-19 risk and guideline adherence." Journal of Affective Disorders 308: 27-30.		x					
Gan, Y. Q., et al. (2022). "Immediate and delayed psychological effects of province-wide lockdown and personal quarantine during the COVID-19 outbreak in China." Psychological Medicine 52(7): 1321-1332.				x		x	
Ganslmeier, M., et al. (2022). "Compliance with the first UK covid-19 lockdown and the compounding effects of weather." Scientific Reports 12(1): 3821.		x					
Gao, Q., et al. (2022). "Quarantine and demographic characteristics as predictors of perceived stress and stress responses during the third year of COVID-19 in China." Frontiers in Psychiatry 13.				x		x	
Garcia-Sierra, R., et al. (2021). "Evaluation of a support system for health professionals confined by COVID-19." Revista de Saude Publica 55.				x			x

Garcia, F. E., et al. (2021). "Construction and validation of the Disconfinement Anxiety Scale in people exposed to the COVID-19 pandemic." Revista Iberoamericana de Diagnostico y Evaluacion Psicologica 60(3): 145-156.	x			
Gardiner, E., et al. (2022). "Perspectives of patients, family members, health professionals and the public on the impact of COVID-19 on mental health." Journal of Mental Health 31(4): 524-533.		x	x	
Gdoura, D., et al. (2021). "Psychological and behavioral impact of the COVID-19 pandemic and containment among the general tunisian population." European Psychiatry 64(Supplement 1): S278.	x			
Genc, A., et al. (2020). "COPING MECHANISMS AS MEDIATORS IN THE RELATIONSHIP BETWEEN PERCEIVED STRESS AND PRECAUTIONS DURING THE COVID-19 PANDEMIC." Primenjena Psihologija 13(4): 449-471.		x	x	
Gendler, Y. and A. Blau (2022). "COVID-19-Related Anxiety Symptoms among Quarantined Adolescents and Its Impact on Sleep Pattern Changes and Somatic Symptoms." Children-Basel 9(5).		x		x
George, G. and M. R. Thomas (2021). "Quarantined effects and strategies of college students - COVID-19." Asian Education and Development Studies 10(4): 565-573.	x			
George, G. and M. R. Thomas (2021). "Quarantined effects and strategies of college students - COVID-19." Asian Education and Development Studies 10(4): 565-573.	x			

Gok, A. (2022). "Examination of Home Quarantine Experiences of Individuals Diagnosed With COVID-19 Living in Turkey." Home Health Care Management and Practice 34(3): 229-236.	x		x		
Goldblatt, M. J., et al. (2022). "Isolation, loneliness and aloneness in the age of covid-19: Reflections on clinical experiences." British Journal of Psychotherapy: No Pagination Specified.		x			
Golemis, A., et al. (2021). "Young adults' coping strategies against loneliness during the COVID-19-related quarantine in Greece." Health Promotion International Vol 37(1), 2021, ArtID daab053 37(1).		x			
Gong, J. Y., et al. (2021). "Psychological Effects of People Isolated in Hubei Due to COVID-19 Epidemic." Frontiers in Psychiatry 12.			x		x
Goodwin, R., et al. (2020). "Quarantine, distress and interpersonal relationships during COVID-19." General Psychiatry 33(6): 1-5.		x			
Gopichandran, V. and S. Subramaniam (2021). "A qualitative inquiry into stigma among patients with Covid-19 in Chennai, India." Indian Journal of Medical Ethics VI(3): 1-21.			x		x
Gorbena, S., et al. (2022). "The effects of an intervention to improve mental health during the COVID-19 quarantine: comparison with a COVID control group, and a pre-COVID intervention group." Psychology & Health 37(2): 178-193.			x	x	
Gordeeva, T., et al. (2021). "Fear of infection and optimism predict following stayat- home recommendations during COVID-19 pandemic in russian young people." European Psychiatry 64(Supplement 1): S99.			x	x	

Govender, I. (2020). "Covid-19 with social distancing, isolation, quarantine and cooperation, collaboration, coordination of care but with disproportionate impacts." South African Family Practice 62(1): 1-2.		x			
Gradidge, P. J. L. and H. S. Kruger (2020). "Physical activity, diet and quality of life during mandatory (COVID-19) quarantine following repatriation." SAGE Open Medical Case Reports 8(no pagination).		x			
Gradinariu, G., et al. (2022). Scottish Medical Journal 67(1): 82-83.			x	x	
Gray, L., et al. (2022). "The lived experience of hotel isolation and quarantine at the Aotearoa New Zealand border for COVID-19: A qualitative descriptive study." International Journal of Disaster Risk Reduction 70.	x				
Grezo, M. and M. Adamus (2022). "Light and Dark Core of personality and the adherence to COVID-19 containment measures: The roles of motivation and trust in government." Acta Psychologica Vol 223 2022, ArtID 103483 223.	x				
Gritsenko, V., Skugarevsky, O., Konstantinov, V., Khamenka, N., Marinova, T., Reznik, A., & Isralowitz, R. (2021). COVID-19 fear, stress, anxiety, and substance use among Russian and Belarusian university students. *International Journal of Mental Health and Addiction*, *19*(6), 2362-2368.			x		x
Grondin, S., Mendoza-Duran, E., & Rioux, P. A. (2020). Pandemic, Quarantine, and Psychological Time. *Frontiers in Psychology*, *11*, Article 581036. https://doi.org/10.3389/fpsyg.2020.581036		x			

Gruhn, M., Miller, A. B., Machlin, L., Motton, S., Thinzar, C. E., & Sheridan, M. A. Child Anxiety and Depression Symptom Trajectories and Predictors over 15 Months of the Coronavirus Pandemic. *Research on Child and Adolescent Psychopathology*. https://doi.org/10.1007/s10802-022-00963-9	x		
Guermazi, A., Ben Thabet, J., Zouari, A., Aloulou, J., Hammami, R., Ben Ayed, H., Sallemi, A., Marrekchi, C., Hdiji, S., Gargouri, I., Kassis, M., Turki, M., Kammoun, S., & Masmoudi, M. L. (2021). Depression in quarantined patients during the COVID-19 pandemic. *European Psychiatry*, *64*, S280-S281. https://doi.org/10.1192/j.eurpsy.2021.753		x	x
Guillen, L. F. M., Enamorado, C. R. C., & Guillen, M. F. M. (2022). Eating Behaviors and Physical Activity Associated With Stress, Anxiety, and Depression During the COVID-19 Pandemic. *Mhsalud-Revista En Ciencias Del Movimiento Humano Y La Salud*, *19*(2). https://doi.org/10.15359/mhs.19-2.6	x		
Gulensoy, E. S., Cicek, B. G., Yuksel, A., Alhan, A., Ogan, N., & Akpinar, E. E. (2021). The effect of the novel type coronavirus infection on the mental health of the patients [Conference Abstract]. *European Respiratory Journal. Conference: International Congress of the European Respiratory Society, ERS*, *58*(SUPPL 65).	x		

Gustafsson, P. E., Nilsson, I., & San Sebastian, M. (2022). Venerable vulnerability or remarkable resilience? A prospective study of the impact of the first wave of the COVID-19 pandemic and quarantine measures on loneliness in Swedish older adults with home care [Research Support, Non-U.S. Gov't]. *BMJ Open*, *12*(5), e060209.	x
Gustavsson, J., & Beckman, L. (2020). Compliance to Recommendations and Mental Health Consequences among Elderly in Sweden during the Initial Phase of the COVID-19 Pandemic-A Cross Sectional Online Survey [Research Support, Non-U.S. Gov't]. *International Journal of Environmental Research & Public Health [Electronic Resource]*, *17*(15), 26.	x
Haas, A. N., Passos-Monteiro, E., Delabary, M. D., Moratelli, J., Schuch, F. B., Correa, C. L., Sonza, A., Guimaraes, A. C. D., & Peyre-Tartaruga, L. A. (2022). Association between mental health and physical activity levels in people with Parkinson's disease during the COVID-19 pandemic: an observational cross-sectional survey in Brazil. *Sport Sciences for Health*, *18*(3), 871-877. https://doi.org/10.1007/s11332-021-00868-y	x
Haddad, C., Zakhour, M., Kheir, M. B., Haddad, R., Al Hachach, M., Sacre, H., & Salameh, P. (2020). Association between eating behavior and quarantine/confinement stressors during the coronavirus disease 2019 outbreak. *Journal of Eating Disorders*, *8*(1), Article 40. https://doi.org/10.1186/s40337-020-00317-0	x

HaGani, N., Eilon, Y., Zeevi, S., Vaknin, L., & Baruch, H. The psychosocial impact of quarantine due to exposure to COVID-19 among healthcare workers in Israel. *Health Promotion International*. https://doi.org/10.1093/heapro/daac010		x		x
Hagezom, H. M., Gebrehiwet, A. B., Goytom, M. H., & Alemseged, E. A. (2021). Prevalence of depression and associated factors among quarantined individuals during the covid-19 pandemic in tigrai treatment and quarantine centers, Tigrai, Ethiopia, 2020: A cross-sectional study. *Infection and Drug Resistance*, *14*, 2113-2119.		x	x	
Haider, S., Smith, L., Markovic, L., Schuch, F. B., Sadarangani, K. P., Lopez Sanchez, G. F., Lopez-Bueno, R., Gil-Salmeron, A., Rieder, A., Tully, M. A., Tschiderer, L., Seekircher, L., Willeit, P., & Grabovac, I. (2021). Associations between Physical Activity, Sitting Time, and Time Spent Outdoors with Mental Health during the First COVID-19 Lock Down in Austria. *International Journal of Environmental Research & Public Health [Electronic Resource]*, *18*(17), 31.	x			
Haik, A. K., & Hussong, A. M. (2022). Impact of Covid-19 Related Stressors on Alcohol Involvement and Depressive Symptoms Post-College: Comparing Lgbtq+ and Heterosexual/Undefined-Sexuality Young Adults [Conference Abstract]. *Alcoholism: Clinical and Experimental Research*, *46(Supplement 1)*, 175A-176A.	x			

Haleemunnissa, S., Didel, S., Swami, M. K., Singh, K., & Vyas, V. (2021). Children and COVID19: Understanding impact on the growth trajectory of an evolving generation. *Children and Youth Services Review Vol 120 2021, ArtID 105754*, *120*.		x		
Haliwa, I., Lee, J., Wilson, J., & Shook, N. J. (2020). Mindfulness and engagement in COVID-19 preventive behavior. *Preventive Medicine Reports*, *20 (no pagination)*,			x	x
Hamdan, A., Ghanim, M., & Mosleh, R. (2021). COVID-19 confinement and related well being measurement using the EQ-5D questionnaire: A survey among the Palestinian population. *International Journal of Clinical Practice*, *75*(10), e14621.	x			
Han, L. Y., Anuar, N. S. B., Sivapatham, L., & Koong, C. L. K. (2022). Pioneering home quarantine for obstetric Covid-19 patients in Malaysia using a mobile application-based home assessment tool [Conference Abstract]. *Medical Journal of Malaysia*, *77(Supplement 2)*, 23.			x	x
Han, T., Ma, W., Gong, H., Hu, Y., Zhang, Y., Zhang, C., Yao, Z., Fan, Y., Zheng, Y., & Wang, C. (2021). Investigation and analysis of negative emotion among university students during home quarantine of COVID-19. [Chinese]. *Journal of Xi'an Jiaotong University (Medical Sciences)*, *42(1)*, 132-136.	x			

Handberg, C., Werlauff, U., Hojberg, A. L., & Knudsen, L. F. (2021). Impact of the COVID-19 pandemic on biopsychosocial health and quality of life among Danish children and adults with neuromuscular diseases (NMD)-Patient reported outcomes from a national survey. *PLoS ONE [Electronic Resource]*, *16*(6), e0253715.	x
Hann, A., Lembach, H., McKay, S., Sneiders, D., Okoth, K., Anderton, J., Nirantharakumar, K., Magill, L., Torlinska, B., Armstrong, M., Mascaro, J., Inston, N., Pinkney, T., Ranasinghe, A., Borrows, R., Ferguson, J., Isaac, J., Calvert, M., Perera, T., & Hartog, H. (2021). Prospective evaluation of health-related quality of life, uncertainty and coping strategies in organ transplant recipients during the covid pandemic [Conference Abstract]. *Transplant International*, *34(SUPPL 1)*, 210-211.	x
Harris, R. (2021). Experiences with testing, self-isolation and vaccination in north east england during the covid pandemic. *Vaccines*, *9(7) (no pagination)*, Article 759.	x
Hartley, S., Colas des Francs, C., Aussert, F., Martinot, C., Dagneaux, S., Londe, V., Waldron, L., & Royant-Parola, S. (2020). The effects of quarantine for SARS-CoV-2 on sleep: An online survey. *L'Encephale: Revue de psychiatrie clinique biologique et therapeutique*, *46*(3, Suppl), S53-S59.	x
Hasina, S. N., Sulistyorini, S., Nisa, F., & Afiyah, R. (2021). Music therapy for anxiety level and quality of life of patients infected by covid-19. *Open Access Macedonian Journal of Medical Sciences, Part G*. *9*, 251-255.	x

Hassan-Abbas, N. M. Daily stress, family functioning and mental health among Palestinian couples in Israel during COVID-19: A moderated mediation model. *Journal of Social and Personal Relationships*. https://doi.org/10.1177/02654075221130785		x		
Hassan, S. M., Ring, A., Tahir, N., & Gabbay, M. (2021). The impact of COVID-19 social distancing and isolation recommendations for Muslim communities in North West England [Research Support, Non-U.S. Gov't]. *BMC Public Health*, *21*(1), 812.		x		
Havlioglu, S., Kahraman, S., Kizir, Y., & Acar, U. (2022). Online identification of obsessive-compulsive symptoms and relevant factors in patients with Covid-19 in Turkey during quarantine. *European Journal of Psychology Open*, No Pagination Specified.	x		x	
Hayat, A. A., Meny, A., Hamdan, Q. U., Sami, W., & Albadrani, G. (2022). Seeing the unforeseen: an insight into kingdom-wide psychological impact of COVID-19 pandemic. *European Review for Medical and Pharmacological Sciences*, *26(16)*, 5956-5962		x		
Hayden, M. E., Rozycki, D., Tanabe, K. O., Pattie, M., Casteen, L., Davis, S., & Holstege, C. P. (2021). COVID-19 Isolation and Quarantine Experience for Residential Students at a Large Four-Year Public University. *American Journal of Public Health*, *111*(10), 1772-1775. https://doi.org/10.2105/ajph.2021.306424		x		
Hazan, H., & Chan, C. S. (2022). Indirect contact with nature, lifestyle, and mental health outcomes during mandatory hotel quarantine in Hong Kong. *Journal of Mental Health*.			x	x

Hermosa-Bosano, C., Paz, C., Hidalgo-Andrade, P., Garcia-Manglano, J., Chalezquer, C. S., Lopez-Madrigal, C., & Serrano, C. (2021). Depression, anxiety and stress symptoms experienced by the ecuadorian general population during the pandemic for covid-19. [Spanish] [Sintomas de depresion, ansiedad y estres en la poblacion general ecuatoriana durante la pandemia por COVID-19.]. *Revista Ecuatoriana de Neurologia*, *30(2)*, 40-47.	x			
Hernandez, L. D., Giezendanner, S., Fischer, R., & Zeller, A. (2021). The effect of COVID-19 on mental well-being in Switzerland: a cross-sectional survey of the adult Swiss general population. *Bmc Family Practice*, *22*(1), Article 181. https://doi.org/10.1186/s12875-021-01532-7	x			
Holladay, K., Lardier, D., Amorim, F. T., Zuhl, M., & Coakley, K. E. (2022). Practicing COVID-19 Public Health Measures Is Associated With Anxiety and Depression in Undergraduate University Students. *Frontiers in Public Health*, *10*, 941730.		x		
Holubnycha, L., Khodakovska, O., Besarab, T., Dolgusheva, O., & Malieieva, T. (2021). Psychological impact of online learning during covid-19 pandemic on students' mental health as life quality basis. *Amazonia Investiga*, *10*(47), 70-80. https://doi.org/10.34069/ai/2021.47.11.8			x	x

Holzinger, B., Nierwetberg, F., Chung, F., Bolstad, C. J., Bjorvatn, B., Chan, N. Y., Dauvilliers, Y., Espie, C. A., Han, F., Inoue, Y., Leger, D., Macedo, T., Matsui, K., Merikanto, I., Morin, C. M., Mota-Rolim, S. A., Partinen, M., Plazzi, G., Penzel, T., . . . De Gennaro, L. (2022). Has the COVID-19 Pandemic Traumatized Us Collectively? The Impact of the COVID-19 Pandemic on Mental Health and Sleep Factors via Traumatization: A Multinational Survey. *Nature and Science of Sleep*, *14*, 1469-1483. https://doi.org/10.2147/nss.S368147		x	
Hong, R. H., Brar, R., & Fairbairn, N. (2022). Supporting Self-isolation for COVID-19 With "Risk Mitigation" Prescribing and Housing Supports for People Who Use Drugs: A Case Report [Case Reports]. *Journal of Addiction Medicine*, *16*(5), 592-594.		x	
Hood, J. E., Kubiak, R. W., Avoundjian, T., Kern, E., Fagalde, M., Collins, H. N., Meacham, E., Baldwin, M., Lechtenberg, R. J., Bennett, A., Thibault, C. S., Stewart, S., Duchin, J. S., & Golden, M. R. (2022). A Multifaceted Evaluation of a COVID-19 Contact Tracing Program in King County, Washington [Research Support, Non-U.S. Gov't]. *Journal of Public Health Management & Practice*, *28*(4), 334-343.	x		x
Horta, R. L., Camargo, E. G., Barbosa, M. L. L., Lantin, P. J. S., Sette, T. G., Lucini, T. C. G., Silveira, A. F., Zanini, L., & Lutzky, B. A. (2021). Front line staff stress and mental health during COVID-19 pandemic in a general hospital. *Jornal Brasileiro de Psiquiatria*, *70*(1), 30-38.		x	

Hou, J., Yu, Q. Y., & Lan, X. Y. (2021). COVID-19 Infection Risk and Depressive Symptoms Among Young Adults During Quarantine: The Moderating Role of Grit and Social Support. *Frontiers in Psychology*, *11*, Article 577942. https://doi.org/10.3389/fpsyg.2020.577942	x		
Hu, W., Nakamura, C., Abdallah, M., Chavarria, M., Hite, A., Lim, Y., & Suddath, R. (2021). 37.7 The Impact of Online Learning and the COVID-19 Pandemic on Child and Adolescent Psychiatric Patients [Conference Abstract]. *Journal of the American Academy of Child and Adolescent Psychiatry*, *60(10 Supplement)*, S224.	x		
Huang, S. F., Xiao, Y. R., Yan, L., Deng, J., He, M., Lu, J., & Ke, S. (2020). Implications for Online Management: Two Cases with COVID-19. *Telemedicine and E-Health*, *26*(4), 487-494. https://doi.org/10.1089/tmj.2020.0066		x	x
Huerta, C. M., & Cafagna, G. (2021). Snapshot of the Use of Urban Green Spaces in Mexico City during the COVID-19 Pandemic: A Qualitative Study. *International Journal of Environmental Research and Public Health*, *18*(8), Article 4304. https://doi.org/10.3390/ijerph18084304	x		
Huth, T., Schmidt, C., Smith, D. G., Lin, W. D., & Wang, A. (2022). Spiritual Health Predicts Improved PHQ-9 During COVID Isolation in Extended Care Facility Patients [Letter]. *Journal of the American Medical Directors Association*, *23(8)*, 1284-1285.	x		

Huy, N. T., Duc, N. T. M., Alhady, S. T. M., Mai, L. N., Hassan, A. K., Giang, T. V., Truong, L. V., Ravikulan, R., Raut, A., Dayyab, F. M., Durme, S. P., Trang, V. T. T., Loc, L. Q., Thach, P. N., & Collaborative, T. M. G. H. G. C. (2021). Perceived Stress of Quarantine and Isolation During COVID-19 Pandemic: A Global Survey. *Frontiers in Psychiatry*, *12*, Article 656664. https://doi.org/10.3389/fpsyt.2021.656664		x	
Hyland, P., Shevlin, M., McBride, O., Murphy, J., Karatzias, T., Bentall, R. P., Martinez, A., & Vallieres, F. (2020). Anxiety and depression in the Republic of Ireland during the COVID-19 pandemic. *Acta Psychiatrica Scandinavica*, *142*(3), 249-256.	x		
Hyun, J., Kim, S., Kim, H., Choi, Y. J., Choi, Y. K., Lee, Y. R., Paik, J. W., Lee, J. S., Kim, K., Jun, J. Y., Lee, S. H., & Sohn, S. (2021). COVID-19 and risk factors of anxiety and depression in South Korea. *Psychiatry Investigation*, *18(9)*, 801-808.			x
Hyun, W. I., Son, Y. H., & Jung, S. O. (2022). Infection preventive behaviors and its association with perceived threat and perceived social factors during the COVID-19 pandemic in South Korea: 2020 community health survey. *BMC Public Health*, *22*(1), 1381.	x		

Irigoyen-Camacho, M. E., Velazquez-Alva, M. C., Zepeda-Zepeda, M. A., Cabrer-Rosales, M. F., Lazarevich, I., & Castano-Seiquer, A. (2020). Effect of Income Level and Perception of Susceptibility and Severity of COVID-19 on Stay-at-Home Preventive Behavior in a Group of Older Adults in Mexico City. *International Journal of Environmental Research and Public Health*, *17*(20), Article 7418. https://doi.org/10.3390/ijerph17207418		x	
Isherwood, K. R., Kyle, R. G., Gray, B. J., & Davies, A. R. Challenges to self-isolation among contacts of cases of COVID-19: a national telephone survey in Wales. *Journal of Public Health*. https://doi.org/10.1093/pubmed/fdac002	x		x
Islam, M., Sujan, M., Tasnim, R., Mohona, R. A., Ferdous, M., Kamruzzaman, S., Toma, T. Y., Sakib, M., Pinky, K. N., Islam, M., Siddique, M., Anter, F. S., Hossain, A., Hossen, I., Sikder, M., & Pontes, H. M. (2021). Problematic smartphone and social media use among Bangladeshi college and university students amid COVID-19: The role of psychological well-being and pandemic related factors. *Frontiers in Psychiatry Vol 12 2021, ArtID 647386*, *12*.		x	
Ismael, F., Bizario, J. C. S., Battagin, T., Zaramella, B., Leal, F. E., Torales, J., Ventriglio, A., Marziali, M. E., Martins, S. S., & Castaldelli-Maia, J. M. (2021). Post-infection depressive, anxiety and post-traumatic stress symptoms: A prospective cohort study in patients with mild COVID-19 [Research Support, N.I.H., Extramural Research Support, Non-U.S. Gov't]. *Progress in Neuro-Psychopharmacology & Biological Psychiatry*, *111*, 110341.		x	

Ismael, F., Zaramella, B., Battagin, T., Bizario, J. C. S., Gallego, J., Villela, V., de Queiroz, L. B., Leal, F. E., Torales, J., Ventriglio, A., Marziali, M. E., Goncalves, P. D., Martins, S. S., & Castaldelli-Maia, J. M. (2021). Substance Use in Mild-COVID-19 Patients: A Retrospective Study [Research Support, N.I.H., Extramural Research Support, Non-U.S. Gov't]. *Frontiers in Public Health*, *9*, 634396.	x			
Ivbijaro, G., Brooks, C., Kolkiewicz, L., Sunkel, C., & Long, A. (2020). Psychological impact and psychosocial consequences of the COVID 19 pandemicResilience, mental well-being, and the coronavirus pandemic [Review]. *Indian Journal of Psychiatry*, *62(9 Supplement 3)*, S395-S403.	x			
Izurieta-Brito, D., Poveda-Rios, S., Naranjo-Hidalgo, T., & Moreno-Montero, E. (2022). Generalized anxiety disorder and academic stress in university students during the COVID-19 pandemic. [Spanish] [Trastorno de ansiedad generalizada y estres academico en estudiantes universitarios ecuatorianos durante la pandemia COVID-19.]. *Revista de Neuro-Psiquiatria*, *85(2)*, 86-94.			x	x
Jabbar, J., Dharmarajan, S., Ramachandran, A. P., & Jasseer, A. (2021). Coping with COVID-19: A Qualitative Analysis. *Asr Chiang Mai University Journal of Social Sciences and Humanities*, *8*(2), Article e2021007. https://doi.org/10.12982/cmujasr.2021.007	x			
Jafri, M. R., Zaheer, A., Fatima, S., Saleem, T., & Sohail, A. (2022). Mental health status of COVID-19 survivors: a cross sectional study. *Virology Journal*, *19*(1), 3.		x		

Jagadeesan, T., Archana, R., Kannan, R., Jain, T., Allu, A. R., Selvi, G. T., Maveeran, M., & Kuppusamy, M. (2022). Effect of Bhramari Pranayama intervention on stress, anxiety, depression and sleep quality among COVID 19 patients in home isolation. *Journal of Ayurveda and Integrative Medicine*, *13*(3), Article 100596. https://doi.org/10.1016/j.jaim.2022.100596	x		x
Jahan, I., Hosen, I., al Mamun, F., Kaggwa, M. M., Griffiths, M. D., & Mamun, M. A. (2021). How Has the COVID-19 Pandemic Impacted Internet Use Behaviors and Facilitated Problematic Internet Use? A Bangladeshi Study. *Psychology Research and Behavior Management*, *14*, 1127-1138. https://doi.org/10.2147/prbm.S323570		x	
James, J. E., Riddle, L., & Perez-Aguilar, G. (2022). "Prison life is very hard and it's made harder if you're isolated": COVID-19 risk mitigation strategies and the mental health of incarcerated women in California [Research Support, N.I.H., Extramural Research Support, Non-U.S. Gov't]. *International journal of prison health.. ahead of print*, *11*, 21.		x	
Jane-Llopis, E., Anderson, P., Segura, L., Zabaleta, E., Munoz, R., Ruiz, G., Rehm, J., Cabezas, C., & Colom, J. (2021). Mental ill-health during COVID-19 confinement. *BMC Psychiatry*, *21*(1), 194.		x	
Jang, H. Y., Ko, Y., & Han, S. Y. (2022). Factors Associated With Depressive Symptoms in Individuals Who Have Experienced COVID-19 Self-Quarantine. *Frontiers in Public Health*, *10*, 810475.	x		x

Jassim, G., Jameel, M., Brennan, E., Yusuf, M., Hasan, N., & Alwatani, Y. (2021). Psychological impact of covid-19, isolation, and quarantine: A cross-sectional study. *Neuropsychiatric Disease and Treatment*, *17*, 1413-1421.			x	x
Jauffret-Roustide, M., Barratt, M., De Dinechin, S., Davies, E., Gilchrist, G., Hughes, C., Maier, L., Ferris, J., & Winstock, A. (2020). Consumption of alcohol and other psychoactive drugs during the covid-19 pandemic in the global drug survey: A French perspective. [French] [Consommation d'alcool et d'autres produits psychoactifs pendant la pandemie de Covid- 19 dans la Global Drug Survey: une perspective francaise.] [Review].		x		
Jawa, T. M. (2022). Logistic regression analysis for studying the impact of home quarantine on psychological health during COVID-19 in Saudi Arabia. *Alexandria Engineering Journal*, *61*(10), 7995-8005. https://doi.org/10.1016/j.aej.2022.01.047		x		
Jeong, S. J., Chung, W. S., Sohn, Y., Hyun, J. H., Baek, Y. J., Cho, Y., Kim, J. H., Ahn, J. Y., Choi, J. Y., & Yeom, J. S. (2020). Clinical characteristics and online mental health care of asymptomatic or mildly symptomatic patients with coronavirus disease 2019. *PLoS ONE [Electronic Resource]*, *15*(11), e0242130.			x	x
Jesmi, A. A., Mohammadzade-Tabrizi, Z., Rad, M., Hosseinzadeh-Younesi, E., & Pourhabib, A. (2021). Lived experiences of patients with COVID-19 infection: a phenomenology study. *Medicinski Glasnik Ljekarske Komore Zenickodobojskog Kantona*, *18*(1), 18-26.	x		x	

Jilowa, C. S., Meena, P. S., Jain, M., Prakash, P., & Tak, P. (2022). Mental Health Status among the Quarantined Population during COVID-19 Pandemic: A Cross-sectional Study from Western Rajasthan. *Journal of Clinical and Diagnostic Research*, *16(5)*, VC01-VC04.				x	x
Jimenez, O., Sanchez-Sanchez, L. C., & Garcia-Montes, J. M. (2020). Psychological Impact of COVID-19 Confinement and Its Relationship with Meditation. *International Journal of Environmental Research and Public Health*, *17*(18), Article 6642. https://doi.org/10.3390/ijerph17186642		x			
Joisten. Christine, J., Barbara, G., Alisa, F., Sven, F., Andreas, G., Lisa, K., Johannes, N., Marc, T., Stefanie, W., Gerhard, A. W., & Annelene, K. (2022). Adherence, psychosocial Consequences, Coping Strategies and Lifestyle of Cologne COVID-19 Patients and their Close Contacts in the Context of an officially ordered Quarantine - first Results of the CoCo-Fakt surveillance Study, Cologne. *Gesundheitswesen*, *84*(4), 349-349. https://doi.org/10.1055/s-0042-1745439	x			x	
Jones, C., Kaizi-Lutu, M., Cordoza, M., Mange, A., & Dinges, D. (2021). Changes in sleep amount and sleep quality due to the COVID-19 pandemic confinement associate with ratings of health and stress [Conference Abstract]. *Sleep*, *44(SUPPL 2)*,			x		
Ju, Y. J., Kim, W., & Lee, S. Y. (2022). Perceived social support and depressive symptoms during the COVID-19 pandemic: A nationally-representative study. *The International journal of social psychiatry*, 207640211066737.		x			

Ju, Y., Chen, W., Liu, J., Yang, A., Shu, K., Zhou, Y., Wang, M., Huang, M., Liao, M., Liu, J., Liu, B., & Zhang, Y. (2021). Effects of centralized isolation vs. home isolation on psychological distress in patients with COVID-19 [Observational Study]. *Journal of Psychosomatic Research*, *143*, 110365.	x		x
Jupille, J., Deloffre, S., Hulin, P., Harscoet, Y. A., Vincent, M., Leguay, D., & Chirio-Espitalier, M. (2021). Crossed views on confinement: people living with mental disorders, families, and caregivers. *Sante Publique*, *33*(6), 875-883. https://doi.org/10.3917/spub.216.0875		x	
Kaba, D., Hasanli, J., Efe, A., Yavuz-Colak, M., & Akin-Sari, B. Predictors of burnout and distress in parents of children with autism spectrum disorder during COVID-19 home confinement. *Childrens Health Care*. https://doi.org/10.1080/02739615.2022.2119974		x	
Kachanoff, F. J., Bigman, Y. E., Kapsaskis, K., & Gray, K. (2021). Measuring Realistic and Symbolic Threats of COVID-19 and Their Unique Impacts on Well-Being and Adherence to Public Health Behaviors. *Social Psychological and Personality Science*, *12*(5), 603-616, Article 1948550620931634. https://doi.org/10.1177/1948550620931634		x	
Kahn, M., Barnett, N., Glazer, A., & Gradisar, M. (2021). Infant sleep during COVID-19: Longitudinal analysis of infants of US mothers in home confinement versus working as usual [Research Support, Non-U.S. Gov't]. *Sleep Health*, *7*(1), 19-23.		x	

Kaim, A., Siman-Tov, M., Jaffe, E., & Adini, B. (2021). Factors that enhance or impede compliance of the public with governmental regulation of lockdown during COVID-19 in Israel. *International Journal of Disaster Risk Reduction*, *66*, Article 102596. https://doi.org/10.1016/j.ijdrr.2021.102596	x		
Kainja, J., Ndasauka, Y., McHenga, M., Kondowe, F., M'Manga, C., Maliwichi, L., & Nyamali, S. (2022). Umunthu, Covid-19 and mental health in Malawi. *Heliyon*, *8*(11), Article e11316. https://doi.org/10.1016/j.heliyon.2022.e11316	x		
Kamin, T., Perger, N., Debevec, L., & Tivadar, B. (2021). Alone in a Time of Pandemic: Solo-Living Women Coping With Physical Isolation. *Qualitative Health Research*, *31*(2), 203-217. https://doi.org/10.1177/1049732320971603	x		
Kang, E., Lee, S. Y., Kim, M. S., Jung, H., Kim, K. H., Kim, K. N., Park, H. Y., Lee, Y. J., Cho, B., & Sohn, J. H. (2021). The Psychological Burden of COVID-19 Stigma: Evaluation of the Mental Health of Isolated Mild Condition COVID-19 Patients [Observational Study]. *Journal of Korean Medical Science*, *36*(3), e33.		x	x
Kannampallil, T. G., Goss, C. W., Evanoff, B. A., Strickland, J. R., McAlister, R. P., & Duncan, J. (2020). Exposure to COVID-19 patients increases physician trainee stress and burnout. *PLoS ONE [Electronic Resource]*, *15*(8), e0237301.	x		
Kaplan, V. (2021). The Burnout and Loneliness Levels of Housewives in Home-Quarantine during Covid-19 Pandemic. *Cyprus Turkish Journal of Psychiatry and Psychology*, *3*(2), 115-122. https://doi.org/10.35365/ctjpp.21.2.13	x		

Karim, M. A., Ouanes, S., Reagu, S. M., & Alabdulla, M. (2021). Network analysis of anxiety and depressive symptoms among quarantined individuals: cross-sectional study. *Bjpsych Open*, *7*(6), Article e222. https://doi.org/10.1192/bjo.2021.1060				x	x
Karlidag, G. E., Kantarcioglu, A., Toraman, Z. A., Balci, H. N., Gulmez, E., & Atmaca, M. (2021). Effect of Infection on Mental Health in COVID-19 Positive Cases and its Relationship with Clinical Variables. *Psychiatry and Clinical Psychopharmacology*, *31(1)*, 83-89.		x			
Kaur, R., Kant, S., Bairwa, M., Kumar, A., Dhakad, S., Dwarakanathan, V., Ahmad, A., Pandey, P., Kapil, A., Lodha, R., & Wig, N. (2021). Risk Stratification as a Tool to Rationalize Quarantine of Health Care Workers Exposed to COVID-19 Cases: Evidence From a Tertiary Health Care Center in India. *Asia-Pacific Journal of Public Health*, *33*(1), 134-137, Article 1010539520977310. https://doi.org/10.1177/1010539520977310	x				
Kaygisiz, B. B., Topcu, Z. G., Meric, A., Gozgen, H., & Coban, F. (2020). Determination of exercise habits, physical activity level and anxiety level of postmenopausal women during COVID-19 pandemic. *Health Care for Women International*, *41*(11-12), 1240-1254. https://doi.org/10.1080/07399332.2020.1842878			x		
Kent, J. N., & Kilby, C. J. Predictors of psychological distress during self-isolation. *Psychology and Psychotherapy-Theory Research and Practice*. https://doi.org/10.1111/papt.12432	x				

Kerr, C. C., Mistry, D., Stuart, R. M., Rosenfeld, K., Hart, G. R., Nunez, R. C., Cohen, J. A., Selvaraj, P., Abeysuriya, R. G., Jastrzebski, M., George, L., Hagedorn, B., Panovska-Griffiths, J., Fagalde, M., Duchin, J., Famulare, M., & Klein, D. J. (2021). Controlling COVID-19 via test-trace-quarantine [Research Support, Non-U.S. Gov't]. *Nature communications*, *12*(1), 2993.				x	x
Kesgin, M. T., Tok, H. H., Uzun, L. N., & Pehlivan, S. (2022). Comparison of anxiety levels of hospitalized COVID-19 patients, individuals under quarantine, and individuals in society. *Perspectives in Psychiatric Care*, *58*(1), 149-158. https://doi.org/10.1111/ppc.12857	x				
Kettani, Z. (2020). Somatization Scale: Differentiating between the symptoms of Covid-19 and somatization in young adults and in the elderly. *NPG Neurologie - Psychiatrie - Geriatrie*, *20*(120), 339-345.			x		x
Keyworth, C., Epton, T., Byrne-Davis, L., Leather, J. Z., & Armitage, C. J. (2021). What challenges do UK adults face when adhering to COVID-19-related instructions? Cross-sectional survey in a representative sample [Research Support, Non-U.S. Gov't]. *Preventive Medicine*, *147*, 106458.	x				
Khadhar, M., Nasri, I., Lazzez, R., Bouassida, S., Sallemi, N., Agrebi, S., Ben Hamida, F., Goucha, R., & Ben Addallah, T. (2021). Pos-530 Anxiety and Depression in Hemodialysis Patients during Covid-19 [Conference Abstract]. *Kidney International Reports*, *6(4 Supplement)*, S231.		x			

Khaledi, H. J., Faizi, M., & Khakzand, M. (2022). The effects of personal green spaces on human's mental health and anxiety symptoms during COVID-19: The case of apartment residents in Tehran. *Frontiers in Built Environment*, *8*, Article 981582. https://doi.org/10.3389/fbuil.2022.981582	x		
Khan, A. H., Sultana, M. S., Hossain, S., Hasan, M. T., Ahmed, H. U., & Sikder, M. T. (2020). The impact of COVID-19 pandemic on mental health & wellbeing among home-quarantined Bangladeshi students: A cross-sectional pilot study. *Journal of Affective Disorders*, *277*, 121-128.	x		
Khan, Y. S., Khan, A. W., Ahmed, I. A. N., Hammoudeh, S., Salim, H., AbuKhattab, M., Al-Maslamani, M., Zainel, A., Salameh, S. N., & Alabdulla, M. (2021). Prevalence of elevated anxiety symptoms among children in quarantine with COVID-19 infection in the State of Qatar: A cross-sectional study. *Scandinavian Journal of Child and Adolescent Psychiatry and Psychology*, *9*, 187-195. https://doi.org/10.21307/sjcapp-2021-021		x	x
Khodami, M. A., Seif, M. H., Koochakzadeh, R. S., Fathi, R., & Kaur, H. (2022). Perceived stress, emotion regulation and quality of life during the Covid-19 outbreak: A multi-cultural online survey. *Annales Medico-Psychologiques*, *180*(6), 514-518. https://doi.org/10.1016/j.amp.2021.02.005	x		

Kilgore, L. J., Murphy, B. L., Postlewait, L. M., Liang, D. H., Bedrosian, I., Lucci, A., Kuerer, H. M., Hunt, K. K., & Teshome, M. (2021). Impact of the early COVID-19 pandemic on Breast Surgical Oncology fellow education. *Journal of Surgical Oncology*, *124*(7), 989-994.	x	
Kilincel, S., Kilincel, O., Muratdagi, G., Aydin, A., & Usta, M. B. (2021). Factors affecting the anxiety levels of adolescents in home-quarantine during COVID-19 pandemic in Turkey. *Asia-Pacific psychiatry : Official Journal of the Pacific Rim College of Psychiatrists*, *13*(2), e12406.	x	
Kim, H., Park, K. J., Shin, Y. W., Lee, J. S., Chung, S., Lee, T., Kim, M. J., Jung, J., Lee, J., Yum, M. S., Lee, B. H., Koh, K. N., Ko, T. S., Lim, E., Lee, J. S., Lee, J. Y., Choi, J. Y., Han, H. M., Shin, W. A., . . . Kim, H. W. (2020). Psychological Impact of Quarantine on Caregivers at a Children's Hospital for Contact with Case of COVID-19. *Journal of Korean Medical Science*, *35*(28), e255.		x
Kim, J. W., Kang, H. J., Jhon, M., Ryu, S., Lee, J. Y., Kang, S. J., Jung, S. I., Shin, I. S., Kim, S. W., Stewart, R., & Kim, J. M. (2021). Associations Between COVID-19 Symptoms and Psychological Distress. *Frontiers in Psychiatry*, *12 (no pagination)*,		x
Kim, J., Kim, Y., & Ha, J. (2021). Changes in Daily Life during the COVID-19 Pandemic among South Korean Older Adults with Chronic Diseases: A Qualitative Study. *International Journal of Environmental Research & Public Health [Electronic Resource]*, *18*(13), 24.	x	

Kim, S. B., Lee, J. H., An, Y. W., Lee, H. J., & Yoon, S. Y. (2021). The psychological impact of COVID-19 pandemic in quarantine population [Conference Abstract]. *Asia Pacific Psychiatry. Conference: 19th International Congress of the Pacific Rim College of Psychiatrists. Seoul South Korea*, *13*(SUPPL 1).	x												
Kim, S. C., Quiban, C., Sloan, C., & Montejano, A. (2021). Predictors of poor mental health among nurses during COVID-19 pandemic. *Nursing Open*, *8*(2), 900-907.						x							x
Kim, S. H., & Han, M. A. (2022). Depression and Related Factors in Korean Adults During the Coronavirus Disease 2019 Outbreak. *Psychiatry Investigation*, *19(11)*, 965-972.		x											
Kim, Y., Kwon, H. Y., Lee, S., & Kim, C. B. (2021). Depression During COVID-19 Quarantine in South Korea: A Propensity Score-Matched Analysis. *Frontiers in Public Health*, *9*, 743625.						x					x		
Kiseleva, M. (2021). Personal traits and coping strategies in compliance with COVID-19 preventive measures [Conference Abstract]. *European Psychiatry*, *64(Supplement 1)*, S300-S301.				x									
Klooster, J., van Gend, J. E., Schreijer, M. A., de Witte, E. R., & Van Gemert-Pijnen, L. (2021, Dec 20-22). The Value of eCoaching in the COVID-19 Pandemic to Promote Adherence to Self-isolation and Quarantine.*Lecture Notes in Computer Science* [Intelligent human computer interaction, ihci 2021]. 13th International Conference on Intelligent Human Computer Interaction (IHCI), Kent State Univ, Design Innovat Hub, Kent, OH.						x			x				

Koc, A., Tok, H. H., Uzun, L. N., & Ensari, H. (2021). Depression, anxiety and state guilt in individuals under quarantine in an institution due to COVID-19 and the related factors. *Noropsikiyatri Arsivi*, *58*(2), 146-153.			x	x
Kolcu, G., & Baser Kolcu, M. I. (2021). Psychological Effects of COVID-19 in Medical Students. *Psychiatria Danubina*, *33*(Suppl 13), 387-391.		x		
Kolodziejczyk, A., Misiak, B., Szczesniak, D., Maciaszek, J., Ciulkowicz, M., Luc, D., Wieczorek, T., Fila-Witecka, K., Chladzinska-Kiejna, S., & Rymaszewska, J. (2021). Coping Styles, Mental Health, and the COVID-19 Quarantine: A Nationwide Survey in Poland. *Frontiers in Psychiatry*, *12 (no pagination)*, Article 625355.		x		
Kontsevaya, A. V., Mukaneeva, D. K., Myrzamatova, A. O., Okely, A. D., & Drapkina, O. M. (2021). Changes in physical activity and sleep habits among adults in Russian Federation during COVID-19: a cross-sectional study. *BMC Public Health*, *21*(1), 893.		x		
Kopilas, V., Hasratian, A. M., Martinelli, L., Ivkic, G., Brajkovic, L., & Gajovic, S. (2021). Self-Perceived Mental Health Status, Digital Activity, and Physical Distancing in the Context of Lockdown Versus Not-in-Lockdown Measures in Italy and Croatia: Cross-Sectional Study in the Early Ascending Phase of the COVID-19 Pandemic in March 2020. *Frontiers in Psychology*, *12*, Article 621633. https://doi.org/10.3389/fpsyg.2021.621633	x		x	

Kortianou, E., Mavronasou, A., Tsimouris, D., Kazatzis, N., Apostolara, Z. E., Lekkas, S., Isakoglou, M., Tsikrika, S., Liontos, A., Christaki, M., Milionis, C., & Kalomenidis, I. (2021). Monitoring physical and psychological status in home-quarantined patients with COVID-19 after hospitalization [Conference Abstract]. *European Respiratory Journal*. *Conference: International Congress of the European Respiratory Society, ERS*, *58*(SUPPL 65).			x	x
Kostenko, A. M., Svitailo, N. D., Nazarov, M. S., Kurochkina, V. S., & Smiianov, Y. V. (2021). Strengthening Societal Resilience during Covid-19 Pandemic. *Wiadomosci Lekarskie*, *74*(5), 1137-1141.	x			
Kotera, Y., Green, P., Rhodes, C., Williams, A., Chircop, J., Spink, R., Rawson, R., & Okere, U. (2020). Dealing With Isolation Using Online Morning Huddles for University Lecturers During Physical Distancing by COVID-19: Field Notes. *International Review of Research in Open and Distributed Learning*, *21*(4), 238-244. <Go to ISI>://WOS:000595826000013		x		
Kothari, R., Sparrow, J., Henshall, J., Buchan, D., Kemp, J., Owen, A., Blakeman, I., & Sarkissian, N. (2022). Locked Up and Locked Down: How the Covid-19 Pandemic has Impacted the Mental Health of Male Prisoners and Support Staff. *Journal of Mens Health*, *18*(6), Article 141. https://doi.org/10.31083/j.jomh1806141	x			

Kowalczuk, I., & Gebski, J. (2021). Impact of Fear of Contracting COVID-19 and Complying with the Rules of Isolation on Nutritional Behaviors of Polish Adults. *International Journal of Environmental Research & Public Health [Electronic Resource]*, *18*(4), 09.		x			
Kowalski, E., Schneider, A., Zipfel, S., Stengel, A., & Graf, J. (2021). SARS-CoV-2 Positive and Isolated at Home: Stress and Coping Depending on Psychological Burden. *Frontiers in Psychiatry*, *12 (no pagination)*, Article 748244.	x			x	
Kowalski, J., Marchlewska, M., Molenda, Z., Gorska, P., & Gaweda, L. (2020). Adherence to safety and self-isolation guidelines, conspiracy and paranoia-like beliefs during COVID-19 pandemic in Poland - associations and moderators [Research Support, Non-U.S. Gov't]. *Psychiatry Research*, *294*, 113540.		x			
Kozhina, A. A., & Vinokurov, L. V. (2020). Work alienation during COVID-19: Main factors and conditions (an example of university professors). *Psychology in Russia: State of the Art*, *13*(4), 106-118.		x			
Kumar, K., Mehra, A., Jha, S., Sharma, R., Saini, L., Sahoo, S., Laxmi, P. V. M., Bhalla, A., & Grover, S. (2021). Psychological Morbidity among People in Quarantine. *Journal of Neurosciences in Rural Practice*, *12*(01), 67-70. https://doi.org/10.1055/s-0040-1718855				x	x
Kumchenko, S., Rasskazova, E., & Tkhostov, A. (2021). Beliefs about coronavirus: Relationship with magical thinking and adherence to self-isolation regimen [Conference Abstract]. *European Psychiatry*, *64(Supplement 1)*, S281.			x		

Kurt, D., & Dalkiran, S. S. (2022). Determining the Compliance of Intern Nursing Students with Isolation Precautions in the COVID-19 Pandemic Period. *Bezmialem Science*, *10*(4), 493-499. https://doi.org/10.14235/bas.galenos.2021.5550	x		
Kwon, H. Y., Kim, Y., & Lee, S. Y. (2022). What Matters for Depression and Anxiety During the COVID-19 Quarantine?: Results of an Online Cross-Sectional Survey in Seoul, South Korea. *Frontiers in Psychiatry*, *13 (no pagination)*, Article 706436.		x	x
Laar, R. A., Ashraf, M. A., Ning, J., Ji, P. G., Fang, P., Yu, T. R., & Khan, M. N. (2021). Performance, Health, and Psychological Challenges Faced by Students of Physical Education in Online Learning during COVID-19 Epidemic: A Qualitative Study in China. *Healthcare*, *9*(8), Article 1030. https://doi.org/10.3390/healthcare9081030	x		
Lacomba-Trejo, L., Calderon-Cholbi, A., & Delhom, I. (2022). Analysis of predictors of stress during confinement by COVID-19 in Spain. *Actas Espanolas de Psiquiatria*, *50*(4), 169-177.	x		
Lacomba-Trejo, L., Calderon-Cholbib, A., & Delhom, I. (2022). Analysis of stress predictors during confinement by COVID-19 in Spain. *Actas Espanolas De Psiquiatria*, *50*(4), 169-177. <Go to ISI>://WOS:000830425500001	x		
Lades, L. K., Laffan, K., Daly, M., & Delaney, L. (2020). Daily emotional well-being during the COVID-19 pandemic. *British Journal of Health Psychology*, *25*(4), 902-911.	x		

Laessle, C., Schneider, J., Pisarski, P., Fichtner-Feigl, S., & Janigen, B. (2021). Experiences and Short-Term Outcomes of Kidney Transplantation During the Coronavirus Disease 2019 Pandemic From a Medium-Volume Transplantation and Superregional Coronavirus Disease 2019 Treatment Center [Comparative Study]. *Transplantation Proceedings*, *53*(4), 1146-1153.			x	x
Lai, X., Wang, X., Yang, Q., Xu, X., Tang, Y., Liu, C., Tan, L., Lai, R., Wang, H., Zhang, X., Zhou, Q., & Chen, H. (2020). Will healthcare workers improve infection prevention and control behaviors as COVID-19 risk emerges and increases, in China? *Antimicrobial Resistance & Infection Control*, *9*(1), 83.	x			
Lardier, D. T., Zuhl, M. N., Holladay, K. R., Amorim, F. T., Heggenberger, R., & Coakley, K. E. (2022). A latent class analysis of mental health severity and alcohol consumption: Associations with covid-19-related quarantining, isolation, suicidal ideations, and physical activity. *International Journal of Mental Health and Addiction*,		x		
Lasalvia, A., Rigon, G., Rugiu, C., Negri, C., Del Zotti, F., Amaddeo, F., & Bonetto, C. (2022). The psychological impact of COVID-19 among primary care physicians in the province of Verona, Italy: a cross-sectional study during the first pandemic wave [Observational Study]. *Family Practice*, *39*(1), 65-73.	x			

Laura, R. P., Jonatan, G. C., Banwell, G., Belen, O. A., Gabriel, G. N., & Andres, R. C. (2022). Experiences of Patients with Rheumatoid Arthritis during and after Covid-19 Induced Quarantine in Terms of Physical Activity and Health Status: A Qualitative Study. *Journal of nursing management.*, *03*.	x	
Lautenbach, G., & Randell, N. (2020). THROUGH THE COVID-19 LOOKING GLASS: COPING SKILLS FOR STEM EDUCATORS IN THE TIME OF A PANDEMIC AND BEYOND. *Journal of Baltic Science Education*, *19*(6A), 1068-1077. https://doi.org/10.33225/jbse/20.19.1068	x	
Lay-Yee, R., Campbell, D., & Milne, B. (2022). Social attitudes and activities associated with loneliness: Findings from a New Zealand national survey of the adult population. *Health & Social Care in the Community*, *30*(3), 1120-1132. https://doi.org/10.1111/hsc.13351	x	
Layek, A. K., Ghosh, N., Biswas, J., & Bhaduri, S. (2021). Self-isolation of healthcare workers during covid-19 pandemic in a tertiary care center - association between their sleep quality, anxiety status and social capital. *Indian Journal of Forensic Medicine and Toxicology*, *15(3)*, 129-136.	x	
Lee, K. M., Ko, H. J., Lee, G. H., Kim, A. S., & Lee, D. W. (2021). A Well-Structured Follow-Up Program is Required after Recovery from Coronavirus Disease 2019 (COVID-19); Release from Quarantine is Not the End of Treatment. *Journal of Clinical Medicine*, *10*(11), Article 2329. https://doi.org/10.3390/jcm10112329		x

Lee, K. S., Sung, H. K., Lee, S. H., Hyun, J., Kim, H., Lee, J. S., Paik, J. W., Kim, S. J., Sohn, S., & Choi, Y. K. (2022). Factors Related to Anxiety and Depression Among Adolescents During COVID-19: A Web-Based Cross-Sectional Survey. *Journal of Korean Medical Science*, *37*(25), e199.	x										
Lee, Y., Kim, B. W., Kim, S. W., Son, H., Park, B., Lee, H., You, M., & Ki, M. (2021). Precautionary Behavior Practices and Psychological Characteristics of COVID-19 Patients and Quarantined Persons *International Journal of Environmental Research and Public Health*, *18(11) (no pagination)*,					x						x
Legido-Quigley, H., Asgari, N., Teo, Y. Y., Leung, G. M., Oshitani, H., Fukuda, K., Cook, A. R., Hsu, L. Y., Shibuya, K., & Heymann, D. (2020). Are high-performing health systems resilient against the COVID-19 epidemic? [Note]. *The Lancet*,				x							
Lei, L., Huang, X., Zhang, S., Yang, J., Yang, L., & Xu, M. (2020). Comparison of Prevalence and Associated Factors of Anxiety and Depression Among People Affected by versus People Unaffected by Quarantine During the COVID-19 Epidemic in Southwestern China [Comparative Study]. *Medical Science Monitor*, *26*, e924609.	x										
Lelisho, M. E., & Tareke, S. A. (2022). Prevalence and Associated Factors of Depressive Symptoms Among Mizan-Tepi University Students During the COVID-19 Pandemic. *Journal of racial and ethnic health disparities.*, *28*.	x										

Levere, M., Rowan, P., & Wysocki, A. (2021). The Adverse Effects of the COVID-19 Pandemic on Nursing Home Resident Well-Being [Research Support, Non-U.S. Gov't]. *Journal of the American Medical Directors Association*, *22*(5), 948-954.e942.		x		
Levy, A. G., Thorpe, A., Scherer, L. D., Scherer, A. M., Drews, F. A., Butler, J. M., Burpo, N., Shoemaker, H., Stevens, V., & Fagerlin, A. (2022). Misrepresentation and Nonadherence Regarding COVID-19 Public Health Measures [Research Support, Non-U.S. Gov't]. *JAMA Network Open*, *5*(10), e2235837. 8		x		
Li, G. Q., Liu, H., Qiu, C. J., & Tang, W. J. (2022). Fear of COVID-19, prolonged smartphone use, sleep disturbances, and depression in the time of COVID-19: A nation-wide survey. *Frontiers in Psychiatry*, *13*, Article 971800. https://doi.org/10.3389/fpsyt.2022.971800		x		
Li, H., Peng, Y. Y., & Lu, J. P. (2021). Investigation and Analysis of 108 Cases of Home Isolated Patients With Mild COVID-19. *Disaster Medicine & Public Health Preparedness*, *15*(6), e8-e11.	x			x
Li, Y. F., Li, J., Yang, Z., Zhang, J., Dong, L. L., Wang, F. S., & Zhang, J. P. (2021). Gender Differences in Anxiety, Depression, and Nursing Needs Among Isolated Coronavirus Disease 2019 Patients. *Frontiers in Psychology*, *12*, Article 615909. https://doi.org/10.3389/fpsyg.2021.615909			x	

Liang, I. J., Perkin, O. J., McGuigan, P. M., Thompson, D., & Western, M. J. (2022). Feasibility and Acceptability of Home-Based Exercise Snacking and Tai Chi Snacking Delivered Remotely to Self-Isolating Older Adults During COVID-19 [Randomized Controlled Trial]. *Journal of Aging & Physical Activity*, *30*(1), 33-43.	x		
Lieberoth, A., Lin, S. Y., Stockli, S., Han, H., Kowal, M., Gelpi, R., Chrona, S., Tran, T. P., Jeftic, A., Rasmussen, J., Cakal, H., Milfont, T. L., Yamada, Y., Amin, R., Debove, S., Flis, I., Sahin, H., Turk, F., Yeh, Y. Y., . . . Dubrov, D. (2021). Stress and worry in the 2020 coronavirus pandemic: relationships to trust and compliance with preventive measures across 48 countries in the COVIDiSTRESS global survey. *Royal Society Open Science*, *8*(2), Article 200589. https://doi.org/10.1098/rsos.200589	x		
Lin, C., & Fu, X. H. (2022). A Cross-Sectional Study of Depression, Anxiety, and Insomnia Symptoms in People in Quarantine During the COVID-19 Epidemic. *International Journal of Public Health*, *67*, Article 1604723. https://doi.org/10.3389/ijph.2022.1604723		x	x
Lin, Y. L., et al. (2021). "Quarantine for the coronavirus disease (COVID-19) in Wuhan city: Support, understanding, compliance and psychological impact among lay public." Journal of Psychosomatic Research 144.	x		
Linas, B. P., et al. (2021). "A clash of epidemics: Impact of the COVID-19 pandemic response on opioid overdose." Journal of Substance Abuse Treatment 120: 108158.	x		

Lindoso, L., et al. (2021). "Physical and mental health during COVID-19 quarantine in adolescents with chronic immunocompromised conditions and inflammatory bowel disease." Journal of Crohns & Colitis 15: S579-S579.	x		
Lindoso, L., et al. (2022). "Physical and mental health impacts during COVID-19 quarantine in adolescents with preexisting chronic immunocompromised conditions." Jornal De Pediatria 98(4): 350-361.	x		
Ling, M. S., et al. "Emotional disturbance and risk factors among COVID-19 confirmed cases in isolation hotels." International Journal of Mental Health Nursing.		x	x
Ling, M. S., et al. "Emotional disturbance and risk factors among COVID-19 confirmed cases in isolation hotels." International Journal of Mental Health Nursing.		x	x
Lionetti, F., et al. (2021). "On the role of moderators on children's sleep health in response to COVID-19." Journal of Clinical Sleep Medicine 17(2): 353-354.	x		
Lipai, T. P. (2020). "[the Covid-19 Pandemic: Depression, Anxiety, Stigma and Impact on Mental Health]." Problemy Sotsialnoi Gigieny i Istoriia Meditsiny 28(5): 922-927.	x		
Lippi, G., et al. (2020). "Physical inactivity and cardiovascular disease at the time of coronavirus disease 2019 (COVID-19)." European Journal of Preventive Cardiology 27(9): 906-908.	x		

Lipskaya-Velikovsky, L. (2021). "COVID-19 Isolation in Healthy Population in Israel: Challenges in Daily Life, Mental Health, Resilience, and Quality of Life." International Journal of Environmental Research & Public Health [Electronic Resource] 18(3): 23.	x	
Littman, R., et al. (2020). "Impact of COVID-19 on obsessive-compulsive disorder patients." Psychiatry & Clinical Neurosciences 74(12): 660-661.	x	
Liu, C. H., et al. (2022). "Concerns about the social climate, finances, and COVID-19 risk on depression and anxiety: An analysis on U.S. young adults across two waves." Journal of Psychiatric Research 148: 286-292.	x	
Liu, C., et al. (2022). "Influence of landscape outside the window on the anxiety level of self-separation people during COVID-19." International Journal of Low-Carbon Technologies 17: 678-685.	x	
Liu, H. Q., et al. (2021). "Clinical characteristics and follow-up analysis of 324 discharged COVID-19 patients in Shenzhen during the recovery period." International Journal of Medical Sciences 18(2): 347-355.	x	
Liu, J. J., et al. (2020). "Mental health considerations for children quarantined because of COVID-19." Lancet Child & Adolescent Health 4(5): 347-349.		x
Liu, J., et al. (2022). "Compliance with COVID-19-preventive behaviours among employees returning to work in the post-epidemic period." BMC Public Health 22(1).	x	

Liu, L., et al. (2021). "Urban-rural disparities in mental health problems related to COVID-19 in China." General Hospital Psychiatry 69: 119-120.	x	
Liu, M., et al. (2022). "Associations among perceived built environment, attitudes, walking behavior, and physical and mental state of college students during COVID-19." Travel Behaviour and Society 28: 170-180.	x	
Liu, Q., et al. (2021). "The prevalence of behavioral problems among school-aged children in home quarantine during the COVID-19 pandemic in china." Journal of Affective Disorders 279: 412-416.	x	
Liu, S. M., et al. (2021). "Gendered Factors Associated with Preventive Behaviors and Mental Health among Chinese Adults during the COVID-19 Pandemic Home Quarantine." Sustainability 13(19).	x	
Liu, W. and J. Liu (2021). "Living with COVID-19: A phenomenological study of hospitalised patients involved in family cluster transmission." BMJ Open 11(2) (no pagination).	x	
Liu, X. L., et al. (2021). "ANALYSIS OF EMOTIONAL STATE AND ASSOCIATED FACTORS IN PATIENTS WITH FEVER DURING ISOLATION STAY IN THE COVID-19 PANDEMIC." Acta Medica Mediterranea 37(2): 1057-1062.		x
Liu, Y. and S. Mattke (2020). "Association between state stay-at-home orders and risk reduction behaviors and mental distress amid the COVID-19 pandemic." Preventive Medicine 141: 106299.	x	

Liu, Y. E., et al. (2022). "COVID-19 Preventive Measures in Northern California Jails: Perceived Deficiencies, Barriers, and Unintended Harms." Frontiers in Public Health 10: 854343.				x	x
Liu, Y. P., et al. (2021). "Using Mindfulness to Reduce Anxiety and Depression of Patients With Fever Undergoing Screening in an Isolation Ward During the COVID-19 Outbreak." Frontiers in Psychology 12.		x			
Liu, Y., et al. (2020). "Awareness of mental health problems in patients with coronavirus disease 19 (COVID-19): A lesson from an adult man attempting suicide." Asian Journal of Psychiatry Vol 51 2020, ArtID 102106 51.			x		
Liu, Y., et al. (2020). "Perceived Discrimination and Mental Distress Amid the COVID-19 Pandemic: Evidence From the Understanding America Study." American Journal of Preventive Medicine 59(4): 481-492.	x				
Liu, Z., et al. (2021). "Mobile application-based behaviour change techniques to encourage quarantine compliance during the COVID-19 pandemic." Public Health 197: e6-e7.	x				
Llerena, A. M. A. and A. M. P. Lascano (2022). "Sustainability of employment during COVID-19, in Ecuador." Vision Gerencial 21(1): 7-19.	x				
Llibre-Guerra, J. J., et al. (2020). "The impact of COVID-19 on mental health in the Hispanic Caribbean region." International Psychogeriatrics 32(10): 1143-1146.	x				
Lo Presti, S., et al. (2021). "Psychological precursors of individual differences in COVID-19 lockdown adherence: Moderated-moderation by personality and moral cognition measures." Personality and Individual Differences Vol 182 2021, ArtID 111090 182.	x				

Lochner, C., et al. (2022). "The COVID-19 pandemic and problematic usage of the internet: Findings from a diverse adult sample in South Africa." Journal of Psychiatric Research 153: 229-235.		x								
Loerinc, L. B., et al. (2021). "Discharge characteristics and care transitions of hospitalized patients with COVID-19." Healthcare 9(1) (no pagination).		x								
Logrosa, G., et al. (2022). "Integrating Risk Assessment and Decision-Making Methods in Analyzing the Dynamics of COVID-19 Epidemics in Davao City, Mindanao Island, Philippines." Risk Analysis 42(1): 105-125.		x								
Lohana, S., et al. (2021). "Psychological impact of Corona Virus Disease on genera population in Karachi." World Family Medicine 19(6): 59-70.		x								
Lohiniva, A. L., et al. (2021). "Learning about COVID-19-related stigma, quarantine and isolation experiences in Finland." PLoS ONE 16(4).	x						x			
Lokuge, K., et al. (2022). "Opening up safely: public health system requirements for ongoing COVID-19 management based on evaluation of Australia's surveillance system performance." BMC Medicine 20(1).							x			x
Longobardi, C., et al. (2020). "COVID-19 emergency: Social distancing and social exclusion as risks for suicide ideation and attempts in adolescents." Frontiers in Psychology Vol 11 2020, ArtID 551113 11.						x				
Loomis, C., et al. (2021). "Global discourse and local coping and hoping during the COVID-19 pandemic." Community Psychology in Global Perspective 7(1): 1-12.						x				

Lopes, A. R. and O. K. Nihei (2021). "Depression, anxiety and stress symptoms in Brazilian university students during the COVID-19 pandemic: Predictors and association with life satisfaction, psychological well-being and coping strategies." PLoS ONE [Electronic Resource] 16(10): e0258493.	x
Lopes, B., et al. (2020). "Paranoia, hallucinations and compulsive buying during the early phase of the COVID-19 outbreak in the United Kingdom: A preliminary experimental study." Psychiatry Research 293: 113455.	x
Lopez Steinmetz, L. C., et al. (2021). "A longitudinal study on depression and anxiety in college students during the first 106-days of the lengthy Argentinean quarantine for the COVID-19 pandemic." Journal of Mental Health.	x
Lopez Steinmetz, L. C., et al. (2021). "Suicidal risk and impulsivity-related traits among young Argentinean college students during a quarantine of up to 103-day duration: Longitudinal evidence from the COVID-19 pandemic." Suicide & Life-Threatening Behavior 51(6): 1175-1188.	x
Lopez Steinmetz, L. C., et al. (2022). "Levels and predictors of depression, anxiety, and suicidal risk during COVID-19 pandemic in Argentina: the impacts of quarantine extensions on mental health state." Psychology Health & Medicine 27(1): 13-29.	x
Lopez-Aymes, G., et al. (2021). "A mixed methods research study of parental perception of physical activity and quality of life of children under home lock down in the COVID-19 pandemic." Frontiers in Psychology Vol 12 2021, ArtID 649481 12.	x

Lopez-Bueno, R., et al. (2020). "Association Between Current Physical Activity and Current Perceived Anxiety and Mood in the Initial Phase of COVID-19 Confinement." Frontiers in Psychiatry 11 (no pagination).	x
Lopez-Bueno, R., et al. (2020). "Health-Related Behaviors Among School-Aged Children and Adolescents During the Spanish Covid-19 Confinement." Frontiers in Pediatrics 8 (no pagination).	x
Lopez-Carral, H., et al. (2020). "Subjective ratings of emotive stimuli predict the impact of the COVID-19 quarantine on affective states." PLoS ONE 15(8 August) (no pagination).	x
Lopez-Gutierrez, C. J., et al. (2021). "PSYCHOLOGICAL DISCOMFORT AND STRESS DURING CONFINEMENT DUE TO THE COVID-19 PANDEMIC. COMPARATIVE STUDY BETWEEN ATHLETES AND NON-ATHLETES." Revista Iberoamericana De Psicologia Del Ejercicio Y El Deporte 16(4).	x
Lopez-Medina, C., et al. (2021). "Treatment adherence during the COVID-19 pandemic and the impact of confinement on disease activity and emotional status: A survey in 644 rheumatic patients." Joint, Bone, Spine: Revue du Rhumatisme 88(2): 105085.	x
Lopez-Moreno, M., et al. (2020). "Physical and psychological effects related to food habits and lifestyle changes derived from covid-19 home confinement in the spanish population." Nutrients 12(11): 1-17.	x

Lopez-Nunez, M., et al. (2021). "Individual differences, personality, social, family and work variables on mental health during COVID-19 outbreak in Spain." Personality and Individual Differences Vol 172 2021, ArtID 110562 172.	x
Lopez-Serrano, J., et al. (2021). "Psychological impact during COVID-19 lockdown in children and adolescents with previous mental health disorders." Revista de Psiquiatria y Salud Mental.	x
Lopez, D. M. L., et al. (2021). "Resilience and psychological distress in pregnant women during quarantine due to the COVID-19 outbreak in Spain: a multicentre cross-sectional online survey." Journal of Psychosomatic Obstetrics & Gynecology 42(2): 115-122.	x
Lopez, G., et al. (2021). "Impact of extended quarantine during the COVID-19 pandemic on Parkinson's disease (PD) patients." Movement Disorder 36(SUPPL 1): S144-S145.	x
Lopez, J., et al. (2021). "COVID-19 pandemic lockdown responses from an emotional perspective: Family function as a differential pattern among older adults." Behavioral Psychology 29(2): 331-344.	x
Lopez, P. J. T., et al. (2021). "Changes in adherence to the Mediterranean diet observed in a Spanish population during confinement for the SARS-CoV-2 pandemic." Nutricion Hospitalaria 38(1): 109-120.	x
Lorant, V., et al. (2021). "Psychological distress associated with the COVID-19 pandemic and suppression measures during the first wave in Belgium." BMC Psychiatry 21(1): 112.	x

Lord, S. A. (2022). "COVID couple therapy: Telehealth and somatic action techniques." Australian and New Zealand Journal of Family Therapy 43(2): 197-209.		x	
Lou, Q., et al. (2020). "Home quarantine compliance is low in children with fever during COVID-19 epidemic." World Journal of Clinical Cases 8(16): 3465-3473.	x		x
Lourens, H. and B. Watermeyer (2021). "The invisible lockdown: Reflections on disability during the time of the coronavirus pandemic." Disability & Society: No Pagination Specified.		x	
Louvardi, M., et al. (2020). "Mental health in chronic disease patients during the COVID-19 quarantine in Greece." Palliative & Supportive Care 18(4): 394-399.		x	
Lowe, C., et al. (2021). "Communication technology use, age and depressive symptoms during covid-19." Psychosomatic Medicine 83(7): A67.		x	
Lowe, C., et al. (2021). "Communication technology use, age and depressive symptoms during covid-19." Psychosomatic Medicine 83(7): A67.		x	
Lozano-Diaz, A., et al. (2020). "Impacts of COVID-19 Confinement among College Students: Life Satisfaction, Resilience and Social Capital Online." International Journal of Sociology of Education 9: 79-104.		x	
Lozano-Diaz, A., et al. (2020). "Impacts of COVID-19 Confinement among College Students: Life Satisfaction, Resilience and Social Capital Online." International Journal of Sociology of Education 9: 79-104.		x	

Lucas, T. C. D., et al. (2021). "Engagement and adherence trade-offs for SARS-CoV-2 contact tracing." Philosophical Transactions of the Royal Society of London - Series B: Biological Sciences 376(1829): 20200270.			x		x
Lucas, T., et al. (2022). "Justice beliefs and cultural values predict support for COVID-19 vaccination and quarantine behavioral mandates: A multilevel cross-national study." Translational Behavioral Medicine 12(2): 284-290.				x	x
Lucchini, L., et al. (2021). "Living in a pandemic: changes in mobility routines, social activity and adherence to COVID-19 protective measures." Scientific Reports 11(1): 24452.	x				
Lucibello, K. M., et al. (2021). "#quarantine15: A content analysis of Instagram posts during COVID-19." Body Image 38: 148-156.	x				
Lugo-Gonzalez, I. V., et al. (2020). "COVID-19 perception and preventive behaviors: A descriptive, comparative study by severity and perceived risk." Salud Mental 43(6): 285-292.	x				
Luis, E., et al. (2021). "Relationship between self-care activities, stress and well-being during COVID-19 lockdown: a cross-cultural mediation model." BMJ Open 11(12): e048469.	x				
Luna, A. and M. Chong (2021). WORKSHOP: 2020 Our resilience - Our Home Class experience. 5th IEEE World Conference on Engineering Education (EDUNINE), Univ Galileo, Guatemala City, GUATEMALA.	x				
Lund, E. M., et al. (2020). "The COVID-19 pandemic, stress, and trauma in the disability community: A call to action." Rehabilitation Psychology 65(4): 313-322.		x			

Luning-Koster, M. N., et al. (2022). "[Experience of first COVID-19 patients in Northern part of the Netherlands: Information provision, source investigation, contact tracing and home isolation]." Nederlands Tijdschrift voor Geneeskunde 166(01): 27.		x			
Lunn, P. D., et al. (2020). "Using decision aids to support self-isolation during the covid-19 pandemic." Psychology & Health: No Pagination Specified.			x	x	
Luo, X., et al. (2020). "The psychological impact of quarantine on coronavirus disease 2019 (COVID-19)." Psychiatry Research Vol 291 2020, ArtID 113193 291.	x				
Lusida, M. A. P., et al. (2022). "Prevalence of and risk factors for depression, anxiety, and stress in non-hospitalized asymptomatic and mild COVID-19 patients in East Java province, Indonesia." PLoS ONE [Electronic Resource] 17(7): e0270966.	x				
Lusida, M. A. P., et al. (2022). "The Impact of Facilitated Quarantine on Mental Health Status of Non-Severe COVID-19 Patients." Disaster Medicine and Public Health Preparedness 16(5): 1751-1752.			x		x
Lyne, J., et al. (2020). "COVID-19 from the perspective of urban and rural general adult mental health services." Irish Journal of Psychological Medicine 37(3): 181-186.	x				
M, S. and G. N (2021). "Quarantined individual's behavior- a model evidence from covid -19 pandemic." Journal of Human Behavior in the Social Environment: No Pagination Specified.	x				

M, S. and G. N (2021). "Quarantined individual's behavior- a model evidence from covid -19 pandemic." Journal of Human Behavior in the Social Environment: No Pagination Specified.		x								
Ma, Y. F., et al. (2020). "Prevalence of depression and its association with quality of life in clinically stable patients with COVID-19." Journal of Affective Disorders 275: 145-148.			x							
Machuca, M., et al. (2022). "Telematic Follow-up in Mental Health (MH) for SARS-CoV2 (+) users at home: A dam for the fifth wave?" Journal of Psychosomatic Research Conference: Ninth Annual Scientific Conference of the European Association of Psychosomatic Medicine (EAPM). Vienna Austria. 157 (no pagination).	x						x			
MacIntosh, B. J., et al. (2021). "Brain structure and function in people recovering from COVID-19 after hospital discharge or self-isolation: a longitudinal observational study protocol." CMAJ open 9(4): E1114-E1119.							x			x
Mader, E. and G. Dori (2020). "Patients with the novel SARS-cov-2 disease require a novel standard of care-med-psych." Psychosomatics: Journal of Consultation and Liaison Psychiatry 61(5): 578-579.						x				
Mader, E., et al. (2021). "The role of religious and spiritual aid in quarantine hospitalization due to SARS-CoV-2." Journal of the Academy of Consultation-Liaison Psychiatry 62(2): 260-261.						x				
Madrigal, L. and A. Blevins (2022). ""I hate it, it's ruining my life": College students' early academic year experiences during the COVID-19 pandemic." Traumatology 28(3): 325-335.		x								

Magamela, M. R., et al. (2021). "Covid-19 consequences on mental health: An african perspective." South African Journal of Psychiatry 27: 1-2.	x				
Maggi, G., et al. (2021). "Mental health status of Italian elderly subjects during and after quarantine for the COVID-19 pandemic: a cross-sectional and longitudinal study." Psychogeriatrics:The Official Journal of the Japanese Psychogeriatric Society 21(4): 540-551.	x				
Mahendru, K., et al. (2021). "Effect of Meditation and Breathing Exercises on the Well-being of Patients with SARS-CoV-2 Infection under Institutional Isolation: A Randomized Control Trial." Indian Journal of Palliative Care 27(4): 490-494.			x		x
Mahmoud, F. M. (2020). "Shared Loneliness During COVID-19." Journal of Graduate Medical Education 12(4): 412-413.			x	x	
Maitra, A. (2020). "Holding up." New England Journal of Medicine 383(26): 2498-2499.		x			
Majumdar, S., et al. (2022). "Worry, Perceived Discrimination, Lifestyle Changes, and Protective Factors During COVID-19: A Study With Recovering Patients in Delhi, India." Sage Open 12(1).			x		x
Malathesh, B. C., et al. (2020). "Overview of mental health issues of COVID-19: Need of the hour." General Psychiatry 33(3): 1-2.	x				
Maleki, M., et al. (2022). "Social Behavior and COVID-19: Analysis of the Social Factors behind Compliance with Interventions across the United States." International Journal of Environmental Research & Public Health [Electronic Resource] 19(23): 25.	x				

Malhotra, N., et al. (2020). Medico-Legal Update 20(4): 1540-1544.		x
Malhotra, V., et al. (2022). "Prevalence and Predictors of Depression, Anxiety and Stress among Elderly in the aftermath of COVID-19: A Quantitative Study from Central India." medRxiv. 12.	x	
Malkawi, S. H., et al. (2021). "COVID-19 Quarantine-Related Mental Health Symptoms and their Correlates among Mothers: A Cross Sectional Study." Maternal & Child Health Journal 25(5): 695-705.	x	
Malmquist, A., et al. (2022). "Queers in quarantine: Young LGBTQ+ people's experiences during the COVID-19 pandemic in Sweden." Scandinavian journal of psychology. 25.	x	
Malta, D. C., et al. (2021). "Factors associated with increased cigarette consumption in the Brazilian population during the COVID-19 pandemic." Cadernos De Saude Publica 37(3): e00252220.	x	
Mamedov, M. N., et al. (2022). "Psychosomatic status analysis of patients with chronic non-communicable diseases (CNCDs) in Russia and CIS countries during self-isolation. [Russian]." Profilakticheskaya Meditsina 25(1): 29-34.	x	
Mandal, A. (2021). "Psychological aspects in terms of physical, mental and sociological factors during worldwide corona crisis." Indian Journal of Forensic Medicine and Toxicology 15(2): 1-5.	x	

Mangrio, E., et al. (2022). "Working With Refugees' Health During COVID-19-The Experience of Health- and Social Care Workers in Sweden." Frontiers in Public Health 10: 811974.	x										
Mani, A., et al. (2022). "Correlation Between Anger, Sleep Quality, and Indoor Activities During COVID-19 Quarantine." Shiraz E Medical Journal 23(5) (no pagination).	x										
Mansoor, T., et al. (2020). "'Surviving COVID-19': Illness Narratives of Patients and Family Members in Pakistan." Annals of King Edward Medical University Lahore Pakistan 26: 157-164.						x					x
Mantymaki, M., et al. (2022). "Coping with pandemics using social network sites: A psychological detachment perspective to COVID-19 stressors." Technological Forecasting and Social Change 179.	x										
Manzano-Leon, A., et al. (2022). "Gamification and family leisure to alleviate the psychological impact of confinement due to COVID-19." Children & Society 36(4): 433-449.	x										
Marazziti, D. and S. M. Stahl (2020). "The relevance of COVID-19 pandemic to psychiatry." World Psychiatry 19(2): 261.					x						
Marchini, S., et al. (2021). "Study of resilience and loneliness in youth (18-25 years old) during the COVID-19 pandemic lockdown measures." Journal of Community Psychology 49(2): 468-480.	x										
Marcus, P. H., et al. (2021). Current Psychiatry 20(1): 28-33.					x						
Margraf, J., et al. (2021). "Adherence to behavioral Covid-19 mitigation measures strongly predicts mortality." PLoS ONE 16(3 March) (no pagination).	x										

Marholz, P. F. O., et al. (2022). "Levels of Trait Anxiety and Well-being in Chilean professional soccer players during the COVID-19 quarantine." Retos-Nuevas Tendencias En Educacion Fisica Deporte Y Recreacion(44): 1037-1044.		x	
Mari, J. J. and M. A. Oquendo (2020). "Mental health consequences of COVID-19: The next global pandemic." Trends in Psychiatry and Psychotherapy 42(3): 219-220.		x	
Mariani, D., et al. (2020). "Habits in the time of Coronavirus. [Italian]." Giornale di Clinica Nefrologica e Dialisi 32(1): 69-72.		x	
Maric, N. P., et al. (2022). "Covid-19-related stressors, mental disorders, depressive and anxiety symptoms: a cross-sectional, nationally-representative, face-to-face survey in Serbia." Epidemiology & Psychiatric Science 31: e36.	x		x
Marin, G. H., et al. (2022). "[Implementation of a support system for isolated elderly people to limit the consequences of the Covid-19 epidemic in Buenos Aires]." Soins. Gerontologie 27(154): 30-38.		x	
Marini, C. M., et al. (2020). "Aging veterans' mental health and well-being in the context of COVID-19: The importance of social ties during physical distancing." Psychological Trauma: Theory, Research, Practice, and Policy 12(S1): S217-S219.		x	
Marinthe, G., et al. (2020). "Looking out for myself: Exploring the relationship between conspiracy mentality, perceived personal risk, and COVID-19 prevention measures." British Journal of Health Psychology 25(4): 957-980.		x	

Mark, E., et al. "The appropriateness of the decision to quarantine healthcare workers exposed to a severe acute respiratory coronavirus virus 2 (SARS-CoV-2)-positive coworker based on national guidelines." Infection Control and Hospital Epidemiology.			x	x
Markiewicz-Gospodarek, A., et al. (2022). "The Relationship between Mental Disorders and the COVID-19 Pandemic-Course, Risk Factors, and Potential Consequences." International Journal of Environmental Research and Public Health 19(15) (no pagination).	x			
Markkanen, P., et al. (2021). ""It changed everything": The safe Home care qualitative study of the COVID-19 pandemic's impact on home care aides, clients, and managers." BMC Health Services Research 21(1): 1055.	x			
Marques, I. G., et al. (2021). "Lessons learned from a home-based exercise program for adolescents with pre-existing chronic diseases during the covid-19 quarantine in brazil." Clinics 76 (no pagination).	x			
Marroquin, B., et al. (2020). "Mental health during the COVID-19 pandemic: Effects of stay-at-home policies, social distancing behavior, and social resources." Psychiatry Research 293 (no pagination).	x			
Marsden, L., et al. (2022). "Daily testing of contacts of SARS-CoV-2 infected cases as an alternative to quarantine for key workers in Liverpool: A prospective cohort study." eClinicalMedicine 50 (no pagination).			x	x
Marshall, A. L. and A. Wolanskyj-Spinner (2020). "COVID-19: Challenges and Opportunities for Educators and Generation Z Learners." Mayo Clinic Proceedings 95(6): 1135-1137.		x		

Martarelli, C. S. and W. Wolff (2020). "Too bored to bother? Boredom as a potential threat to the efficacy of pandemic containment measures." Humanities & Social Sciences Communications 7(1).			x		
Martin-Krumm, C., et al. (2020). "Optimism and COVID-19: A resource to support people in confinement?" Annales Medico-Psychologiques 178(7): 728-737.		x			
Martin, A. F., et al. (2021). "Engagement with daily testing instead of self-isolating in contacts of confirmed cases of SARS-CoV-2." BMC Public Health 21(1): 1067.	x				x
Martin, S., et al. (2021). "New onset of child maladaptive behaviors during the COVID-19 quarantine: An examination of prevalence and risk factors." Psychosomatic Medicine 83(7): A6-A7.		x			
Martinez-Rodriguez, T. Y., et al. (2021). "Physical activity as self-regulating behavior of perceived anxiety and dysfunctional patterns of dietary intake during isolation due to COVID-19 among Latin Americans." Revista Chilena De Nutricion 48(3): 347-354.		x			
Martinez-Taboas, A., et al. (2021). "COVID stress scales: The psychosocial impact of COVID-19 in Latinos/as." Revista Puertorriquena de Psicologia 32(1): 90-103.		x			
Martinez, E. Z., et al. (2020). "Physical activity in periods of social distancing due to COVID-19: a cross-sectional survey." Ciencia & Saude Coletiva 25(suppl 2): 4157-4168.		x			
Martino, E., et al. "Housing Vulnerability and COVID-19 Outbreaks: When Crises Collide." Urban Policy and Research.				x	x

Martins Van Jaarsveld, G. (2020). "The Effects of COVID-19 Among the Elderly Population: A Case for Closing the Digital Divide." Frontiers in Psychiatry 11 (no pagination).	x		
Martins, C. R., et al. (2021). "Parents' mental health and children's emotional regulation during the COVID-19 pandemic." Psicologia: Teoria e Pratica 23(1): 1-19.	x		
Martos Martinez-Caja, A., et al. (2022). "Pet ownership, feelings of loneliness, and mood in people affected by the first COVID-19 lockdown." Journal of Veterinary Behavior 57: 52-63.	x		
Maruta, N. A., et al. (2021). "Psychological Factors and Consequences of Psychosocial Stress during the Pandemic." Wiadomosci Lekarskie 74(9 cz 1): 2175-2181.	x		
Mason, T. B., et al. (2021). "Eating to Cope With the COVID-19 Pandemic and Body Weight Change in Young Adults." Journal of Adolescent Health 68(2): 277-283.	x		
Matnazarova, G., et al. (2020). "The new coronavirus-cOvid-19 in Uzbekistan." International Journal of Pharmaceutical Research 12(4): 548-556.		x	x
Matovic, S., et al. (2022). "Group-Based Trajectories and Predictors of Psychological Distress during Covid-19: A Longitudinal Study of Older Adults in Quebec." Psychosomatic Medicine 84(5): A4-A5.	x		
Mattioli, A. V., et al. (2020). "COVID-19 pandemic: the effects of quarantine on cardiovascular risk." European Journal of Clinical Nutrition 74(6): 852-855.	x		

Mattioli, A. V., et al. (2020). "Obesity risk during collective quarantine for the COVID-19 epidemic." Obesity Medicine 20 (no pagination).	x			
Mattioli, A. V., et al. (2020). "Practical tips for prevention of cardiovascular disease in women after quarantine for COVID-19 disease." Acta Bio-Medica de l Ateneo Parmense 91(4): e2020127.	x			
Mattioli, A. V., et al. (2020). "Quarantine during COVID-19 outbreak: Changes in diet and physical activity increase the risk of cardiovascular disease." Nutrition Metabolism & Cardiovascular Diseases 30(9): 1409-1417.		x		
Mattioli, A. V., et al. (2021). "Lifestyle and Stress Management in Women During COVID-19 Pandemic: Impact on Cardiovascular Risk Burden." American Journal of Lifestyle Medicine 15(3): 356-359.	x			
Maugeri, G., et al. (2020). "The impact of physical activity on psychological health during Covid-19 pandemic in Italy." Heliyon 6(6).	x			
Maximova, O. A., et al. (2021). "SOCIAL FACTORS, PARAMETERS AND EFFECTS OF FORCED SELF-ISOLATION IN RUSSIA." Ad Alta-Journal of Interdisciplinary Research 11(2): 57-61.	x			
Mazza, M., et al. (2020). "Danger in danger: Interpersonal violence during COVID-19 quarantine." Psychiatry Research 289: 113046.		x		
Mbunge, E. (2020). "Effects of COVID-19 in South African health system and society: An explanatory study." Diabetes & Metabolic Syndrome 14(6): 1809-1814.			x	x

Mbunge, E., et al. (2021). "Ethics for integrating emerging technologies to contain COVID-19 in Zimbabwe." Human Behavior and Emerging Technologies 3(5): 876-890.			x	x
McAiney, C., et al. (2021). "In their own words: How COVID-19 has impacted the well-being of persons living with dementia in the community." Canadian Journal on Aging 40(4): 543-553.	x			
McCarron, N., et al. (2021). "Addressing isolation and quality of life during COVID-19." Journal of Applied Research in Intellectual Disabilities 34(5): 1221-1221.	x			
McCarron, N., et al. (2021). "Addressing isolation and quality of life during COVID-19." Journal of Applied Research in Intellectual Disabilities 34(5): 1221-1221.		x		
McCarron, R. H., et al. (2021). "Factors in psychiatric admissions: Before and during the COVID-19 pandemic." Clinical Neuropsychiatry: Journal of Treatment Evaluation 18(5): 270-277.	x			
McCarthy, E., et al. (2022). "New-onset and exacerbated insomnia symptoms during the COVID-19 pandemic in US military veterans: A nationally representative, prospective cohort study." Journal of Sleep Research 31(1): e13450.	x			
McCluskey, G., et al. (2021). "School closures, exam cancellations and isolation: The impact of Covid-19 on young people's mental health." Emotional & Behavioural Difficulties 26(1): 46-59.	x			
McHill, A. W. and E. D. Chinoy (2020). "Utilizing the National Basketball Association's COVID-19 restart "bubble" to uncover the impact of travel and circadian disruption on athletic performance." Scientific Reports 10(1).	x			

McIntyre, K., et al. (2021). "Use of a hotel for an emergency department homeless population requiring quarantine for SARS-CoV- 2." Academic Emergency Medicine 28(SUPPL 1): S284.			x	x
McKee, M. (2021). "The UK government tested the response to a coronavirus - Why are we only discovering this now?" The BMJ 375 (no pagination).		x		
McKinlay, A. R., et al. (2022). "'You're just there, alone in your room with your thoughts': a qualitative study about the psychosocial impact of the COVID-19 pandemic among young people living in the UK." BMJ Open 12(2): e053676.	x			
McLamore, Q., et al. (2022). "Trust in scientific information mediates associations between conservatism and coronavirus responses in the U.S., but few other nations." Scientific Reports 12(1): 3724.	x			
Meagher, B. R. and A. D. Cheadle (2020). "Distant from others, but close to home: The relationship between home attachment and mental health during COVID-19." Journal of Environmental Psychology Vol 72 2020, ArtID 101516 72.	x			
Mechili, E. A., et al. (2021). "Is the mental health of young students and their family members affected during the quarantine period? Evidence from the COVID-19 pandemic in Albania." Journal of Psychiatric & Mental Health Nursing 28(3): 317-325.	x			

Meda-Lara, R. M., et al. (2021). "Precautionary behaviors during the second and third phases of the covid-19 pandemic: Comparative study in the latin American population." International Journal of Environmental Research and Public Health 18(13) (no pagination).	x			
Meda-Lara, R. M., et al. (2022). "Psychological responses to COVID-19 in a Mexican population: an exploratory study during second and third phases." Psychology Health & Medicine 27(1): 257-264.	x			
Medina-Ortiz, O., et al. (2020). "[Sleep disorders as a result of the COVID-19 pandemic]." Revista Peruana de Medicina Experimental y Salud Publica 37(4): 755-761.		x		
Medina, M. S., et al. (2022). "Humanizing the isolation during the COVID-19 pandemic: The "Shortening Distances" Program." Revista Rol De Enfermeria 45(1): 16-24.	x			
Medina, N. S. and J. C. D. de Britto (2021). "EMOTIONAL STAPES OF THE STUDENTS OF DEGREE IN EARLY CHILHOOD AND PRIMARY EDUCATION DURING THE CONFINEMENT BY COVID-19." Barataria-Revista Castellano-Manchega De Ciencias Sociales(31): 57-70.	x			
Medvedev, V. E. and O. A. Dogotar (2021). "COVID-19 and mental health: Challenges and first conclusions. [Russian]." Nevrologiya, Neiropsikhiatriya, Psikhosomatika 12(6): 4-10.		x		
Meena, P., et al. (2021). "Mental health problems in health-care workers working in COVID-19 quarantine centers." Annals of Indian Psychiatry 5(2): 153-157.			x	x

Meena, S., et al. (2021). "Repatriation operation in South Australia during the COVID-19 pandemic: initial planning and preparedness." Communicable Diseases Intelligence 45: 27.			x	x
Mehrsafar, A. H., et al. (2020). "Addressing potential impact of COVID-19 pandemic on physical and mental health of elite athletes." Brain, Behavior, and Immunity 87: 147-148.		x		
Mejia, M. C., et al. (2022). "Cross-Sectional Survey of Smoking Patterns During the COVID-19 Pandemic in a Tobacco Cessation and Lung Cancer Screening Program." Ochsner Journal 22(1): 48-60.	x			
Melegari, M. G., et al. (2021). "Identifying the impact of the confinement of Covid-19 on emotional-mood and behavioural dimensions in children and adolescents with attention deficit hyperactivity disorder (ADHD)." Psychiatry Research 296 (no pagination).	x			
Mendes-Santos, C., et al. (2020). "Mitigating COVID-19 Impact on the Portuguese Population Mental Health: The Opportunity That Lies in Digital Mental Health." Frontiers in Public Health 8: 553345.		x		
Meng, Y., et al. (2020). "Mental health status of college students under regular prevention and control of coronavirus disease 2019 epidemic. [Chinese]." Academic Journal of Second Military Medical University 41(9): 958-965.	x			
Menon, V., et al. (2021). "COVID-19 pandemic and suicidality: Durkheim revisited." Australian and New Zealand Journal of Psychiatry 55(3): 324.		x		

Menouni, A., et al. (2022). "Mental health among the Moroccan population during SARS-CoV-2 outbreak: MAROCOVID study." Journal of Affective Disorders 308: 343-352.	x	
Meo, S. A., et al. (2020). "Covid-19 pandemic: Impact of quarantine on medical students' mental wellbeing and learning behaviors." Pakistan Journal of Medical Sciences 36(COVID19-S4): S43-S48.	x	
Merino-Munoz, P., et al. (2022). "Effects on the wellness state in period of confinement due to COVID-19 in professional soccer players." Mhsalud-Revista En Ciencias Del Movimiento Humano Y La Salud 19(1).	x	
Merino, M. D., et al. (2020). "Is It Possible to Find Something Positive in Being Confined Due to COVID-19? Implications for Well-Being." International Journal of Environmental Research & Public Health [Electronic Resource] 17(23): 05.	x	
Merino, M., et al. (2021). "What makes one feel eustress or distress in quarantine? An analysis from conservation of resources (COR) theory." British Journal of Health Psychology 26(2): 606-623.	x	
Messy, J. (2021). "COVID-19: The dual confinement." NPG Neurologie - Psychiatrie - Geriatrie 21(126): 391-394.		x
Messy, J. (2021). "COVID-19: The dual confinement." NPG Neurologie - Psychiatrie - Geriatrie 21(126): 391-394.	x	

Metin, B., et al. "Perceived Stress During the COVID-19 Pandemic Mediates the Association Between Self-quarantine Factors and Psychological Characteristics and Elevated Maladaptive Daydreaming." International Journal of Mental Health and Addiction.				x	x
Meza-de-Luna, M. E., et al. (2022). "Work-family conciliation with and without children, during confinement by COVID-19 in Mexico." Psicoperspectivas 21(2): 1-2.		x			
Mezzina, R., et al. (2020). "Mental health at the age of coronavirus: Time for change." Social Psychiatry and Psychiatric Epidemiology: The International Journal for Research in Social and Genetic Epidemiology and Mental Health Services 55(8): 965-968.			x		
Mgbedo, N. E., et al. (2022). "COVID-19 pandemic's impact on the student's sleep patterns at the University of Georgia." Sleep Medicine 100(Supplement 1): S73.		x			
Mi, L., et al. (2020). "Mental health and psychological impact of COVID-19: Potential high-risk factors among different groups." Asian Journal of Psychiatry 53 (no pagination).			x		
Migrant workers in China need emergency psychological interventions during the COVID-19 outbreak				x	x
Mijovic, F., et al. (2021). "The myth of pre-operative isolation during the COVID-19 pandemic." Science Progress 104(3): 368504211026155.	x				x
Mikolai, J., et al. (2020). "Intersecting household-level health and socio-economic vulnerabilities and the COVID-19 crisis: An analysis from the UK." SSM - Population Health 12 (no pagination).				x	x

Miller, A. E., et al. (2022). "Impact of the COVID-19 pandemic on the psychological health of individuals with mental health conditions: A mixed methods study." Journal of Clinical Psychology 78(4): 710-728.	x								
Milne, S. J., et al. (2020). "Effects of isolation on mood and relationships in pregnant women during the covid-19 pandemic." European Journal of Obstetrics & Gynecology and Reproductive Biology 252: 610-611.	x								
Miranda Olivera, L., et al. (2021). "A perspective on the emotional and cognitive effects of the measures put in place due to COVID-19 in Puerto Rican children." Revista Puertorriquena de Psicologia 32(1): 104-118.	x								
Mistraletti, G., et al. (2020). "How to communicate with families living in complete isolation." BMJ supportive & palliative care. 15.					x				
Mistry, S. K., et al. (2022). "COVID-19 related anxiety and its associated factors: a cross-sectional study on older adults in Bangladesh." BMC Psychiatry 22(1): 737.	x								
Mistry, S. K., et al. (2022). "Stigma toward people with COVID-19 among Bangladeshi older adults." Frontiers in Public Health 10: 982095.	x								
Mitchell, T. O. and L. M. Li (2021). "State-Level Data on Suicide Mortality During COVID-19 Quarantine: Early Evidence of a Disproportionate Impact on Racial Minorities." Psychiatry Research 295.	x								
Mitrokhin, O. V., et al. (2020). Open Public Health Journal 13(1): 734-738.						x			x

Mittal, S. and S. Nagendran (2021). "A study on the effects of stress and hopelessness in isolated COVID-19 patients in relation to severity of infection." European Psychiatry 64(Supplement 1): S665.			x											
Mlouki, I., et al. (2021). "Social stigma among COVID-19 patients after quarantine in Sousse, Tunisia." European Journal of Public Health 31: 382-382.					x									
Modini, M. and L. Vrklevski (2020). "A hotel room on Mars: Quarantine and the psychological view from the virtual front line." Australasian Psychiatry 28(6): 624-626.						x								
Modini, M. and L. Vrklevski (2022). "Virtual psychology support for people in hotel quarantine: predicting surges in demand." Australasian Psychiatry 30(1): 134-135.						x								
Moens, I. S., et al. (2022). "Positive health during the COVID-19 pandemic: a survey among community-dwelling older individuals in the Netherlands." BMC Geriatrics 22(1).		x												
Moghaddam, F. H., et al. (2021). "Prevalence of depression and its related factors during the COVID-19 quarantine among the elderly in Iran." Salmand: Iranian Journal of Ageing 16(1): 140-151.							x				x			
Moghimian, M., et al. (2022). "Exploring the experiences of nurses and physicians infected with COVID-19." Journal of Education and Health Promotion 11(1).							x							x
Mohamed, A. E. and A. M. Yousef (2021). "Depressive, anxiety, and post-traumatic stress symptoms affecting hospitalized and home-isolated COVID-19 patients: a comparative cross-sectional study." Middle East Current Psychiatry-Mecpsych 28(1).	x						x							

Mojarad, F. A., et al. (2021). "Mental health of the people of northern Iran during the quarantine time of 2020 following the coronavirus epidemic." Journal of Education and Health Promotion 10(1).		x				
Moni, A. S. B., et al. (2021). "Psychological distress, fear and coping among Malaysians during the COVID-19 pandemic." PLoS ONE 16(9).				x		x
Montanari Vergallo, G., et al. (2020). "CoViD-19 and psychiatry: can mental illness justify further exceptions to the obligation to stay at home?" Rivista di Psichiatria 55(4): 245-249.				x	x	
Montano, A. H. and J. G. Tovar (2022). "Explanatory model of self-care, emotional regulation and burnout in psychologists in isolation due to COVID-19." Acta Colombiana De Psicologia 25(2): 90-103.				x		x
Monteith, L. L., et al. (2021). "Understanding women's risk for suicide during the COVID-19 pandemic: A call to action." Psychiatry Research Vol 295 2021, ArtID 113621 295.			x			
Monterrosa-Castro, A., et al. (2021). "Perceived Loneliness and Severe Sleep Disorders in Adult Women during the Covid-19 Quarantine: A Cross-Sectional Study in Colombia." Journal of Primary Care & Community Health 12: 21501327211025170.	x					
Montiel Ishino, F. A., et al. (2022). "Substance Use From Social Distancing and Isolation By US Nativity During the Time of Covid-19: Cross-Sectional Study." JMIR public health and surveillance. 18.	x					

Moodley, K., et al. (2020). "Isolation and quarantine in South Africa during COVID-19: Draconian measures or proportional response?" Samj South African Medical Journal 110(6): 456-457.		x				
Moore, E. W. G., et al. (2022). "College Student-athletes' COVID-19 Worry and Psychological Distress Differed by Gender, Race, and Exposure to COVID-19-related Events." Journal of Adolescent Health 70(4): 559-566.			x			x
Moore, R. C., et al. (2021). "Age-Related Differences in Experiences With Social Distancing at the Onset of the COVID-19 Pandemic: A Computational and Content Analytic Investigation of Natural Language From a Social Media Survey." Jmir Human Factors 8(2).	x					
Moore, R., et al. (2021). ""This Pandemic Is Making Me More Anxious about My Welfare and the Welfare of Others:" COVID-19 Stressors and Mental Health." International Journal of Environmental Research & Public Health [Electronic Resource] 18(11): 26.	x					
Morales-Vives, F., et al. (2022). "COmpliance with pandemic COmmands Scale (COCOS): The relationship between compliance with COVID-19 measures and sociodemographic and attitudinal variables." PLoS ONE [Electronic Resource] 17(1): e0262698.				x	x	
Moreira, D. N. and M. P. da Costa (2020). "The impact of the Covid-19 pandemic in the precipitation of intimate partner violence." International Journal of Law and Psychiatry 71.		x				

Moreira, P. S., et al. (2021). "Protective elements of mental health status during the covid-19 outbreak in the portuguese population." International Journal of Environmental Research and Public Health 18(4): 1-11.	x
Moreno-Quispe, L. A., et al. (2021). "Level of physical activity of Peruvian university students during confinement." Journal of Human Sport and Exercise 16: S763-S768.	x
Morrison, S. A., et al. (2020). "Responding to a global pandemic: Republic of Slovenia on maintaining physical activity during self-isolation." Scandinavian Journal of Medicine & Science in Sports 30(8): 1546-1548.	x
Morrison, S. A., et al. (2021). "The Effect of Pandemic Movement Restriction Policies on Children's Physical Fitness, Activity, Screen Time, and Sleep." Frontiers in Public Health 9: 785679.	x
Mosquera-Presedo, M., et al. (2021). "P.0213 Self-perceived mental health and isolation as screening tools in mental health symptoms during covid-19 pandemic." European Neuropsychopharmacology 53(Supplement 1): S155-S156.	x
Mosquera-Presedo, M., et al. (2021). "The effect of covid-19 pandemic in mental health symptoms: isolation and self-perceived mental health." European Neuropsychopharmacology 53: S154-S155.	x

Mostafa, A., et al. (2021). "Validity and Reliability of a COVID-19 Stigma Scale Using Exploratory and Confirmatory Factor Analysis in a Sample of Egyptian Physicians: E16-COVID19-S." International Journal of Environmental Research & Public Health [Electronic Resource] 18(10): 19.	x			
Mscn, S. S. A. and E. G. A. Ma'Ala (2022). "Impact of Preventive Measures upon Children's Emotional Behaviors during COVID-19 Pandemic in Mosul City." Pakistan Journal of Medical and Health Sciences 16(3): 945-947.	x			
Mughal, F., et al. (2021). "Mental health support through primary care during and after covid-19." The BMJ 373 (no pagination).		x		
Muhamad, A. B., et al. (2021). "Retrospective analysis of psychological factors in COVID-19 outbreak among isolated and quarantined agricultural students in a Borneo university." Frontiers in Psychiatry Vol 12 2021, ArtID 558591 12.			x	x
Mukherjee, A., et al. (2021). "COVID-19 pandemic: Mental health and beyond-the Indian perspective." Irish Journal of Psychological Medicine 38(2): 140-144.		x		
Mukherjee, K., et al. (2021). "Impact of COVID-19 pandemic on mental health across different populations." Minerva Psychiatry 62(3): 140-155.		x		
Mukhtar, S. (2020). "Mental Health and Psychosocial Aspects of Coronavirus Outbreak in Pakistan: Psychological Intervention for Public Mental Health Crisis." Asian Journal of Psychiatry 51: 102069.		x		

Multisectoral actions of mental health during the COVID-19 pandemic in Mazandaran province of Iran	x										
Mumbardo-Adam, C., et al. (2021). "How have youth with Autism Spectrum Disorder managed quarantine derived from COVID-19 pandemic? An approach to families perspectives." Research in Developmental Disabilities 110: 103860.	x										
Mumtaz, M. (2021). "COVID-19 and mental health challenges in Pakistan." International Journal of Social Psychiatry 67(3): 303-304.					x						
Munshi, M., et al. (2021). "Impact of isolation during the covid-19 pandemic on the care of older adults with type 1 diabetes: A qualitative study." Diabetes. Conference: 81st Scientific Sessions of the American Diabetes Association, ADA 70(SUPPL 1).					x						
Murali, N., et al. (2020). "Analytical study on the preventive measures followed by public during covid outbreak." International Journal of Pharmaceutical Research 12(Supplementry 2): 635-648.			x								
Murali, N., et al. (2020). "Analytical study on the preventive measures followed by public during covid outbreak." International Journal of Pharmaceutical Research 12(Supplementry 2): 635-648.	x										
Murayama, K., et al. (2022). "The Impact of Gender and Age Differences and Infectious Disease Symptoms on Psychological Distress in Quarantined Asymptomatic or Mildly Ill COVID-19 Patients in Japan." International Journal of Environmental Research & Public Health [Electronic Resource] 19(15): 26.						x					x

Murphy, E., et al. (2022). "The Lived Experience of Older Adults Transferring Between Long-Term Care Facilities During the COVID-19 Pandemic." Journal of Gerontological Nursing 48(1): 29-33.	x			
Muruganandam, P., et al. (2020). "COVID-19 and Severe Mental Illness: Impact on patients and its relation with their awareness about COVID-19." Psychiatry Research 291 (no pagination).	x			
Nabarro, D. and J. Atkinson (2020). "Staying safe while waiting for the vaccine: what we need to do." Qjm 113(10): 717-719.		x		
Nachega, J. B., et al. (2021). "Perspective Contact Tracing and the COVID-19 Response in Africa: Best Practices, Key Challenges, and Lessons Learned from Nigeria, Rwanda, South Africa, and Uganda." American Journal of Tropical Medicine and Hygiene 104(4): 1179-1187.		x		
Naik, S. S., et al. (2020). "Homeless people with mental illness in India and COVID-19." The Lancet Psychiatry 7(8): e51-e52.			x	x
Najafipour, H., et al. (2021). "Effects of quarantine due to the COVID-19 on sleep time, anxiety, and physical activity in adult population: A longitudinal study in Kerman, southeastern Iran." Journal of Kerman University of Medical Sciences 28(3): 219-229.	x			
Najmi, A., et al. (2022). "Easing or tightening control strategies: determination of COVID-19 parameters for an agent-based model." Transportation 49(5): 1265-1293.			x	x
Nakhae, M., et al. (2021). "The experience of family caregivers in treatment of COVID-19 patients: a qualitative study." Neuropsychiatria i Neuropsychologia 16(3-4): 131-137.	x			

Narayana, G., et al. (2020). "Knowledge, perception, and practices towards COVID-19 pandemic among general public of India: A cross-sectional online survey." Current Medicine Research and Practice 10(4): 153-159.		x		
Naser, A. Y., et al. (2021). "The effect of the 2019 coronavirus disease outbreak on social relationships: A cross-sectional study in Jordan." International Journal of Social Psychiatry 67(6): 664-671.		x		
Nash, M., et al. (2022). "It's just that uncertainty that eats away at people: Antarctic expeditioners' lived experiences of COVID-19." PLoS ONE [Electronic Resource] 17(11): e0277676.		x		
Nasir, A. B. M. (2021). "Athletes' mental health & coping strategies during COVID-19 pandemic quarantine training camp." International Journal of Sport and Exercise Psychology 19: S95-S95.		x		
Navarrete, J., et al. (2022). "Connecting to Nature through 360degree Videos during COVID-19 Confinement: A Pilot Study of a Brief Psychological Intervention." Journal of Healthcare Engineering 2022: 4242888.			x	x
Navas, E., et al. (2022). "[Mental health consequences of isolation of patients with COVID-19.]." Revista Espanola De Salud Publica 96: 21.	x		x	
Nazari, E., et al. (2020). "Home quarantine is a useful strategy to prevent the coronavirus outbreak: Identifying the reasons for non-compliance in some Iranians." Informatics in Medicine Unlocked 21 (no pagination).		x		

Ndejjo, R., et al. (2021). "Experiences of persons in COVID-19 institutional quarantine in Uganda: a qualitative study." BMC Public Health 21(1): 482.				x		x
Nebhinani, N. (2021). "Steps to mitigate the immediate and long-term consequences of covid-19 pandemic on children and adolescents." Journal of Indian Association for Child and Adolescent Mental Health 17(4): 1-5.			x			
Nelson, B. W., et al. (2020). "Rapid assessment of psychological and epidemiological correlates of COVID-19 concern, financial strain, and health-related behavior change in a large online sample." PLoS ONE Vol 15(11), 2020, ArtID e0241990 15(11).	x					
Nelson, B. W., et al. (2020). PLoS ONE 15(11 November) (no pagination).	x					
Nese, M., et al. (2022). "Delay discounting of compliance with containment measures during the COVID-19 outbreak: a survey of the Italian population." Journal of Public Health (Germany) 30(2): 503-511.	x					
Ng, T. C., et al. (2021). "Comparison of Estimated Effectiveness of Case-Based and Population-Based Interventions on COVID-19 Containment in Taiwan." JAMA Internal Medicine 181(7): 913-921.				x	x	
Nguyen, A., et al. (2021). "COVID-19, MENTAL HEALTH STATUS, and ADHERENCE to PUBLIC HEALTH GUIDELINES among the ELDERLY POPULATION in UNITED STATES." Journal of Investigative Medicine 70: 267.		x				

Nguyen, H. B., et al. "Post-traumatic stress disorder, anxiety, depression and related factors among COVID-19 patients during the fourth wave of the pandemic in Vietnam." International Health.		x	x
Nguyen, N. P. T., et al. (2020). "Preventive behavior of Vietnamese people in response to the COVID-19 pandemic." PLoS ONE [Electronic Resource] 15(9): e0238830.	x		
Nilsson, G., et al. (2021). "Old Overnight: Experiences of Age-Based Recommendations in Response to the COVID-19 Pandemic in Sweden." Journal of Aging & Social Policy 33(4-5): 359-379.	x		
Nina, Y., et al. (2021). "Conspiracy Belief and Behavior in the COVID-19 Pandemic. How Belief in Conspiracy Theory Relates to Adherence to Quarantine Restrictions (Wearing Protective Equipment, Isolation, Hygiene) and Influences Antisocial Behavior such as Aggression and Selfishness, as well as Prosocial Behavior such as Help and Altruism." Brain-Broad Research in Artificial Intelligence and Neuroscience 12(2): 202-221.	x		
Nirubama, K., et al. (2020). "Awareness of protective measure taken by government and self-protective measure during pandemic-a questionnaire survey." International Journal of Pharmaceutical Research 12(Supplementry 2): 2205-2217.	x		
Nisa, C. F., et al. (2021). "Lives versus Livelihoods? Perceived economic risk has a stronger association with support for COVID-19 preventive measures than perceived health risk." Scientific Reports 11(1): 9669.	x		

Nishitani, K., et al. (2021). "Self-quarantine programme and pre-operative SARS-CoV-2 PCR screening for orthopaedic elective surgery: experience from Japan." International Orthopaedics 45(5): 1147-1153.	x		
Niu, Z., et al. (2020). "Chinese Public's engagement in preventive and intervening health behaviors during the early breakout of COVID-19: Cross-sectional study." Journal of Medical Internet Research 22(8) (no pagination).	x		
Nkire, N., et al. (2021). "COVID-19 Pandemic: Demographic Predictors of Self-Isolation or Self-Quarantine and Impact of Isolation and Quarantine on Perceived Stress, Anxiety, and Depression." Frontiers in Psychiatry 12 (no pagination).		x	x
Nkodila, A. N., et al. (2021). "Psychological Consequence of the Corona Virus Disease Pandemic in Kinshasa, Democratic Republic of the Congo. A Population-Based Cross-Sectional Survey." Risk Management and Healthcare Policy 14: 2163-2170.	x		
Nofal, A. M., et al. (2020). "Who complies with COVID-19 transmission mitigation behavioral guidelines?" PLoS ONE 15(10 October) (no pagination).	x		
Noman, A. H. M., et al. (2021). "The detrimental effects of the COVID-19 pandemic on domestic violence against women." Journal of Psychiatric Research 134: 111-112.	x		
Nurunnabi, M., et al. (2020). "Mental health and well-being during the COVID-19 pandemic in higher education: Evidence from G20 countries." Journal of Public Health Research 9: 60-68.	x		

Nwaeze, O., et al. (2021). "Factors affecting willingness to comply with public health measures during the pandemic among sub-Sahara Africans." African Health Sciences 21(4): 1629-1639.		x		
Ochnik, D., et al. (2022). "Longitudinal Predictors of Coronavirus-Related PTSD among Young Adults from Poland, Germany, Slovenia, and Israel." International Journal of Environmental Research & Public Health [Electronic Resource] 19(12): 12.		x		
Oginni, O. A., et al. (2021). "Depressive and anxiety symptoms and COVID-19-related factors among men and women in Nigeria." PLoS ONE [Electronic Resource] 16(8): e0256690.	x		x	
Ohlmeier, S., et al. (2022). "Having a Break or Being Imprisoned: Influence of Subjective Interpretations of Quarantine and Isolation on Boredom." International Journal of Environmental Research & Public Health [Electronic Resource] 19(4): 15.			x	x
Okhrimenko, I. and N. Lyhun (2020). "Stress Prevention and Management during the Quarantine." Brain-Broad Research in Artificial Intelligence and Neuroscience 11(2): 157-164.		x		
Okobi, S., et al. (2022). "Evaluation of Stigma Related to Perceived Risk for Coronavirus-19 Transmission Relative to the Other Stigmatized Conditions Opioid Use and Depression." Frontiers in Psychiatry 13.			x	x
Okonofua, F. E., et al. (2022). PLoS ONE 17(8 August) (no pagination).		x		

Olani, A. B., et al. (2022). "Exploring experiences of quarantined people during the early phase of COVID-19 outbreak in Southern Nations Nationalities and Peoples' Region of Ethiopia: A qualitative study." PLoS ONE [Electronic Resource] 17(9): e0275248.					x		x	
Oldham, M. A., et al. (2021). "Mental Health, COVID-19, and the Invisible Pandemic on the Horizon." Mayo Clinic Proceedings 96(2): 287-290.				x				
Olkhovaya, T. A., et al. (2020). "Media clips on social fear to regulate individual behavior in temporary involuntary isolation (quarantine)." Cuestiones Politicas 38(66): 73-84.			x					
Oloniniyi, I. O., et al. "Prevalence and Pattern of Intimate Partner Violence During COVID-19 Pandemic Among Nigerian Adults." Psychological Trauma-Theory Research Practice and Policy.					x	x		
Omaka-Amari, L. N., et al. (2020). "Coronavirus (COVID-19) Pandemic in Nigeria: Preventive and Control Challenges within the First Two Months of Outbreak." African Journal of Reproductive Health 24(s1): 87-97.				x				
Omaleki, V., et al. ""It's hard for everyone" systemic barriers to home confinement to prevent community spread of COVID-19." Translational Behavioral Medicine.					x			x
Omiya, Y. and S. Tokuno (2020). "How much of an impact did COVID-19 self-isolation measures have on mental health?" Asian Journal of Psychiatry 54.		x						
Opakunle, T., et al. (2022). "Associated Psychological Factors of Viral Load among Self-Isolating Nigerian COVID-19 Patients." West African Journal of Medicine 39(6): 588-594.	x				x			

Orsini, A., et al. (2021). "Post-traumatic stress, anxiety, and depressive symptoms in caregivers of children tested for COVID-19 in the acute phase of the Italian outbreak." Journal of Psychiatric Research 135: 256-263.		x		x
Overall, N. C., et al. (2022). "Partners' Attachment Insecurity and Stress Predict Poorer Relationship Functioning During COVID-19 Quarantines." Social Psychological and Personality Science 13(1): 285-298.	x			
Ozcan, B. A. and B. Yeslikaya (2021). "Adverse Effect of Emotional Eating Developed During the COVID-19 Pandemic on Healthy Nutrition, a Vicious Circle: A cross-sectional descriptive study." Revista Espanola De Nutricion Humana Y Dietetica 25.	x			
Ozdogan, B., et al. (2022). "The Relationship of Covid-19 Related Anxiety with Positive and Negative Emotions of Individuals." Cyprus Turkish Journal of Psychiatry and Psychology 4(3): 231-241.	x			
Ozlu, I., et al. (2021). "The compliance of emergency healthcare personnel with isolation precautions during the COVID-19 pandemic: A cross-sectional questionnaire study." International Journal of Clinical Practice 75(10): e14492.		x	x	
Padidar, S., et al. (2021). "Assessment of early COVID-19 compliance to and challenges with public health and social prevention measures in the Kingdom of Eswatini, using an online survey." PLoS ONE [Electronic Resource] 16(6): e0253954.	x			

Padrosa, E. and M. Bolibar "Disentangling youth non-compliance with COVID-19 restrictions from gender, socioeconomic vulnerability and poor mental health: lessons from the first wave in Catalonia." Journal of Youth Studies.	x			
Pai, N., et al. (2022). "EMOTIONAL IMPACT AND RESILIENCE DUE TO ISOLATION (EIRI) DURING COVID-19 AMONG MEDICAL STUDENTS AND STAFF: A WEB-BASED SURVEY IN INDIA." Australian and New Zealand Journal of Psychiatry 56(1_SUPPL): 208-208.	x			
Paludo, A. C., et al. (2021). "Female students are the most psychologically affected by the COVID-19 outbreak: a case study in an academic community in Brazil." Revista Da Associacao Medica Brasileira 67(5): 741-746.	x			
Pandey, K. (2020). "A comparative study of behavioral patterns of sportspersons and non-sports persons quarantined during COVID-19: A case study of Ghaziabad city." Journal of Family Medicine and Primary Care 9(10): 5366-5371.		x		
Pang, N. T. P., et al. (2021). "Relationships between psychopathology, psychological process variables, and sociodemographic variables and comparison of quarantined and non-quarantined groups of malaysian university students in the covid-19 pandemic." International Journal of Environmental Research and Public Health 18(18) (no pagination).			x	x
Papandreou, C., et al. (2020). "Comparing eating behaviours, and symptoms of depression and anxiety between Spain and Greece during the COVID-19 outbreak: Cross-sectional analysis of two different confinement strategies." European Eating Disorders Review 28(6): 836-846.	x			

Park, C. L., et al. (2020). "Americans' COVID-19 Stress, Coping, and Adherence to CDC Guidelines." Journal of General Internal Medicine 35(8): 2296-2303.		x	x	
Park, H., et al. (2022). "Stress Experience of COVID-19 Patients as Reported by Psychological Supporters in South Korea: A Qualitative Study." Frontiers in Psychiatry 13.		x		x
Park, M. B., et al. (2021). "Effect of the Period From COVID-19 Symptom Onset to Confirmation on Disease Duration: Quantitative Analysis of Publicly Available Patient Data." Journal of Medical Internet Research 23(9).		x	x	
Parrello, S., et al. (2021). "Adolescents' dreams under COVID-19 isolation." International Journal of Dream Research 14(1): 10-20.	x			
Parsons Leigh, J., et al. (2020). "A national cross-sectional survey of public perceptions of the COVID-19 pandemic: Self-reported beliefs, knowledge, and behaviors." PLoS ONE [Electronic Resource] 15(10): e0241259.	x			
Pathare, S., et al. (2020). "Analysis of news media reports of suicides and attempted suicides during the COVID-19 lockdown in India." International Journal of Mental Health Systems 14(1) (no pagination).		x	x	
Pathmanathan, M. D., et al. (2021). "A Safe Home Quarantine Digital Solution for COVID-19: A Proof-of-Concept Study." Medical Journal of Malaysia 76(SUPPL 5): 42.		x	x	
Patrick, M. E., et al. (2022). "Using Substances to Cope With the COVID-19 Pandemic: U.S. National Data at Age 19 Years." Journal of Adolescent Health 70(2): 340-344.	x			

Patwary, M. M., et al. (2022). "Knowledge, Attitudes, and Practices Toward Coronavirus and Associated Anxiety Symptoms Among University Students: A Cross-Sectional Study During the Early Stages of the COVID-19 Pandemic in Bangladesh." Frontiers in Psychiatry 13.				x		x
Paulauskaite, L., et al. (2021). "My son can't socially distance or wear a mask: How families of preschool children with severe developmental delays and challenging behavior experienced the COVID-19 pandemic." Journal of Mental Health Research in Intellectual Disabilities 14(2): 225-236.		x				
Pawlowski, M., et al. (2022). "Psychopathological Symptoms Among Chronically Ill Patients During SARS-CoV-2 Pandemic in Poland." Psychology Research and Behavior Management 15: 2659-2669.		x				
Paz, C., et al. (2020). "Behavioral and sociodemographic predictors of anxiety and depression in patients under epidemiological surveillance for COVID-19 in Ecuador." PLoS ONE [Electronic Resource] 15(9): e0240008.	x			x		
Peitl, V., et al. (2020). "Mental health issues and psychological crisis interventions during the COVID-19 pandemic and earthquakes in Croatia." Archives of Psychiatry Research: An International Journal of Psychiatry and Related Sciences 56(2): 193-198.			x			
Pel-Littel, R. E., et al. (2022). "Lessons Learned From the COVID-19 Pandemic as Experienced by Older Adults Treated for COVID-19." Gerontology and Geriatric Medicine 8.				x	x	

Peng, M., et al. (2020). "Prevalence, risk factors and clinical correlates of depression in quarantined population during the COVID-19 outbreak." Journal of Affective Disorders 275: 119-124.					x							
Pereira, A. T., et al. (2022). "Adherence Scale to the Recommendations for Mental Health during the COVID-19 pandemic from the Portuguese General Directorate of Health (ASR-MHCOVID19) - Development and validation." European Psychiatry 65(Supplement 1): S265.				x								
Perez-Siguas R, Matta-Solis E, Matta-Solis H, Remuzgo-Artezano A, Matta-Zamudio L, Yauri-Machaca M. Level of Depression in Positive Patients for COVID-19 in the Puente Piedra District of North Lima, 2020. International Journal of Advanced Computer Science and Applications. 2020;11(10):546-51.							x					x
Perez-Siguas R, Seminario-Unzueta R, Matta-Solis H, Yauri-Machaca M, Matta-Solis E. Problematic Use of Mobile Phones during the COVID-19 Pandemic in Peruvian University Students, 2020. International Journal of Advanced Computer Science and Applications. 2020;11(12):445-50.		x					x					
Pericas JM, Cucchiari D, Torrallardona-Murphy O, Calvo J, Serralabos J, Alves E, et al. Hospital at home for the management of COVID-19: preliminary experience with 63 patients. Infection. 2021;49(2):327-32.							x			x		
Petrocchi et al. (2021)	x						x					

Petzold, M.B., Bendau, A., Plag, J., Pyrkosch, L., Mascarell Maricic, L., Betzler, F., Rogoll, J., Grosse, J., Strohle, A., 2020. Risk, resilience, psychological distress, and anxiety at the beginning of the COVID-19 pandemic in Germany. Brain and Behavior. 10(9) (no pagination), e01745.		x	
Pham-Scottez, A., Silva, J., Barruel, D., Dauriac-Le Masson, V., Yon, L., Trebalag, A.K., Gourevitch, R., 2020. Patient flow in the largest French psychiatric emergency centre in the context of the COVID-19 pandemic. Psychiatry Research. 291, 113205. https://doi.org/10.1016/j.psychres.2020.113205.		x	
Pheh, K. S., Tan, H. C., & Tan, C. S. (2020). Ultra-Brief Online Mindfulness-Based Intervention Effects on Mental Health During the Coronavirus Disease Outbreak in Malaysia: A Randomized Controlled Trial. Makara Hubs-Asia, 24(2), 118-128. https://doi.org/10.7454/hubs.asia.2140920	x		x
Pigaiani, Y., Zoccante, L., Zocca, A., Arzenton, A., Menegolli, M., Fadel, S., Ruggeri, M., Colizzi, M., 2020. Adolescent Lifestyle Behaviors, Coping Strategies and Subjective Wellbeing during the COVID-19 Pandemic: An Online Student Survey. Healthcare. 8, 472. https://doi.org/10.3390/healthcare8040472.		x	
Pineda-Garcia, G., Serrano-Medina, A., Ochoa-Ruiz, E., Martinez, A.L., 2021. Body Image, Anxiety, and Bulimic Behavior during Confinement Due to COVID-19 in Mexico. Healthcare. 9, 1435. https://doi.org/10.3390/healthcare9111435.		x	

Pinheiro, C., Thuruthiyath, L.R., Philip, S., Viswabhadran, A.M., Sivadasan, A.M., 2022. Quarantine of Travellers during the Initial Phase of the COVID-19 Pandemic- Experience from a Rural Setting in Kerala, India. Journal of Clinical and Diagnostic Research. 16(9), LC27-LC31.	x												x
Plesea-Condratovici, C., Gatej, E.R., Rizeanu, S., Plesea-Condratovici, A., 2022. Anxiety in Home-Quarantined Patients with COVID-19. Brain-Broad Research in Artificial Intelligence and Neuroscience. 13, 347-360. https://doi.org/10.18662/brain/13.1/288.		x						x					
Pogorilska, N.I., Synelnykov, R.Y., Palamar, B.I., Tukaiev, S.V., Nezhyva, L.L., 2021. Features of Psychological Experiences in Severe Quarantine during the Covid-19 Pandemic: The Role of Tolerance for Uncertainty. Wiadomosci Lekarskie. 74, 1312-1316.			x										
Pokryszko-Dragan, A., Chojdak-Lukasiewicz, J., Gruszka, E., Pawlowski, M., Pawlowski, T., Rudkowska-Mytych, A., Rymaszewska, J., Budrewicz, S., 2021. Burden of COVID-19 pandemic perceived by polish patients with multiple sclerosis. Journal of Clinical Medicine. 10(18) (no pagination), 4215.				x									
Pollak, Y., Dayan, H., Shoham, R., Berger, I., 2020. Predictors of non-adherence to public health instructions during the COVID-19 pandemic. Psychiatry and Clinical Neurosciences. 74, 602-604.								x			x		

Pollak, Y., Shoham, R., Dayan, H., Gabrieli-Seri, O., Berger, I., 2022. Background and concurrent factors predicting non-adherence to public health preventive measures during the chronic phase of the COVID-19 pandemic. Journal of Public Health. 44, E117-E125, fdab214. https://doi.org/10.1093/pubmed/fdab214.		x		x
Pollak, Y., Shoham, R., Dayan, H., Gabrieli-Seri, O., Berger, I., 2022. Symptoms of ADHD Predict Lower Adaptation to the COVID-19 Outbreak: Financial Decline, Low Adherence to Preventive Measures, Psychological Distress, and Illness-Related Negative Perceptions. Journal of Attention Disorders. 26, 735-746.		x		x
Pollak, Y., Shoham, R., Dayan, H., Seri, O. G., & Berger, I. (2021). ADHD symptoms predict lower adaptation to the COVID-19 outbreak: financial decline, low adherence to preventive measures, psychological distress, and illness-related negative perceptions.	x			
Polskaya, N.A., Razvaliaeva, A.Y., 2020. Interpersonal Sensitivity in the Period of Self-Isolation and its Role in the Choice of Social Distancing Measures. Psikhologicheskaya Nauka I Obrazovanie-Psychological Science and Education. 25, 63-76. https://doi.org/10.17759/pse.2020250606.	x			
Porteny, T., Corlin, L., Allen, J.D., Monahan, K., Acevedo, A., Stopka, T.J., Levine, P., Ladin, K., 2022. Associations among political voting preference, high-risk health status, and preventative behaviors for COVID-19. BMC Public Health. 22, 225.			x	x

Poyraz Findik, O.T., Ceri, V., Perdahli Fis, N., 2022. Factors associated with anxiety and post-traumatic stress symptomatology during the COVID-19 pandemic in Turkey: A comparison of youths and adults. Marmara Medical Journal. 35(2), 202-210.	x		
Prachthauser, M., Cassisi, J.E., Le, T.-A., Nicasio, A.V., 2020. The Social Distance Scale (v1): A screening instrument to assess patient adherence to prevention strategies during pandemics. International Journal of Environmental Research and Public Health Vol 17(21), 2020, ArtID 8158. 17.		x	x
Prakash, J., Dangi, A., Chaterjee, K., Yadav, P., Srivastava, K., Chauhan, V.S., 2021. Assessment of depression, anxiety and stress in COVID-19 infected individuals and their families. Medical Journal Armed Forces India. 77(Supplement 2), S424-S429.	x		
Prasetyo, Y.T., Castillo, A.M., Salonga, L.J., Sia, J.A., Seneta, J.A., 2020. Factors affecting perceived effectiveness of COVID-19 prevention measures among Filipinos during Enhanced Community Quarantine in Luzon, Philippines: Integrating Protection Motivation Theory and extended Theory of Planned Behavior. International Journal of Infectious Diseases. 99, 312-323. https://doi.org/10.1016/j.ijid.2020.07.074.		x	x
Prete, G., Fontanesi, L., Porcelli, P., Tommasi, L., 2020. The psychological impact of COVID-19 in Italy: Worry leads to protective behavior, but at the cost of anxiety. Frontiers in Psychology Vol 11 2020, ArtID 566659. 11.		x	x

Proserpio, P., Zambrelli, E., Lanza, A., Dominese, A., Di Giacomo, R., Quintas, R., Tramacere, I., Rubino, A., Turner, K., Colosio, C., Cattaneo, F., Canevini, M.P., D'Agostino, A., Agostoni, E.C., Didato, G., 2022. Sleep disorders and mental health in hospital workers during the COVID-19 pandemic: a cross-sectional multicenter study in Northern Italy. Neurological Sciences. 43, 2241-2251.	x
Psychological impact of mandatory covid-19 quarantine on small business owners and self-employed in china	x
Puccinelli, P.J., Costa, T.S., Seffrin, A., de Lira, C.A.B., Vancini, R.L., Knechtle, B., Nikolaidis, P.T., Andrade, M.S., 2021. Physical Activity Levels and Mental Health during the COVID-19 Pandemic: Preliminary Results of a Comparative Study between Convenience Samples from Brazil and Switzerland. Medicina. 57, 08.	x
Puerta, J.G., Malagon, M.C.G., Gijon, M.K., Simon, E.J.L., 2022. Levels of Stress, Anxiety, and Depression in University Students from Spain and Costa Rica during Periods of Confinement and Virtual Learning. Education Sciences. 12, 660. https://doi.org/10.3390/educsci12100660.	x
Qiu, J.Y., Shen, B., Zhao, M., Wang, Z., Xie, B., Xu, Y.F., 2020. A nationwide survey of psychological distress among Chinese people in the COVID-19 epidemic: implications and policy recommendations. General Psychiatry. 33, e100213. https://doi.org/10.1136/gpsych-2020-100213.	x

Quintiliani, L., Sisto, A., Vicinanza, F., Curcio, G., Tambone, V., 2022. Resilience and psychological impact on Italian university students during COVID-19 pandemic. Distance learning and health. Psychology Health & Medicine. 27, 69-80.	x				
Qureshi, M.F., Kango, R.A., Zaki, N., Shaikh, F.K., Ieee, 2021. Activity Monitoring of the Potential COVID'19 Individuals in Quarantine Facility, 47th Annual Conference of the IEEE-Industrial-Electronics-Society (IECON), Electr Network.		x	x		
Rachubinska, K., Cybulska, A.M., Solek-Pastuszka, J., Panczyk, M., Stanislawska, M., Ustianowski, P., Grochans, E., 2022. Assessment of Psychosocial Functioning of Polish Nurses during COVID-19 Pandemic. International Journal of Environmental Research & Public Health [Electronic Resource]. 19, 27.		x			x
Rafique, N., Al Tufaif, F., Alhammali, W., Alalwan, R., Aljaroudi, A., AlFaraj, F., Latif, R., Al-Asoom, L.I., Alsunni, A.A., Al Ghamdi, K.S., Salem, A.M., Yar, T., 2022. The Psychological Impact of COVID-19 on Residents of Saudi Arabia. Psychology Research and Behavior Management. 15, 1221-1234. https://doi.org/10.2147/prbm.S360772.		x			x
Rahman, M.A., Rahman, S., Wazib, A., Arafat, S.M.Y., Chowdhury, Z.Z., Uddin, B.M.M., Rahman, M.M., Moni, A.S.B., Alif, S.M., Sultana, F., Salehin, M., Islam, S.M.S., Cross, W., Bahar, T., 2021. COVID-19 Related Psychological Distress, Fear and Coping: Identification of High-Risk Groups in Bangladesh. Frontiers in Psychiatry. 12, 718654. https://doi.org/10.3389/fpsyt.2021.718654.		x		x	

Rahman, M.H., Banik, G., Ahmed, A., El Arifeen, S., Hossain, A.T., Hasan, M.A., Rahman, A.E., 2021. Anxiety and depressive symptoms among COVID-19 patients admitted to three isolation facilities in Bangladesh. Health Psychology Open. 8, 20551029211046106. https://doi.org/10.1177/20551029211046106.			x							
Rahman, M.M., Rahaman, M.S., 2022. Psychological status of mass people in the capital city of Bangladesh during COVID-19: Do home quarantine challenges matter? Journal of Community Psychology. 50, 2090-2103.		x								
Rahman, N.A., Abdullah, M.S., Asli, R., Chong, P.L., Mani, B.I., Chong, V.H., 2022. Challenges during the second wave of COVID-19 in Brunei Darussalam: National Isolation Centre to National COVID-19 Hospital. Western Pacific Surveillance and Response. 13. https://doi.org/10.5365/wpsar.2022.13.3.913.							x			x
Rainero, I., Bruni, A.C., Marra, C., Cagnin, A., Bonanni, L., Cupidi, C., Lagana, V., Rubino, E., Vacca, A., Di Lorenzo, R., Provero, P., Isella, V., Vanacore, N., Agosta, F., Appollonio, I., Caffarra, P., Busse, C., Sambati, R., Quaranta, D., Guglielmi, V., Logroscino, G., Filippi, M., Tedeschi, G., Ferrarese, C., 2021. The impact of COVID-19 quarantine on patients with dementia and family caregivers: A nation-wide survey. Frontiers in Aging Neuroscience Vol 12 2021, ArtID 625781. 12.		x								
Rajagopalan, A., Kuppusamy, M., Gandhi, T.S., 2022. Meditation Alleviates Psychological Distress and Sleep Quality in COVID-19 Patients during Home Isolation. Journal of Young Pharmacists. 14, 441-443. https://doi.org/10.5530/jyp.2022.14.89.	x						x			

Rajkumar, R.P., 2020. Suicides related to the COVID-19 outbreak in India: A pilot study of media reports. Asian Journal of Psychiatry. 53 (no pagination), 102196.	x		
Ramadhana, M.R., 2020. dataset for emotional reactions and family resilience during COVID-19 isolation period among Indonesian families. Data in Brief. 31, 105946. https://doi.org/10.1016/j.dib.2020.105946.	x		
Ramezani et al. (2021)	x		
Ramezani, N., Ashtari, F., Bastami, E.A., Ghaderi, K., Hosseini, S.M., Naeini, M.K., Rajabi, F., Adibi, I., 2021. Fear and anxiety in patients with multiple sclerosis during COVID-19 pandemic; report of an Iranian population. Multiple Sclerosis and Related Disorders. 50, 102798.	x		
Ramirez-Cervantes et al. (2020)	x		
Ramirez-Coronel et al. (2020): Anxiety and depression in the face of COVID-19 confinement in adult women in Azogues.	x		
Ramli, M., Mukramati, M., Ikhwan, M., Hafnani, H., 2022. Community behavior for mathematical model of coronavirus disease 2019 (COVID-19). Global Journal of Environmental Science and Management-Gjesm. 8, 151-168. https://doi.org/10.22034/gjesm.2022.02.01.		x	x
Ramos-Padilla, P., Villavicencio-Barriga, V.D., Cardenas-Quintana, H., Abril-Merizalde, L., Solis-Manzano, A., Carpio-Arias, T.V., 2021. Eating Habits and Sleep Quality during the COVID-19 Pandemic in Adult Population of Ecuador. International Journal of Environmental Research & Public Health [Electronic Resource]. 18, 31.	x		

Rasskazova, E. I., & Tkhostov, A. S. (2021). Crisis of trust and decision-making in a pandemic: the role of the social context in the choice of protective actions. Voprosy Psikhologii(6), 78-+. <Go to ISI>://WOS:000843186500008		x		
Rasskazova, E.I., 2021. [Psychological factors of sleep and daytime complaints during the COVID-19 Lockdown: the role of anxiety, well-being, autonomy, and coping]. Zhurnal Nevrologii i Psikhiatrii Imeni S.S. Korsakova. 121, 24-30.	x			
Rasskazova, E.I., Leontiev, D.A., Lebedeva, A.A., 2020. Pandemic as a challenge to subjective well-being: Anxiety and coping. Konsul'tativnaia psikhologiia i psikhoterapiia. 28, 90-108.	x			
Rastogi, T., Awasthi, S., Khare, R., Prasad, M., Sami, G., Verma, V.K., 2022. Clinical Epidemiology and Global Health. 13 (no pagination), 100927.			x	x
Ratschen, E., Shoesmith, E., Shahab, L., Silva, K., Kale, D., Toner, P., Reeve, C., Mills, D.S., 2020. Human-animal relationships and interactions during the Covid-19 lockdown phase in the UK: Investigating links with mental health and loneliness. PLoS ONE. 15(9 September) (no pagination), e0239397.	x			
Ravindran, R.M., Krishnan, R.A., Kiran, P.S., Mohan, B., Shinu, K.S., 2021. Psychosocial Intervention Model of Kerala, India During Pandemic COVID-19: "Ottakkalla Oppamundu (You're not alone, we're with you)". International Journal of Health Services. 51, 436-445, 00207314211019240. https://doi.org/10.1177/00207314211019240.	x			

Reche-Garcia, C., Morante, J.J.H., Santana, J.T.T., Cisneros, C.A.G., Romero, J.R., Montero, F.J.O., 2022. Psychological well-being of Mexican adolescent athletes confined by the COVID-19 pandemic. Cultura Ciencia Y Deporte. 17, 7-13. https://doi.org/10.12800/ccd.v17i52.1681.	x			
Recio-Vivas, A.M., Font-Jimenez, I., Mansilla-Dominguez, J.M., Belzunegui-Eraso, A., Diaz-Perez, D., Lorenzo-Allegue, L., Pena-Otero, D., 2022. Fear and Attitude towards SARS-CoV-2 (COVID-19) Infection in Spanish Population during the Period of Confinement. International Journal of Environmental Research & Public Health [Electronic Resource]. 19, 12.	x			
Regehr, C., Goel, V., De Prophetis, E., Jamil, M., Mertz, D., Rosella, L.C., Bulir, D., Smieja, M., 2021. Investigating the impact of quarantine on mental health: Insights from the COVID-19 international border surveillance study in Canada. BJPsych Open Vol 7 2021, ArtID e143. 7.		x		x
Reguera-Garcia, M.M., Liebana-Presa, C., Alvarez-Barrio, L., Alves Gomes, L., Fernandez-Martinez, E., 2020. Physical Activity, Resilience, Sense of Coherence and Coping in People with Multiple Sclerosis in the Situation Derived from COVID-19. International Journal of Environmental Research & Public Health [Electronic Resource]. 17, 06.	x			
Reicher, S., Stott, C., 2020. On order and disorder during the COVID-19 pandemic. British Journal of Social Psychology. 59, 694-702.		x	x	

Reinel, M., Quevedo, Y., Hernandez, C., Mino, V., Rojas, A., 2022. "emotional drinking" during quarantine by covid-19 in chile: The role of depressive symptoms on problematic alcohol consumption. Journal of Substance Use. No Pagination Specified.	x								
Reinhardt, M., Findley, M.B., Countryman, R.A., 2021. Policy liberalism and source of news predict pandemic-related health behaviors and trust in the scientific community. PLoS ONE [Electronic Resource]. 16, e0252670.						x			x
Reshetnikov, A.V., Prisyazhnaya, N.V., Pavlov, S.V., Vyatkina, N.Y., 2020. PERCEPTION OF THE COVID-19 PANDEMIC BY MOSCOW RESIDENTS. Sotsiologicheskie Issledovaniya. 138-143. https://doi.org/10.31857/s013216250009481-2.	x								
Reynolds, C.M., Purdy, J., Rodriguez, L., McAvoy, H., 2021. Factors associated with changes in consumption among smokers and alcohol drinkers during the COVID-19 'lockdown' period. European Journal of Public Health. 31, 1084-1089.						x			x
Riaz, S., Tariq, S., Mirza, U.T., Khan, M.T., Chaudhary, S., Arshad, M., 2021. The feelings & perceptions of a medical doctor after recovery from corona infection. Pakistan Journal of Medical and Health Sciences. 15(5), 939-942.		x							
Ribeiro et al. (2021): Mental health risk factors during the first wave of the COVID-19 pandemic	x								

Ribeiro, F.S., Santos, F.H., Anunciacao, L., Barrozo, L., Landeira-Fernandez, J., Leist, A.K., 2021. Exploring the Frequency of Anxiety and Depression Symptoms in a Brazilian Sample during the COVID-19 Outbreak. International Journal of Environmental Research & Public Health [Electronic Resource]. 18, 01.)	x			
Riccardi, A., Gemignani, J., Fernandez-Navarro, F., Heffernan, A., 2021. Optimisation of Non-Pharmaceutical Measures in COVID-19 Growth via Neural Networks. Ieee Transactions on Emerging Topics in Computational Intelligence. 5, 79-91. https://doi.org/10.1109/tetci.2020.3046012.		x	x	
Riggio, G., Borrelli, C., Piotti, P., Grondona, A., Gazzano, A., Di Iacovo, F.P., Fatjo, J., Bowen, J.E., Mota-Rojas, D., Pirrone, F., Mariti, C., 2022. Cat-Owner Relationship and Cat Behaviour: Effects of the COVID-19 Confinement and Implications for Feline Management. Veterinary Sciences. 9(7) (no pagination), 369.	x			
Riiser, K., Helseth, S., Haraldstad, K., Torbjornsen, A., Richardsen, K.R., 2020. Adolescents' health literacy, health protective measures, and health-related quality of life during the Covid-19 pandemic. PLoS ONE. 15(8 august) (no pagination), e0238161.		x		x
Rios-de-Deus, M.P., Rodicio-Garcia, M.L., Rego-Agraso, L., Mosquera-Gonzalez, M.J., Losa-Iglesias, M.E., Becerro-de-Bengoa-Vallejo, R., Lopez-Lopez, D., 2022. Student Perceptions of the Resilience in a Confinement Due to COVID-19 in University of A Coruna: A Qualitative Research. Behavioral Sciences. 12, 294. https://doi.org/10.3390/bs12080294.	x			

Ripon, R.K., El-Sabban, F., Sikder, T., Hossain, S., Mim, S.S., Ahmed, H.U., Mehta, N., 2021. Psychological and nutritional effects on a COVID-19-quarantined population in Bangladesh. Journal of Human Behavior in the Social Environment. 31, 271-282.			x												
Ripon, R.K., Mim, S.S., Puente, A.E., Hossain, S., Babor, M.M.H., Sohan, S.A., Islam, N., 2020. COVID-19: psychological effects on a COVID-19 quarantined population in Bangladesh. Heliyon. 6, e05481. https://doi.org/10.1016/j.heliyon.2020.e05481.	x	x													
Risal, A., Shikhrakar, S., Mishra, S., Kunwar, D., Karki, E., Shrestha, B., Khadka, S., Holen, A., 2020. Anxiety and depression during COVID-19 pandemic among medical students in Nepal. Kathmandu University Medical Journal. 18(72), 333-339.			x												
Ritish, D., Dinakaran, D., Chander, R., Murugesan, M., Ibrahim, F.A., Parthasarathy, R., Pandey, P.K., Sharma, M.K., Pandian, D., Manjunatha, N., Reddi, S.K., Moirangthem, S., Kumar, C.N., Suresh, B.M., Gangadhar, B.N., 2020. Mental health concerns in quarantined international air passengers during COVID-19 pandemic - An experiential account. Asian Journal of Psychiatry. 53, 102364.								x					x		
Rivas, D.R.Z., Jaldin, M.L.L., Canaviri, B.N., Escalante, L.F.P., Fernandez, A.M.C.A., Ticona, J.P.A., 2021. Social media exposure, risk perception, preventive behaviors and attitudes during the COVID-19 epidemic in la Paz, Bolivia: A cross sectional study. PLoS ONE. 16(1 January) (no pagination), e0245859.									x		x				

Rivera, J., Castrejon, I., Vallejo-Slocker, L., Offenbacher, M., Molina-Collada, J., Trives, L., Lopez, K., Caballero, L., Hirsch, J.K., Toussaint, L., Nieto, J.C., Alvaro-Gracia, J.M., Vallejo, M.A., 2021. Clinical impact of confinement due to the COVID-19 pandemic on patients with fibromyalgia: a cohort study. Clinical & Experimental Rheumatology. 39 Suppl 130, 78-81.		x	
Rivillas, J. C., Murad, R., Rivera, D., Calderon, M., Sanchez, M., Castano, L., & Royo, M. (2020). Social response to early-stage government control measures of COVID-19 in Colombia: population survey, April 8-20 2020. doi:10.1101/2020.06.18.20135145		x	
Robba, H.C.S., Costa, A.A., Kozu, K.T., Silva, C.A., Farhat, S.C.L., Ferreira, J.C.d.O.A., 2022. Mental health impacts in pediatric nurses: A cross-sectional study in tertiary pediatric hospital during the COVID-19 pandemic. Revista Latino-Americana de Enfermagem Vol 30 2022, ArtID e3530. 30.		x	
Robin, C., Reynolds, R., Lambert, H., Hickman, M., Rubin, J., Smith, L. E., . . . Oliver, I. (2022). Understanding adherence to self-isolation in the first phase of COVID-19 response. doi:10.1101/2022.03.14.22272273	x		x
Robles-Bello, M.A., Sanchez-Teruel, D., Naranjo, N.V., Sohaib, L., 2022. Predictor variables of mental health in the Spanish population confined by COVID-19. Brain and Behavior. 12, e32515. https://doi.org/10.1002/brb3.2515.		x	

Robles-Bello, M.A., Sanchez-Teruel, D., Valencia Naranjo, N., 2022. Variables protecting mental health in the Spanish population affected by the COVID-19 pandemic. Current Psychology: A Journal for Diverse Perspectives on Diverse Psychological Issues. 41, 5640-5651.	x
Rodas, J.A., Jara-Rizzo, M., Oleas, D., 2021. Emotion regulation, psychological distress and demographic characteristics from an Ecuadorian sample: Data from the lockdown due to COVID-19. Data in Brief. 37, 107182. https://doi.org/10.1016/j.dib.2021.107182.	x
Rodrigues, H., Valentin, D., Franco-Luesma, E., Rakotosamimanana, V.R., Gomez-Corona, C., Saldana, E., Saenz-Navajas, M.P., 2022. How has COVID-19, lockdown and social distancing changed alcohol drinking patterns? A cross-cultural perspective between britons and spaniards. Food Quality and Preference. 95, 104344. https://doi.org/10.1016/j.foodqual.2021.104344.	x
Rodriguez-de Avila, U.E., Leon-Valle, Z.L., Ceballos-Ospino, G.A., 2020. Psychometric behavior of the Zung Self-Rating Anxiety Scale-15 (SAS-15) Spanish version, during Covid-19 Pandemic Physical Isolation. Duazary. 17, 7-9. https://doi.org/10.21676/2389783x.3469.	x
Rodriguez-Fuentes, G., Campo-Prieto, P., Cancela-Carral, J.M., 2022. Lifestyles and habits of a Spanish University Community in times of COVID-19: a cross-sectional study. Retos-Nuevas Tendencias En Educacion Fisica Deporte Y Recreacion. 283-291.	x

Rodriguez, L.M., Stewart, S.H., Neighbors, C., 2021. Effects of a Brief Web-Based Interpersonal Conflict Cognitive Reappraisal Expressive-Writing Intervention on Changes in Romantic Conflict During COVID-19 Quarantine. Couple and Family Psychology-Research and Practice. 10, 212-222. https://doi.org/10.1037/cfp0000173.	x		
Rodriguez, S., Valle, A., Pineiro, I., Rodriguez-Llorente, C., Guerrero, E., Martins, L., 2020. Sociodemographic Characteristics and Stress of People from Spain Confined by COVID-19. European Journal of Investigation in Health Psychology and Education. 10, 1095-1105. https://doi.org/10.3390/ejihpe10040077.	x		
Roe, L., Proudfoot, J., Teck, J.T.W., Irvine, R.D.G., Frankland, S., Baldacchino, A.M., 2021. Isolation, Solitude and Social Distancing for People Who Use Drugs: An Ethnographic Perspective. Frontiers in Psychiatry. 11, 623032. https://doi.org/10.3389/fpsyt.2020.623032.	x		
Rofail, D., McGale, N., Podolanczuk, A.J., Rams, A., Przydzial, K., Sivapalasingam, S., Mastey, V., Marquis, P., 2022. Patient experience of symptoms and impacts of COVID-19: a qualitative investigation with symptomatic outpatients. BMJ Open. 12, e055989.		x	x
Roitblat, Y., Burger, J., Vaiman, M., Nehuliaieva, L., Buchris, N., Shterenshis, M., 2021. Owls and larks do not exist: COVID-19 quarantine sleep habits. Sleep Medicine. 77, 177-183.	x		

Rojas-Jara, C., 2020. Quarantine, forced isolation and substance use. Cuadernos De Neuropsicologia-Panamerican Journal of Neuropsychology. 14, 24-28. https://doi.org/10.7714/cnps/14.1.203.			x			
Rojas, D.L., Torres, F.C., Garza-Ornelas, B.M., Tarquino, A.M.C., Silva, C.A.S., Chanona, J.L.A., Rodriguez-de-Ita, J., 2022. Parents and school-aged children's mental well-being after prolonged school closures and confinement during the COVID-19 pandemic in Mexico: a cross-sectional online survey study. Bmj Paediatrics Open. 6, e001468. https://doi.org/10.1136/bmjpo-2022-001468.		x				
Romay-Barja, M., Pascual-Carrasco, M., De Tena-Davila, M.J., Falcon, M., Rodriguez-Blazquez, C., Forjaz, M.J., Ayala, A., Molina-de la Fuente, I., Burgos, A., Munoz, A., Benito, A., 2021. How patients with COVID-19 managed the disease at home during the first wave in Spain: a cross-sectional study. BMJ Open. 11, e048702.				x		x
Rosca, P., Shapira, B., Neumark, Y., 2020. Isolating the isolated: Implications of COVID-19 quarantine measures on in-patient detoxification treatment for substance use disorders. International Journal of Drug Policy. 83, 102830.	x				x	
Rose, K.J., Scibilia, R., 2021. The COVID19 pandemic - Perspectives from people living with diabetes. Diabetes Research & Clinical Practice. 173, 108343.			x			

Rossi, E., Cassioli, E., Castellini, G., Sanfilippo, G., Felciai, F., Monteleone, A.M., Ricca, V., 2021. The impact of COVID-19 on eating disorders: A longitudinal study with assessments before and after the lockdown. European Psychiatry. 64, S97-S97. https://doi.org/10.1192/j.eurpsy.2021.283.			x		
Rossi, R., Socci, V., Talevi, D., Mensi, S., Niolu, C., Pacitti, F., Di Marco, A., Rossi, A., Siracusano, A., Di Lorenzo, G., 2020. COVID-19 Pandemic and Lockdown Measures Impact on Mental Health Among the General Population in Italy. Frontiers in Psychiatry. 11 (no pagination), 790.	x			x	
Rossi, R., Socci, V., Talevi, D., Mensi, S., Niolu, C., Pacitti, F., Di Marco, A., Rossi, A., Siracusano, A., Di Lorenzo, G., 2020. COVID-19 Pandemic and Lockdown Measures Impact on Mental Health Among the General Population in Italy. Frontiers in Psychiatry. 11 (no pagination), 790.		x			
Roy, D., Sinha, K., 2020. Cognitive biases operating behind the rejection of government safety advisories during COVID19 Pandemic. Asian Journal of Psychiatry. 51 (no pagination), 102048.				x	x
Roy, D., Tripathy, S., Kar, S.K., Sharma, N., Verma, S.K., Kaushal, V., 2020. Study of knowledge, attitude, anxiety & perceived mental healthcare need in Indian population during COVID-19 pandemic. Asian Journal of Psychiatry. 51, 102083.		x			

Rubio, L.A., Peng, J., Rojas, S., Rojas, S., Crawford, E., Black, D., Jacobo, J., Tulier-Laiwa, V., Hoover, C.M., Martinez, J., Jones, D., Sachdev, D., Cox, C., Herrera, E., Valencia, R., Zurita, K.G., Chamie, G., DeRisi, J., Petersen, M., Havlir, D.V., Marquez, C., Consortium, C., 2021. The COVID-19 Symptom to Isolation Cascade in a Latinx Community: A Call to Action. Open Forum Infectious Diseases. 8, ofab023. https://doi.org/10.1093/ofid/ofab023.	x		x
Ruiz-Frutos, C., Ortega-Moreno, M., Allande-Cusso, R., Dominguez-Salas, S., Dias, A., Gomez-Salgado, J., 2021. Health-related factors of psychological distress during the COVID-19 pandemic among non-health workers in Spain. Safety Science. 133 (no pagination), 104996.		x	
Ruiz-Perez, J.I., Barrera, J.A., 2020. Spatial and temporal analysis of the breach of legal regulations regarding the Colombian COVID-19 lockdown. Logos Ciencia & Tecnologia. 12, 20-32. https://doi.org/10.22335/rlct.v12i3.1251.		x	
Ruiz, P., Semblat, F., Pautassi, R.M., 2022. Change in Psychoactive Substance Consumption in Relation to Psychological Distress During the COVID-19 Pandemic in Uruguay. Sultan Qaboos University Medical Journal. 22, 198-205.		x	
Ryu, S., Hwang, Y., Yoon, H., Chun, B.C., 2022. Self-Quarantine Noncompliance During the COVID-19 Pandemic in South Korea. Disaster Medicine & Public Health Preparedness. 16, 464-467.	x		x

Saetrevik, B., Bjorkheim, S.B., 2022. Motivational factors were more important than perceived risk or optimism for compliance to infection control measures in the early stage of the COVID-19 pandemic. PLoS ONE. 17(9 September) (no pagination), e0274812.			x	x	
Saez-Delgado, F., Olea-Gonzalez, C., Mella-Norambuena, J., Lopez-Angulo, Y., Garcia-Vasquez, H., Cobo-Rendon, R., Lopez, F.S., 2020. Psychosocial Characterization and Mental Health in Families of Chilean Students during Physical Isolation by Covid-19. Revista Internacional De Educacion Para La Justicia Social. 9, 281-300. https://doi.org/10.15366/riejs2020.9.3.015.	x				
Sagaltici, E., Saydam, R.B., Cetinkaya, M., Sahin, S.K., Kucuk, S.H., Muslumanoglu, A.Y., 2022. Burnout and psychological symptoms in healthcare workers during the COVID-19 pandemic: Comparisons of different medical professions in a regional hospital in Turkey. Work. 72, 1077-1085.		x			x
Saguem, B.N., Braham, A., Romdhane, I., Ben Nasr, S., 2021. Psychological impact of home confinement due to coronavirus disease on medical students and its relationship with cognitive emotion regulation. Journal of Mental Health Training Education and Practice. 16, 322-337. https://doi.org/10.1108/jmhtep-11-2020-0085.	x				
Saguem, B.N., Nakhli, J., Romdhane, I., Nasr, S.B., 2022. Predictors of sleep quality in medical students during COVID-19 confinement. Encephale. 48, 3-12.	x				

Sahin, I., Toluk, O., Kaskir Kesin, F., Uzunoglu, A., Yabaci Tak, A., Ercan, I., 2022. Compliance with General Rules and Periodically Differences During the COVID-19 Pandemic in Turkiye: A Cross-Sectional Study. Turkiye Klinikleri Journal of Medical Sciences. 42(4), 297-310.	x			x
Sahu, A., Naqvi, W.M., 2020. Floating countries and corona pandemic: Impact of covid-19 on stranded cruise ships. International Journal of Research in Pharmaceutical Sciences. 11(Special Issue 1), 219-223.			x	x
Sallie, S.N., Ritou, V., Bowden-Jones, H., Voon, V., 2020. Assessing international alcohol consumption patterns during isolation from the COVID-19 pandemic using an online survey: highlighting negative emotionality mechanisms. BMJ Open. 10, e044276.		x		
Sanchez-Hernandez, O., Canales, A., 2020. EFFICACY AND SATISFACTION OF THE RESILIENCE AND WELLBEING PROGRAM: "STAY AT HOME" PSYCHOLOGY IN TIMES OF QUARANTINE AND PANDEMIC. Revista De Psicoterapia. 31, 381-398. https://doi.org/10.33898/rdp.v31i117.389.		x		
Santamaria-Garcia, H., Burgaleta, M., Legaz, A., Flichtentrei, D., Cordoba-Delgado, M., Molina-Paredes, J., Linares-Puerta, J., Montealegre-Gomez, J., Castelblanco, S., Schulte, M., Paramo, J.D., Mondragon, I., Leongomez, J.D., Salamone, P., Gonzalez-Pacheco, J., Baez, S., Eyre, H., Ibanez, A., 2022. The price of prosociality in pandemic times. Humanities & Social Sciences Communications. 9, 15. https://doi.org/10.1057/s41599-021-01022-2.			x	x

Santangelo, G., Baldassarre, I., Barbaro, A., Cavallo, N.D., Cropano, M., Maggi, G., Nappo, R., Trojano, L., Raimo, S., 2021. Subjective cognitive failures and their psychological correlates in a large Italian sample during quarantine/self-isolation for COVID-19. Neurological Sciences. 42, 2625-2635.	x	
Santelesforo, R.G., del Rey, T.R., Perez-Saez, E., Hernandez, B.P., Impact of confinement measures due to the COVID-19 pandemic on people living with dementia and their caregivers in Spain. Health & Social Care in the Community. https://doi.org/10.1111/hsc.13960.	x	
Sarah, L.T., Myriam, C.W., Lorenz, R., Nadia, W., Thomas, L., Dorothea, H., Michael, R., Martin, R., Philipp, K., Pietro, V., Christian, R.K., Felix, F., Alexia, C., Tomas, V.K., Sandra, C., Matthias, P., 2020. Flattening the curve in 52 days: characterisation of the COVID-19 pandemic in the Principality of Liechtenstein - an observational study. Swiss Medical Weekly. 150, w20361. https://doi.org/10.4414/smw.2020.20361.		x

Sarfati, S., Katz, A., Cohen, M., Bantman, P., Mimoun, A., Sitruk, P., Amson, F., Rimmer, R., Zittoun, J., Paillat, S., Levy, V., Pariente, J., Huet, C., Sztulman, L., Wargnier, N., Soussan, A., Bloch, G., Ghozlan, E., Michower, M., Fisbein, L., Hazan, K., Battner, H., Heymann, M., Astruc, A., Halioua, D., Taieb, J., Journo, M., Odier, R., Dassa, S., Rochmann, G., Vaislic, M., Taieb, C., Halioua, B., 2022. Psychological impact of the outbreak of COVID-19 on Holocaust survivors in France. European Journal of Trauma & Dissociation Vol 6(2), 2022, ArtID 100242. 6.	x			
Sarkar, A., Jasmine, E., Thomas, S.L., Andrade, C., 2022. Markers of Social and Emotional Health in College Students During the COVID-19 Pandemic. The Primary Care Companion to CNS Disorders. 24, 10.			x	x
Sarmiento, A.S., Ponce, R.S., Bertolin, A.G., 2021. Resilience and COVID-19. An Analysis in University Students during Confinement. Education Sciences. 11, 533. https://doi.org/10.3390/educsci11090533.	x			
Sarvari, S., Rahimzadeh, M., Saei, S.M., Salehian, M.H., 2022. Do the Adolescents' Physical Activity and Screen Time during the COVID-19 Quarantine correlate to their Upper Extremity Abnormalities and Anxiety? International Journal of Pediatrics-Mashhad. 10, 15567-15576. https://doi.org/10.22038/ijp.2022.62477.4779.	x			
Saurabh, K., Ranjan, S., 2020. Compliance and Psychological Impact of Quarantine in Children and Adolescents due to Covid-19 Pandemic. Indian Journal of Pediatrics. 87, 532-536.		x		

Savvopoulou, N., Assimakopoulos, K., Gourzis, P., Jelastopulu, E., 2022. Eating habits during quarantine: Investigating the role of emotions and loneliness in a sample of adults in Greece. European Psychiatry. 65(Supplement 1), S534.		x								
Sawant, N., Ingawale, S., Lokhande, U., Patil, S., Ayub, E.F.M., Rathi, V., 2021. Psychiatric Sequelae and COVID Experiences of Post COVID -19 Recovered Resident Doctors and Interns of a Tertiary General Hospital in Mumbai. Journal of the Association of Physicians of India. 69, 22-26.			x							
Schluter, P.J., Genereux, M., Landaverde, E., Chan, E.Y.Y., Hung, K.K.C., Law, R., Mok, C.P.Y., Murray, V., O'Sullivan, T., Qadar, Z., Roy, M., 2022. An eight country cross-sectional study of the psychosocial effects of COVID-19 induced quarantine and/or isolation during the pandemic. Scientific Reports. 12, 13175.	x						x			
Schmitt, A., Brenner, A.M., Primo de Carvalho Alves, L., Claudino, F.C.d.A., Fleck, M.P.d.A., Rocha, N.S., 2021. Potential predictors of depressive symptoms during the initial stage of the COVID-19 outbreak among Brazilian adults. Journal of Affective Disorders. 282, 1090-1095.		x								
Scholz, J., Wetzker, W., Licht, A., Heintzmann, R., Scherag, A., Weis, S., Pletz, M., Betsch, C., Bauer, M., Dickmann, P., Co, N.A.N.s.g., 2021. The role of risk communication in public health interventions. An analysis of risk communication for a community quarantine in Germany to curb the SARS-CoV-2 pandemic. PLoS ONE [Electronic Resource]. 16, e0256113.							x			x

Schorr, A.V., Yehuda, I., Tamir, S., 2021. Ethnic Differences in Loneliness, Depression, and Malnutrition Among Older Adults During COVID-19 Quarantine. Journal of Nutrition, Health & Aging. 25, 311-317.		x	
Schuch, F.B., Bulzing, R.A., Meyer, J., Lopez-Sanchez, G.F., Grabovac, I., Willeit, P., Vancampfort, D., Caperchione, C.M., Sadarangani, K.P., Werneck, A.O., Ward, P.B., Tully, M., Smith, L., 2022. Moderate to vigorous physical activity and sedentary behavior changes in self-isolating adults during the COVID-19 pandemic in Brazil: a cross-sectional survey exploring correlates. Sport Sciences for Health. 18, 155-163. https://doi.org/10.1007/s11332-021-00788-x.		x	
Schuch, F.B., Bulzing, R.A., Meyer, J., Vancampfort, D., Firth, J., Stubbs, B., Grabovac, I., Willeit, P., Tavares, V.D.O., Calegaro, V.C., Deenik, J., Lopez-Sanchez, G.F., Veronese, N., Caperchione, C.M., Sadarangani, K.P., Abufaraj, M., Tully, M.A., Smith, L., 2020. Associations of moderate to vigorous physical activity and sedentary behavior with depressive and anxiety symptoms in self-isolating people during the COVID-19 pandemic: A cross-sectional survey in Brazil. Psychiatry Research. 292, 113339.		x	
Senol, Y., Avci, K., 2022. Identification of risk factors that increase household transmission of COVID-19 in Afyonkarahisar, Turkey. Journal of Infection in Developing Countries. 16, 927-936.	x		x

Senthil, M., Gayathri, N., 2022. Quarantined individual's behavior- a model evidence from COVID -19 pandemic. Journal of Human Behavior in the Social Environment. 32, 417-425. https://doi.org/10.1080/10911359.2021.1901825.	x
Serin, E., Koc, M.C., 2020. Examination of the eating behaviours and depression states of the university students who stay at home during the coronavirus pandemic in terms of different variables. Progress in Nutrition. 22, 33-43.	x
Serralta, F.B., Zibetti, M.R., Evans, C., 2020. Psychological Distress of University Workers during COVID-19 Pandemic in Brazil. International Journal of Environmental Research & Public Health [Electronic Resource]. 17, 17.	x
Sfendla, A., Hadrya, F., 2020. Factors Associated with Psychological Distress and Physical Activity During the COVID-19 Pandemic. Health Security. 18, 444-453.	x
Shafiyeva, E., Aliyeva, S., Kazimova, K., Valiyeva, Y., Nasirova, N., Babayeva, T., 2022. PSYCHOLOGICAL NATURE OF THE IMPACT OF PANDEMIC ON THE EMOTIONAL STATE OF THE POPULATION IN AZERBAIJAN. Revista Universidad Y Sociedad. 14, 188-201.	x
Shah, S.M.A., Mohammad, D., Qureshi, M.F.H., Abbas, M.Z., Aleem, S., 2020. Prevalence, psychological responses and associated correlates of depression, anxiety and stress in a global population, during the coronavirus disease (covid-19) pandemic. Community Mental Health Journal. No Pagination Specified.	x

Shah, Y.B., Kjelstrom, S., Martinez, D., Leitenberger, A., Manasseh, D.M., Bollmann-Jenkins, M., Partridge, A., Kaklamani, V., Chlebowski, R., Larson, S., Weiss, M., 2022. Risk factors for heightened COVID-19-Related anxiety among breast cancer patients. Cancer Medicine.			x										
Shaikhain, T.A., Al-Husayni, F.A., Alhejaili, E.A., Al-Harbi, M.N., Bogari, A.A., Baghlaf, B.A., Alzahrani, M.S., 2021. COVID-19-Related Knowledge and Practices Among Health Care Workers in Saudi Arabia: Cross-sectional Questionnaire Study. Jmir Formative Research. 5, e21220. https://doi.org/10.2196/21220.								x			x		
Shen, S. H., et al. (2022). "Investigation on psychosomatic status of entry quarantine personnel during the COVID-19 pandemic." Food Science and Technology 42.								x					x
Shewasinad Yehualashet, S., et al. (2021). "Predictors of adherence to COVID-19 prevention measure among communities in North Shoa Zone, Ethiopia based on health belief model: A cross-sectional study." PLoS ONE [Electronic Resource] 16(1): e0246006.	x										x		
Shillitoe, S., et al. (2022). "Improving wellbeing for COVID-19 patients at umm salal isolation and recovery facility through expressive drawing (Art Therapy): A Service/Quality Improvement Initiative." Journal of Emergency Medicine, Trauma and Acute Care. Conference: Qatar Health(pagination).								x				x	
Sinelnikova, E. S. (2021). "Emotional response and coping on self-isolation of students with different levels of situational life satisfaction." Voprosy Psikhologii(6): 43-+.					x								

Singh, S. M., et al. (2020). "Is it time to consider an income guarantee for the period that patients with COVID-19 spend in isolation: an Indian perspective." Public Health 185: 3-3.							x				
Smith, L. E., et al. (2020). "Factors associated with adherence to self-isolation and lockdown measures in the UK: a cross-sectional survey." Public Health 187: 41-52.	x										x
Smith, L. E., et al. (2020). "The impact of believing you have had COVID-19 on self-reported behaviour: Cross-sectional survey." PLoS ONE 15(11 November) (no pagination).			x								
Smith, L. E., et al. (2021). "Adherence to the test, trace, and isolate system in the UK: Results from 37 nationally representative surveys." The BMJ 372 (no pagination).	x										x
Smith, L. E., et al. (2021). "Intention to adhere to test, trace, and isolate during the COVID-19 pandemic (the COVID-19 Rapid Survey of Adherence to Interventions and Responses study)." British journal of health psychology. 30.									x		x
Son, H. M., et al. (2021). "The Lived Experiences of COVID-19 Patients in South Korea: A Qualitative Study." International Journal of Environmental Research & Public Health [Electronic Resource] 18(14): 12.				x							
Sorokin, M. Y., et al. (2021). Psychiatria Danubina 33(3): 386-392.			x								
Steens, A., et al. (2020). "Poor self-reported adherence to COVID-19-related quarantine/isolation requests, Norway, April to July 2020." Eurosurveillance 25(37) (no pagination).	x										x

Stingone, C., et al. (2022). "Features of fragile people with SARS-CoV-2 infection in isolation in a COVID-19 hotel in Rome, Italy." European Review for Medical and Pharmacological Sciences 26(7): 2631-2638.						x			x	
Sun, P., et al. (2020). "AN ANALYSIS OF THE DIFFERENCES IN ANXIETY AND EDUCATIONAL BACKGROUND AMONG PATIENTS ISOLATING DUE TO COVID-19." Acta Medica Mediterranea 36(6): 3807-3810.		x								
Sun, P., et al. (2022). "Impact of gender difference on anxiety in COVID-19 patients in quarantine wards." Frontiers in Psychiatry Vol 12 2022, ArtID 799879 12.		x								
Sun, P., et al. (2022). "The impact of age on anxiety in Covid-19 patients in quarantine wardwards." Psychology Health & Medicine 27(2): 403-408.		x								
Suplico-Jeong, L., et al. (2022). "Adherence to quarantine protocols to prevent the spread of COVID-19: the mediating effect of intrinsic and extrinsic motivations." Asian Education and Development Studies 11(2): 366-379.	x									
Surenthirakumaran, R., et al. (2022). "Depression and associated factors from COVID-19-related quarantine in Jaffna district, Sri Lanka." Asia-Pacific Journal of Public Health 34(4): 413-415.				x						
Suresh, P., et al. (2021). "Establishment of Telemedicine for Covid-19 Affected Patients at Home Isolation in a Secondary Care Hospital in the Context of Covid-19 Pandemic." BMJ Leader 5(Supplement 1): A8.						x				x

Synek, M., et al. (2022). "'Everything's the Same, Everything's Halted': Quarantine in a 'Home' for People Identified as Disabled." Sociologicky Casopis-Czech Sociological Review 58(5): 563-585.	x		
Szwarcwald, C. L., et al. (2020). "Adherence to physical contact restriction measures and the spread of COVID-19 in Brazil." Epidemiologia E Servicos De Saude 29(5).	x		
Tamm, A. L., et al. (2022). "Changes in the Mental Health Indicators and Training Opportunities for Estonian Elite Athletes Compared to the COVID-19 Isolation Period." Sports 10(5).	x		
Tan, S. T. and L. Lee (2022). "Social determinants of self-reported psychological distress during the covid-19 pandemic: A cross-sectional study." Psychology, Health & Medicine: No Pagination Specified.	x		
Tang, F., et al. (2021). "COVID-19 related depression and anxiety among quarantined respondents." Psychology & Health 36(2): 164-178.		x	
Tang, K. H. D. (2022). "Movement control as an effective measure against Covid-19 spread in Malaysia: an overview." Journal of Public Health (Germany) 30(3): 583-586.			x
Tang, Q., et al. (2022). "Effect of Repeated Home Quarantine on Anxiety, Depression, and PTSD Symptoms in a Chinese Population During the COVID-19 Pandemic: A Cross-sectional Study." Frontiers in Psychiatry 13.	x		

Tang, W. J., et al. (2020). "Prevalence and correlates of PTSD and depressive symptoms one month after the outbreak of the COVID-19 epidemic in a sample of home-quarantined Chinese university students." Journal of Affective Disorders 274: 1-7.						x						x
Taporoski, T., et al. (2021). "Sex and age differences in the association between quarantine and sleep quality during COVID-19 pandemic in Brazil." American Journal of Human Biology 33.	x											
Taskesen, B., et al. "Evaluation of depression, anxiety and posttraumatic stress response levels of children and adolescents treated with COVID-19." European Journal of Pediatrics.						x						x
Theano, P., et al. (2020). "SARSCOV-2 PSYCHOSOMATIC EFFECTS AND FEAR OF STIGMA ON THE DISCHARGE DAY OF INFECTED INDIVIDUALS: SAPFO STUDY." Psychiatria Danubina 32(3-4): 577-580.		x										
Thorneloe, R. J., et al. (2022). "Adherence to behaviours associated with the test, trace, and isolate system: an analysis using the theoretical domains framework." BMC Public Health 22(1): 567.							x		x			
TMGH-Global. Perceived Stress of Quarantine and Isolation During COVID-19 Pandemic: A Global Survey. (2021). Frontiers in Psychiatry, 12 (no pagination), Article 656664.		x										
Tokur Kesgin, M., et al. (2022). "Comparison of anxiety levels of hospitalized COVID-19 patients, individuals under quarantine, and individuals in society." Perspectives in Psychiatric Care 58(1): 149-158.						x					x	

Toulabi, T., et al. (2021). "Exploring COVID-19 patients' experiences of psychological distress during the disease course: a qualitative study." BMC Psychiatry 21(1): 625.			x		x
Tseng, C. W., et al. (2021). "Patients' Compliance With Quarantine Requirements for Exposure or Potential Symptoms of COVID-19." Hawaii Journal of Health and Social Welfare 80(11): 276-282.	x			x	
Tsukawaki, R. and T. Imura (2021). "The relationship between self-isolation during lockdown and individuals' depressive symptoms: Humor as a moderator." Social Behavior and Personality 49(7).		x			
Tsuzuki, S., et al. (2021). "The burden of isolation to the individual: a comparison between isolation for COVID-19 and for other influenza-like illnesses in Japan." Epidemiology & Infection 150: e5.			x		x
Tyson, L., et al. (2022). "The effects of social distancing and self-isolation during the COVID-19 pandemic on adults diagnosed with asthma: A qualitative study." Journal of Health Psychology 27(6): 1408-1420.		x			
Ubara, A., et al. (2020). "Self-isolation due to COVID-19 is linked to small one-year changes in depression, sleepiness, and insomnia: Results from a clinic for sleep disorders in Shiga Prefecture, Japan." International Journal of Environmental Research and Public Health 17(23): 1-11.		x			
Udaya Bahadur, B. C., et al. (2021). "Anxiety and depression among people living in quarantine centers during COVID-19 pandemic: A mixed method study from western Nepal." PLoS ONE 16(7 July) (no pagination).			x		x

Uludag, A. and K. Safak (2022). "A QUALITATIVE EVALUATION OF THE VIEWS OF THOSE STAYING IN DORMITORY-HOSPITALS DURING THE COVID-19 PANDEMIC. [Turkish]." Nobel Medicus 18(1): 22-32.	x											
Usher, K., et al. (2020). "COVID-19 and social restrictions: the potential mental health impact of social distancing and isolation for young Indigenous Australians." Australasian Psychiatry 28(5): 599-600.					x							
Ustundag, G., et al. (2022). "Evaluation of Depression, Anxiety, and Sleep Quality in Children Diagnosed With COVID-19." Journal of Nervous and Mental Disease 210(8): 629-632.						x						x
Vasiliu, O., et al. (2021). "Quality of life impairments and stress coping strategies during the Covid-19 pandemic isolation and quarantine - A Web-based survey." Romanian Journal of Military Medicine 124(1): 10-21.						x				x		
Vasquez, G., et al. (2020). "Mental health, confinement, and coronavirus concerns: A qualitative study." Revista Interamericana de Psicologia 54(2): 1-16.	x											
Vaysse, J. and A. Poli (2020). "Jail incarceration, social sanitary confinement and ways of escape." Annales Medico-Psychologiques 178(7): 743-746.						x			x			
Vazquez-Nava, F., et al. (2021). "Risk factors of non-adherence to guidelines for the prevention of COVID-19 among young adults with asthma in a region with a high risk of a COVID-19 outbreak." Journal of Asthma 58(12): 1630-1636.	x											

Venkatesan, K., et al. (2021). "Assessment of Psychological Stress among Quarantined COVID-19 Patients." Journal of Pharmaceutical Research International 33(44A): 502-507.							x				x			
Venkatesh, V., et al. (2021). "Psychological impact of infection with SARS-CoV-2 on health care providers: A qualitative study." Journal of Family Medicine and Primary Care 10(4): 1666-1672.							x							x
Vera, C. A. R. (2021). "The dynamic characteristics of mental health in Peruvian adults during the COVID-19 quarantine." Medisur-Revista De Ciencias Medicas De Cienfuegos 19(2): 318-322.		x												
Verberk, J. D. M., et al. (2021). "Experiences and needs of persons living with a household member infected with SARS-CoV-2: A mixed method study." PLoS ONE 16(3 March 2021) (no pagination).	x						x							
Vuorio, A., et al. (2022). "Promoting Flight Crew Mental Health Requires International Guidance for Down-Route Quarantine Circumstances." Frontiers in Public Health 10: 854262.						x								
Wahyuni, A. A. S., et al. (2022). "Relationship between the Length of Isolation and Swab Results with Degree of Anxiety and Depression Disorders in Patients with Confirmed of Coronavirus Disease 2019." Open Access Macedonian Journal of Medical Sciences 10(T7): 170-175.		x												
Walker, L. J., et al. (2021). "SARS-CoV-2 infections among Australian passengers on the Diamond Princess cruise ship: A retrospective cohort study." PLoS ONE 16(9).							x			x				

Walsh, B., et al. (2022). "Social stressors and isolation have biggest effect on resident wellness during a pandemic." Western Journal of Emergency Medicine 23(1.1): S33.	x			
Wang, C. Y., et al. (2021). "Rapid creation of a medical respite center for covid-19 positive individuals experiencing homelessness." Journal of General Internal Medicine 36(SUPPL 1): S401.			x	x
Wang, D., et al. (2022). "Trajectories of mental health status during the early phase pandemic in China: A longitudinal study on adolescents living in the community with confirmed cases." Psychiatry Research 314: 114646.	x			
Wang, M. H., et al. (2021). "Acute psychological impact on COVID-19 patients in Hubei: a multicenter observational study." Translational Psychiatry 11(1).		x		
Wang, W., et al. (2021). "Psychological impact of mandatory covid-19 quarantine on small business owners and self-employed in china." Current Psychology: A Journal for Diverse Perspectives on Diverse Psychological Issues: No Pagination Specified.	x			
Wang, Z. H., Qi, S. G., Zhang, H., Mao, P. X., He, Y. L., Li, J., Xiao, S. Y., Peng, H. M., Sun, W. W., Guo, H. Y., & Liu, M. (2020). Impact of the COVID-19 epidemic on anxiety among the elderly in community. [Chinese]. *Zhonghua yi xue za zhi*, *100(40)*, 3179-3185.	x			

Weiden, F., Levinsky, M., Schiff, M., Becker, N., Pat-horenczyk, R., & Benbenishty, R. (2021). COVID-related concerns, the need for help, and perceived microagressions among young ultra-orthodox jewish respondents in Israel. *International Journal of Environmental Research and Public Health*, *18(12) (no pagination)*, Article 6445.	x
Weinstein, N., & Nguyen, T. V. (2020). Motivation and preference in isolation: a test of their different influences on responses to self-isolation during the COVID-19 outbreak. *Royal Society Open Science*, *7*(5), Article 200458. https://doi.org/10.1098/rsos.200458	x
Werneck, A. O., Silva, D. R., Malta, D. C., Souza-Junior, P. R. B., Azevedo, L. O., Barros, M. B. A., & Szwarcwald, C. L. (2021). Physical inactivity and elevated TV-viewing reported changes during the COVID-19 pandemic are associated with mental health: A survey with 43,995 Brazilian adults [Research Support, Non-U.S. Gov't]. *Journal of Psychosomatic Research*, *140*, 110292.	x
Werneck, A. O., Silva, D. R., Malta, D. C., Souza, P. R. B., Azevedo, L. O., Barros, M. B. A., & Szwarcwald, C. L. (2021). Changes in the clustering of unhealthy movement behaviors during the COVID-19 quarantine and the association with mental health indicators among Brazilian adults. *Translational Behavioral Medicine*, *11*(2), 323-331. https://doi.org/10.1093/tbm/ibaa095	x

Wessely, S., Tappiser, M., Eisenburger, N., Feddern, S., Gehlhar, A., Kilimann, A., Klee, L., Niesen, J., Schmidt, N., Wiesmuller, G. A., Kossow, A., Grune, B., Joisten, C., & CoCo-Fakt, G. (2022). Changes in Alcohol Consumption, Eating Behaviors, and Body Weight during Quarantine Measures: Analysis of the CoCo-Fakt Study. *Obesity Facts*, *15*(4), 570-580.	x		x
Whitehead, B. R., & Torossian, E. (2021). Older Adults' Experience of the COVID-19 Pandemic: A Mixed-Methods Analysis of Stresses and Joys. *Gerontologist*, *61*(1), 36-47.		x	
Willoughby, J. F., King, R. L., & Adams, P. M. Development of an mHealth text message intervention to promote adherence to COVID-19 isolation recommendations for college students. *Journal of American College Health*. https://doi.org/10.1080/07448481.2022.2037615		x	
Wilson, J. M., Lee, J., & Shook, N. J. (2021). COVID-19 worries and mental health: the moderating effect of age [Research Support, Non-U.S. Gov't]. *Aging & Mental Health*, *25*(7), 1289-1296.		x	
Wolff, W., Martarelli, C. S., Schuler, J., & Bieleke, M. (2020). High Boredom Proneness and Low Trait Self-Control Impair Adherence to Social Distancing Guidelines during the COVID-19 Pandemic. *International Journal of Environmental Research & Public Health [Electronic Resource]*, *17*(15), 28.		x	
Woo, S. W., & Hong, N. (2021). Study on the mental health status of individuals self-quarantined due to the COVID-19 in a city. *Asia-Pacific Psychiatry*, *13*. <Go to ISI>://WOS:000637802700029		x	

Wratil, P. R., Kotter, K., Bischof, M. L., Hollerbach, S., Apak, E., Kalteis, A. L., Nayeli-Pflantz, T., Kaderali, L., Adorjan, K., & Keppler, O. T. Vaccine-hesitant individuals accumulate additional COVID-19 risk due to divergent perception and behaviors related to SARS-CoV-2 testing: a population-based, cross-sectional study. *Infection*. https://doi.org/10.1007/s15010-022-01947-z		x	x
Wright, L., Steptoe, A., & Fancourt, D. (2020). What predicts adherence to COVID-19 government guidelines? Longitudinal analyses of 51,000 UK adults. doi:10.1101/2020.10.19.20215376	x		
Wright, M. F., & Wachs, S. (2022). Self-isolation during the beginning of the COVID-19 pandemic and adolescents' health outcomes: The moderating effect of perceived teacher support. *School Psychologist*, *37*(1), 47-53.	x		
Wright, M. F., & Wachs, S. Self-isolation practices and perceived social support from friends: the impact on adolescents' mental health. *European Journal of Developmental Psychology*. https://doi.org/10.1080/17405629.2022.2146090	x		
Wu, C., Zhou, T., Wu, L. P., Zhao, Y. X., Shang, H. M., Gao, Y. Y., Rao, P., Jiao, Y., & Xi, J. N. (2021). Analysis of psychological status and effect of psychological intervention in quarantined population during the epidemic of SARS-CoV-2 [Observational Study]. *Medicine*, *100*(19), e25951.	x		

Wu, H. C., Chen, S. X., & Xu, H. N. Exploring the drivers of COVID-19 protective behaviors among Singaporean tourists to Indonesia using travel bubbles. *Current Psychology*. https://doi.org/10.1007/s12144-022-03629-3							x		x		
Xiao, C. X., Lin, Y. J., Lin, R. Q., Liu, A. N., Zhong, G. Q., & Lan, C. F. (2020). Effects of progressive muscle relaxation training on negative emotions and sleep quality in COVID-19 patients: A clinical observational study [Observational Study]. *Medicine*, *99*(47), e23185.						x					x
Xiao, H., Zhang, Y., Kong, D. S., Li, S. Y., & Yang, N. X. (2020). Social Capital and Sleep Quality in Individuals Who Self-Isolated for 14 Days During the Coronavirus Disease 2019 (COVID-19) Outbreak in January 2020 in China. *Medical Science Monitor*, *26*, Article e923921. https://doi.org/10.12659/msm.923921	x										
Xu, C., Xu, Y. J., Xu, S., Zhang, Q. H., Liu, X. T., Shao, Y. F., Xu, X. X., Peng, L., & Li, M. (2020). Cognitive Reappraisal and the Association Between Perceived Stress and Anxiety Symptoms in COVID-19 Isolated People. *Frontiers in Psychiatry*, *11*, Article 858. https://doi.org/10.3389/fpsyt.2020.00858	x										
Xu, X., Chew, K. A., Xu, X. L., Wu, Z. H., Xiao, X. H., & Yang, Q. (2021). Demographic and social correlates and indicators for behavioural compliance with personal protection among Chinese community-dwellers during COVID-19: a cross-sectional study. *BMJ Open*, *11*(1), Article e041453. https://doi.org/10.1136/bmjopen-2020-041453	x										

Xue, D. N., Liu, T., Chen, X. M., Liu, X. R., & Chao, M. (2021). Data on media use and mental health during the outbreak of COVID-19 in China. *Data in Brief*, *35*, Article 106765. https://doi.org/10.1016/j.dib.2021.106765		x			
Yan, L. Y., Liu, F. D., & Meng, X. (2022). Questionnaires assessing the anxiety alleviation benefits of indoor plants for self-isolated population during COVID-19. *International Journal of Low-Carbon Technologies*, *17*, 300-307. https://doi.org/10.1093/ijlct/ctab102		x			
Yan, T., Zhizhong, W., Jianzhong, Z., Yubo, Y., Jie, L., Junjun, Z., & Guangtian, L. (2021). Depressive and Anxiety Symptoms Among People Under Quarantine During the COVID-19 Epidemic in China: A Cross-Sectional Study. *Frontiers in Psychiatry*, *12 (no pagination)*, Article 566241.			x		x
Yang, L., Mitchell, D., Clayton, F., Clerc, P., Spangehl, T., Oxland, R., Spearing, K., Barbic, S., Andolfatto, G., Grafstein, E., Scheuermeyer, F., & Kestler, A. (2022). Self-isolation among discharged emergency department patients with suspected COVID-19 [Letter]. *CJEM Canadian Journal of Emergency Medical Care*, *24*(1), 97-98.	x			x	
Yi-Chi Chang, Y., Wu, P. L., & Chiou, W. B. (2021). Thoughts of social distancing experiences affect food intake and hypothetical binge eating: Implications for people in home quarantine during COVID-19 [Randomized Controlled Trial]. *Social Science & Medicine*, *284*, 114218.		x			

Yildirim, H., Isik, K., & Aylaz, R. (2021). The effect of anxiety levels of elderly people in quarantine on depression during covid-19 pandemic. *Social Work in Public Health*, *36*(2), 194-204.	x		
Yu, J. C., Yang, Z. Y., Wu, Y. Q., Ge, M., Tang, X. M., & Jiang, H. B. (2021). Prevalence of and Factors Associated With Depressive Symptoms Among College Students in Wuhan, China During the Normalization Stage of COVID-19 Prevention and Control. *Frontiers in Psychiatry*, *12*, Article 742950. https://doi.org/10.3389/fpsyt.2021.742950	x		
Yuan, Z., Musa, S. S., Hsu, S. C., Cheung, C. M., & He, D. (2022). Post pandemic fatigue: what are effective strategies? *Scientific Reports*, *12*(1), 9706.			x
Zahangir, M. S., & Rokonuzzaman, M. (2022). Depression, anxiety and stress among people infected with COVID-19 in Dhaka and Chittagong cities. *Heliyon*, *8*(9), Article e10415. https://doi.org/10.1016/j.heliyon.2022.e10415		x	
Zainel, A. A., Qotba, H., Al-Maadeed, A., Al-Kohji, S., Al Mujalli, H., Ali, A., Al Mannai, L., Aladab, A., AlSaadi, H., AlKarbi, K. A., & Al-Baghdadi, T. (2021). Psychological and Coping Strategies Related to Home Isolation and Social Distancing in Children and Adolescents During the COVID-19 Pandemic: Cross-sectional Study. *Jmir Formative Research*, *5*(4), Article e24760. https://doi.org/10.2196/24760	x		

Zakeri, M. A., Maazallahi, M., Ehsani, V., & Dehghan, M. (2021). Iranian psychosocial status during and after COVID-19 outbreak mandatory quarantine: A cross-sectional study [Research Support, Non-U.S. Gov't]. *Journal of Community Psychology*, *49*(7), 2506-2516.	x		
Zavala-Flores, E., Salcedo-Matienzo, J., Quiroz-Alva, A., & Berrocal-Kasay, A. (2020). [Effects of the COVID-19 quarantine on patients with rheumatoid arthritis and systemic lupus erythematosus] [Letter]. *Revista Peruana de Medicina Experimental y Salud Publica*, *37*(4), 783-784.	x		
Zeiler, M., Wittek, T., Kahlenberg, L., Grobner, E. M., Nitsch, M., Wagner, G., Truttmann, S., Krauss, H., Waldherr, K., & Karwautz, A. (2021). Impact of covid-19 confinement on adolescent patients with anorexia nervosa: A qualitative interview study involving adolescents and parents. *International Journal of Environmental Research and Public Health*, *18(8) (no pagination)*, Article 4251.	x		
Zhang, G. Y., Liu, Q., Lin, J. Y., Yan, L., Shen, L., & Si, T. M. (2021). Mental health outcomes among patients from Fangcang shelter hospitals exposed to coronavirus disease 2019: An observational cross-sectional study. *Chronic Diseases and Translational Medicine*, *7(1)*, 57-64.		x	x

Zhang, W. Z., LaBedz, S. L., Holbrook, J. T., Gangemi, A., Baalachandran, R., Eakin, M. N., Wise, R. A., Sumino, K., & Amer Lung Assoc Airways Clinical, R. (2022). Impact of the Coronavirus Disease 2019 Pandemic on Physical and Mental Health of Patients With COPD: Results From a Longitudinal Cohort Study Conducted in the United States (2020-2021). *Chronic Obstructive Pulmonary Diseases-Journal of the Copd Foundation*, *9*(4), 510-519. https://doi.org/10.15326/jcopdf.2022.0287	x	
Zhang, Y. Q., Zhan, N. L., Zou, J. Q., Xie, D. J., Liu, M. F., & Geng, F. L. (2022). The transmission of psychological distress and lifestyles from parents to children during COVID-19. *Journal of Affective Disorders*, *303*, 74-81. https://doi.org/10.1016/j.jad.2022.02.007	x	
Zhang, Z. Y., Feng, Y., Song, R., Yang, D., & Duan, X. F. (2021). Prevalence of psychiatric diagnosis and related psychopathological symptoms among patients with COVID-19 during the second wave of the pandemic. *Globalization and Health*, *17*(1), Article 44. https://doi.org/10.1186/s12992-021-00694-4	x	
Zhao, Q., Hu, C., Feng, R., & Yang, Y. (2020). Investigation of the mental health of patients with COVID-19. [Chinese]. *Chinese Journal of Neurology*, *53(6)*, 432-436.		x

Zhao, S. Z., Wong, J. Y. H., Wu, Y. D., Choi, E. P. H., Wang, M. P., & Lam, T. H. (2020). Social Distancing Compliance under COVID-19 Pandemic and Mental Health Impacts: A Population-Based Study. *International Journal of Environmental Research and Public Health*, *17*(18), Article 6692. https://doi.org/10.3390/ijerph17186692	x		
Zhao, Y. Q., An, Y. Y., Tan, X., & Li, X. H. (2020). Mental Health and Its Influencing Factors among Self-Isolating Ordinary Citizens during the Beginning Epidemic of COVID-19. *Journal of Loss & Trauma*, *25*(6-7), 580-593. https://doi.org/10.1080/15325024.2020.1761592	x		
Zhao, Z., Sarkhani, S., Sarkhani, M., & Imani, N. (2022). Effect of a home-based exercise programme on mental health and well-being in children during covid-19 pandemic. *International Journal of Sport and Exercise Psychology*, No Pagination Specified.	x		
Zhu, S., Wu, Y., Zhu, C. Y., Hong, W. C., Yu, Z. X., Chen, Z. K., Chen, Z. L., Jiang, D. G., & Wang, Y. G. (2020). The immediate mental health impacts of the COVID-19 pandemic among people with or without quarantine managements [Letter Research Support, Non-U.S. Gov't]. *Brain, Behavior, & Immunity*, *87*, 56-58.		x	x
Zhuo, K. F., & Zacharias, J. (2021). The impact of out-of-home leisure before quarantine and domestic leisure during quarantine on subjective well-being. *Leisure Studies*, *40*(3), 321-337. https://doi.org/10.1080/02614367.2020.1843693	x		

Znazen, H., Slimani, M., Bragazzi, N. L., & Tod, D. (2021). The Relationship between Cognitive Function, Lifestyle Behaviours and Perception of Stress during the COVID-19 Induced Confinement: Insights from Correlational and Mediation Analyses.	x			
Zoowa, S. B., Shrestha, L., Paudel, L., Bhandari, G., Sapkota, S., & Timilsina, B. (2021). Socio-psychological Study of COVID-19 Pandemic among Healthcare Workers in a Medical College of Nepal: A Descriptive Cross-sectional Study. *Jnma, Journal of the Nepal Medical Association*, *59*(234), 160-164.	x			
Zubair, K., Luqman, M., Ijaz, F., Hafeez, F., & Aftab, R. K. (2020). Practices of General Public Towards Personal Protective Measures During the Coronavirus Pandemic. *Annals of King Edward Medical University Lahore Pakistan*, *26*, 151-156. <Go to ISI>://WOS:000583445800013		x	x	
Zubair, U. (2021). Psychiatric morbidity among patients tested positive and isolated for COVID-19 [Conference Abstract]. *European Psychiatry*, *64(Supplement 1)*, S261.		x		x
