# Supplementary material for: The impact of self-isolation on psychological wellbeing in adults and how to reduce it: A systematic review
Source: PLoS One. 2025 Mar 28;20(3):e0310851. doi: 10.1371/journal.pone.0310851 (PMC11952258; doi:10.1371/journal.pone.0310851)
Supplement: S5 Appendix — (PDF) [file pone.0310851.s005.pdf]

Supplementary materials 5

The impact of self-isolation on psychological wellbeing and how to reduce it: a systematic review

Alex F. Martin[1, 2*], Louise E. Smith[1, 2], Samantha K. Brooks[1, 2], Madeline V. Stein[1], Rachel Davies[1], Richard Amlôt[2, 3], Neil Greenberg[1, 2], G James Rubin[1,2]

[1] King's College London, Institute of Psychiatry, Psychology and Neuroscience, London, UK

[2] NIHR Health Protection Research Unit in Emergency Preparedness and Response, London, UK

[3] UK Health Security Agency, Chief Scientific Officer's Group, UK

Contents

S5 APPENDIX: DATA EXTRACTION TABLES* 2

S5.1 Table. Study characteristics* 2

S5.2 Table. Isolation characteristics* 12

S5.3 Table. Quantitative outcomes by research aim* 16

S5.4 Table. Qualitative outcomes by research aim 27

S5 Appendix: Data extraction tables*

* *Tables note.* Data extraction for each study was conducted by AFM throughout June 2023. All studies included in the extraction tables were deemed eligible for inclusion in the review. The extracted data includes all necessary information to replicate the analyses.

S5.1 Table. Study characteristics*

Citation	Country; dates of data collection	Study design; data collection method.	Sample frame; sampling method; sample size; response rate	Study inclusion criteria	Percent female; age: mean (standard deviation), unless otherwise stated; group size (percentage)
**Aaltonen 2022**	Finland; 12 May to 23 June 2020.	Longitudinal (aim 1), cross sectional (aim 2); telephone interview.	Population based; identified from the register of the infectious diseases control unit in the city of Kerava using random sampling; N=112; 98%.	≥18 years old; Finnish-speaking persons; suspected infection between 19 May and 25 June 2020.	Quarantine group N=43<br>58%; 40.9y (12.1).<br>Self-isolation group N=14<br>36.1y (11.2).<br>Control group (negative PCR, N=523) 58%; 43.5y (14.0).
**Abir 2021**	Bangladesh; 1 to 30 April 2020.	Cross sectional; online survey.	Population based; disseminated through social media and snowball sampling; N=10,609; not reported.	≥18 years old; resident of Bangladesh.	50.4%; The majority (57.0%) of respondents were young adults aged 18 to 27y; na.
**Aloba 2021**	Nigeria; October 2020 to January 2021.	Cross sectional; online survey.	Population based; participants presented to one of two treatment centres in the capital of Nigeria; 'eligible Nigerian adults with COVID-19 were recruited'; N=498; not reported.	≥18 years old; stable internet access required; positive PCR.<br><br>Exclusion criteria: pre-existing mental health disorders or co-morbid medical disorders	47.3%; 41.3y (14.6), range=18-80; na.
**Aslaner 2022**	Turkey; 30 September 2020 to 10 January 2021.	Cross sectional; telephone interview.	Older adults; identified from local government records in Kayseri; N=656 included; 81% of those contacted.	≥65 years old.	51.5%; 69.9y (5.7), range=65-80; na.

**Bonsaksen 2020**	Norway; 8 April to 20 May 2020.	Cross sectional; online survey.	Population based; identified from the Norwegian cross-sectional survey CORONAPOP, which collected data by means of an open web-link disseminated from several institutions, including Oslo hospitals, social media, and national and local newspapers; N=4527; not reported.	≥18 years old; Norwegian citizens	85.4% (of the 4,509 who reported data on gender);<br>18–29y: N=1156 (25.5%)<br>30–39y: N=1220 (26.9%)<br>40–49y: N=931 (20.6%)<br>50–59y: N=766 (16.9%)<br>60–69y: N=354 (7.8%)<br>70y+: N=100 (2.2%); na.
**Chakeri 2020**	Iran; not reported.	Intervention – telenursing counselling every other day for 3 weeks.	Population based; recruited through emergency departments of two hospitals using continuous sampling; N=100; not reported.	Patients* who presented to the emergency department and were given a diagnosis of COVID-19 after a CT scan of their lungs; the physician prescribed home quarantine, medication, and continued treatment at home.<br><br>**Assumed to be adults as participants are described as 'men and women', although age is not specified.*	Control group (N=50)<br>Not reported; 42.4y (9.0)<br><br>Intervention group (N=50)<br>Not reported; 42.7y (9.4).
**Daly 2021**	Canada; 14 to 29 May 2020.	Cross sectional; online survey.	Population based; survey distributed by a national polling vendor, the total panel includes 125,000 adults from all provinces and territories. Increased recruitment of panel participants from traditionally under-represented groups. Participants were randomly sampled using stratification of sociodemographic characterises to ensure representative of Canadian population with adjustments for response propensity; 3558 were invited to reach 3000 (84% response rate).	≥18 years old; resident in Canada.	50.6%;<br>18-34y: N=534 (17.8%)<br>35-54y: N=1157 (38.6%)<br>55y+: N=1309 (43.6%); na.

**Domenghino 2022**	Switzerland; participants enrolled 6 October 2020 to 26 January 2021 (retrospective group) and 6 August 2020 to 19 January 2021 (prospective group).	Qualitative*; free text survey responses.<br><br>**only the qualitative findings met the review inclusion criteria*	Population based; identified from the Zurich SARS-CoV-2 cohort study, individuals with SARS-CoV-2 infection reported to authorities. Two populations were recruited, the first 'retrospectively recruited' (infected prior to the study) and the second 'prospectively recruited', an age-stratified random sample of all eligible individuals; N=1,547;<br>For the retrospectively recruited population: N=442; 33.7%, for the prospectively recruited population: N=1,105; 34.5%.	≥18 years old; residing in the Canton of Zurich; able to follow study procedures; sufficient knowledge of German language; diagnosed with SARS-CoV-2 infection either before the study (between 27 February 2020 and 05 August 2020, retrospective group) or between 06 August 2020 and 19 January 2021 (prospective group).	50.6%; 49.2y (range 17-92)*; na.<br><br>**being 18 or over was one of the inclusion criteria; unclear why 17-year-olds were included*
**Flores-Torres 2021**	Mexico; 4 June to 8 July 2020.	Cross sectional; online survey.	Population based; identified from the Study of Urban Health and Social Distancing (SUSana) of Mexico City government employees through an email invite<br>N=2,016; 14.0%.	All employees with access to an institutional email*<br><br>Exclusion criteria: employees from Mexico City's Ministry of Health.<br><br>**Although not stated as an inclusion criterion, all participants were ≥18 years old.*	49.8%; 42.6y (12.1); na.

**Gok 2022**	Turkey; 'Procedure' subsection of the Methods states data was collected between 1 April to 30 May 2021, however, the 'Sample' subsection of the Methods states that data collection started 'in March 2021 and was completed in about a month' - unclear which of these is correct.	Qualitative; free text survey responses.	Population based; individuals diagnosed with COVID-19 living in Turkey, recruited via 'appropriate sampling' which is the sample of participants who can be 'accessed more effortlessly because of limitations such as time and place'; N=212; not reported.	Living in Turkey; diagnosis of COVID-19.*<br><br>**Although not stated as an inclusion criterion, all participants were ≥18 years old.*	72.2%; 35.8y (9.67)<br>Range 18-60<br>18-25: N=37 (17.4%)<br>26-35: N=71 (33.4%)<br>36-46: N=73 (34.4%)<br>47-60: N=31 (14.8%), na.
**Havlioglu 2022**	Turkey; January – April 2021.	Cross sectional; online questionnaire.	Population based; all individuals in region with a positive PCR; N=800; 28.2%.	≥18-year-old; in the Sanlıurfa province; positive PCR.	45.1%; 18-30 N=539 (44.1%) no other age data reported; na.
**Isherwood 2022**	Wales; Round 1, 11 November to 1 December 2020 and Round 2, 18 February to 23 March 2021.	Cross sectional; telephone interview.	Population based; all individuals who were a close contact of a confirmed case, quota sampling based on age, gender, and SES; N=2,027; 18.8%.	≥18 years old; resident in Wales; successfully contacted by TTP after forward contact tracing; completed their self-isolation period at the time of telephone survey.<br><br>Exclusion criteria: currently self-isolating; a contact of a case of COVID-19 who died.	53.6%;<br>18-29: N=598 (29.5%)<br>30-39: N=374 (18.5%)<br>40-49: N=344 (17.0%)<br>50-59: N=409 (20.0%)<br>60+ N=301 (9.6%); na.

**Jagadeesan 2022**	India; May to June 2021.	Intervention – Bhramari Pranayama (yogic breathing) 20 mins twice daily (online) for 15 days.	Population based; patients registered in a COVID-19 home care programme; N=42 participated; 70.0%.	18-60 years old; under home care program of the host institute; met the diagnostic criteria of COVID-19 and asymptomatic; with an internet facility; able to independently cooperate with doctors in online programme.<br><br>Exclusion criteria: moderate to critical COVID-19 symptoms; pre-existing acute respiratory diseases, asthmatic, heart disease, cognitive impairments, and pregnant women.	30.0%; 39.2y (14.6); na.
**Jang 2022**	South Korea; 16 August to 31 October 2020.	Cross sectional; in person interview.	Population based; identified from the 2020 Korean Community Health Survey (KCHS) using the population stratified sampling from the original study; N=1,071 included; not reported.	≥19 years old; all individuals in KCHS study who had self-isolated.	46.6%; 40.0y (standard error=0.31); na.
**Jesmi 2021**	Iran; April 2020 to July 2020.	Qualitative; in-person and telephone interviews.	Population based; recruited from Sabzevar Vasei Hospital (Iran) after discharge using purposive and snowball sampling; N=14; not reported.	≥18 years old; confirmed diagnosis of COVID-19 by a chest CT scan and PCR test; able to communicate and willing to share their experiences	64%; 37.7y (9.5*); na.<br><br>**calculated by the review authors – the paper only presented individual ages and a range of 25-53*
**Joisten 2022**	Germany; Not reported	Cross-sectional, online survey	Population based; taken from the CoCo-Fakt cohort study, identified through government register of infected people and their close contacts*; N=10,490; 31.1%.<br><br>**data from the same study as Wessely et al. (2022), but reporting different outcomes.*	>16 years of age*<br><br>Exclusion criteria: without an email address, hospitalised or deceased affected, nursing home residents.<br><br>**16 and 17 year olds were included, although the number was not reported, the mean age and SD suggests that they were a small minority and the study was therefore included*	Whole sample: 60.0%, 40.8y (14.2);<br>Infected N=4,065<br>Contacts N=6,425.
**Ju 2021**	China; 10 February to 2 April 2020.	Longitudinal, prospective; online questionnaire.	Population based; recruited from The First Hospital of Changsha (Hunan, China) after discharge; N=146 included at baseline (79.8%)	≥18 years old (one adolescent aged 15 was included); able to use mobile devices to complete the questionnaires.	Whole sample: 46.3%; median = 39y (IQ range 30-47); of 95 included in the study (that completed both timepoints)<br>Home isolation N=45<br>Hotel isolation N=50.

**Kopilas 2021**	Croatia and Italy; 4 to 24 March 2020	Cross sectional*; online questionnaire.<br><br>**only the quantitative findings met the review inclusion criteria*	Population based; convenience sampling of universities and colleagues; N=164; 71.3%.	≥18 years old; speak English.	Whole sample: 69.3%; 37.3y (SD=13.6);<br>Exposed (isolation following close contact) N=27.<br>Not exposed N=136 (made up of three groups: in lockdown (N=72), no contact (N=21) and unrelated (N=43)).
**Kowalski 2021**	Germany; 29 January to 12 April.	Cross sectional; online survey.	Population based; taken from government register of positive PCR cases; N=224 included; 37.5%.	≥18-year-old; within the jurisdiction of the Freudenstadt Health Department; all individuals with a positive PCR.<br><br>Exclusion criteria: in inpatient care facilities; language barrier to questionnaire completion.	Whole sample: 52.7%; 41y (14.6);<br>With psychological burden, N=104 without N=121<br>Without psychological burden N=104.
**Lohiniva 2021**	Finland; April to May 2020.	Qualitative; in-person interviews.	Population based; participants were PCR confirmed cases of COVID-19 (and their families) recruited via the Finnish Institute for Health and Welfare website or an SMS sent to confirmed cases, sampling was based on 'maximum variation' to engage participants from different types of households – this study is part of a larger study on COVID-19 transmission where individuals recruited for that study could also take part in this qualitative study if they wished;<br>N=64 participants from 24 households; not reported.	≥12 years old*; households located in Helsinki; households with at least one COVID-19 PCR confirmed case and at least one additional person in the household, 'in home quarantine or in isolation for a period of time'.<br><br>**only those participants 18+ have had their data extracted due to this review's inclusion criteria*	Of the 50 adults: gender % not reported ('the sample included approximately an equal number of female and male respondents'); mean age not reported, 'most adult participants were in the age range of 30-49 (75%)'; na.
**Maric 2022**	Serbia, June to October 2021.	Cross sectional; in-person interviews.	Population based; identified from the Serbian CoV2Soul.rs study using multistage probabilistic sampling from 135 randomly selected local communities in 60 out of the 180 municipalities in Serbia and quasi-randomisation of households in identified areas; N=1,203, 67.0%.	18-65 years old; resident in identified households; spoke Serbian.	51.3%; 43.7y (13.6); na.

**Mohamed 2021**	Egypt, 22 May to 28 July 2020.	Cross sectional; online survey.	Population based. Patients were recruited from Zagazig University Hospital; N=89; not reported.	≥18 years old; PCR confirmed mild and moderate cases of COVID-19.<br><br>Exclusion criteria: Presence of 'mental retardation, dementia, or delirium'; confirmed severe cases of COVID-19.	Home isolated N=43; 33.6%; 39.9y (8.8) Hospital isolated (N=46); 30.4%; 41.3y (9.3).
**Navas 2022**	Spain, April to June 2020.	Cross sectional; telephone interview.	Population based; selected from primary care electronic medical records lists from the Passeig de Sant Joan Primary Care Team in Barcelona; N=89; not reported.	≥18 years old; PCR confirmed infection; have lived with other people in the preceding two weeks.<br><br>To be eligible to stay in the supervised hotel, they had to fulfil at least one of the following criteria: caring for a vulnerable person; being destitute or homeless; being a tourist in transit; sharing their home with many people; fulfilling other vulnerability requirements as assessed by a social worker.	Whole sample: 64%; 53.6y (16.9);<br>Home isolated N=45<br>Hotel isolated N=44.
**Oginni 2021**	Nigeria; not reported.	Cross sectional; online survey.	Population based; recruited through social media and WhatsApp, participants represented all Nigerian tribes; N=966; not reported.	≥18 years old; resident in Nigeria for at least six months prior to the lockdown; fluent in English; able to use the internet;<br><br>Exclusion criteria: severe cognitive or physical impairments.	49.6%; median = 27y (IQR=12y); na.
**Opakunle, 2022**	Nigeria; October 2020 to February 2021.	Cross-sectional; online survey.	Population based; convenience sampling via email and WhatsApp; N=509; not reported.	18 to 80 years old; no pre-existing mental disorder or co-morbid medical problems.<br><br>Exclusion criteria: too physically ill during isolation; admitted to hospital or isolation centre; given oxygen therapy.	47.3%; 41.2y (14.6); na.
**Paz 2020**	Ecuador; 22 March to 18 April 2020.	Cross-sectional; online survey.	Population based; taken from the surveillance program for COVID-19 established by the Ecuadorian Ministry of Public Health, a non-probabilistic sampling strategy was used from the beginning of the study until quota was reached; N=759; 88.7% response rate.	≥18 years old; living in Ecuador; under surveillance for diagnosis or suspected illness.	51.9%; 37.7y (11.0) range 18-94; na.

**Petrocchi 2021**	Switzerland; COVID-19 positive (groups 1&2), 4 August to 5 October 2020. COVID-19 negative (group 3), 8 September to 15 October 2020.	Cross sectional; online survey or paper survey (optional).	Population based; random sampling to produce several groups with different sampling methods:<br>COVID-19 positive groups: hospital database of COVID-19 cases<br>1. Home isolated; N=63; 12.6%<br>2. Hospital isolated; N=76; 24.5%<br>3. COVID-19 negative group: university Facebook advert<br>N=61; 31.3%.	≥18 years old.	Home isolated group N=63<br>60.3%; 41.9y (14.3) range=19–75<br><br>Hospital isolated group N=76<br>26.3%; 62.1y (14.8) range=24-87<br><br>Covid negative group N=61<br>83.6%; 41.3y (13.6) range=19–74.
**Pheh 2020**	Malaysia; not reported.	Intervention*; online survey<br><br>**this study reports an RCT during nationwide stay at home orders. Here, data is reported related to aim 1 and data is used as a longitudinal, prospective design.*	Population based; convenience and snowball sampling using authors' social media; N=161; 68.5%.	Not reported.*<br><br>**Although not stated as an inclusion criterion, all participants were ≥18 years old.*	73.9%; 28.8y (9.0) range=18-70; Experienced self-isolation N=16 (9.9%)
**Plesea-Condratovici 2022**	Romania; not reported.	Cross sectional; telephone interview.	Population based; recruited from GP monitoring of COVID-19 positive patients from two GP settings; N=107; not reported.	Not reported.*<br><br>**13-18 year olds were included, although the number was not reported. We calculated, using the mean age and SD, that those <18y were a small minority (2 SDs of the sample gives an estimated age range of 15-76y) and the study was therefore included.*	42%; 45.1y (15.2) range 13-83; na.
**Rajagopalan 2022**	India; not reported.	Intervention: OM chanting and meditation 20 minutes twice a day for 14 days.	Population based; recruited from the home care program of Saveetha medical college and hospital; N=25 included; 28.4%.	18-60 years old; asymptomatic COVID-19 patients with mild severity.<br><br>Exclusion criteria: pregnant women; moderate or severe symptoms; other comorbid conditions.	40.0%; range 41-60 years; na.

**Ripon 2020**	Bangladesh; 10 to 29 May 2020.	Cross sectional; online survey.	Population based; social media; N=579; not reported.	≥18 years old, Bangladeshi citizens; diagnosis by PCR.<br><br>Exclusion criteria: did not complete the 10th grade or below of education; without a job.	Not reported;<br>18-30y: 17.3%<br>31-45y: 32.5%<br>46-55y: 30.1%<br>>55y: 19.8%.<br><br>Home quarantine N=3952 (68.2%)<br>Institutional quarantine N=1840 (31.8%)
**Rossi 2020**	Italy; 27 March to 6 April 2020.	Cross sectional; online survey.	Population based; paid social media advert plus snowballing; N=18,146; not reported.	≥18 years old; Italian citizens.	79.5%; median = 38y (IQR=23y); C<br>Currently in quarantine N=141 (0.8%).
**Schluter 2022**	8 countries and territories: Canada, USA, England, Switzerland, Belgium, Philippines, New Zealand and Hong Kong; 6 to 18 November 2020.	Cross sectional; online survey.	Population based; representative quota sampling for each country (age, gender, region), collected by two polling forms using telephone, online and offline recruitment;<br>N=9027; not reported.	≥18 years old; resident in one of eight included countries.	52% (0.5% did not identify with male or female); 47.0y (17.0) range=18-99;<br>No isolation N=5753<br>COVID contact N=566<br>COVID symptoms N=720<br>COVID diagnosis N=457<br>Travel/health N=1199.
**Verberk 2021**	Netherlands (N=10) and Belgium (N=8); 5 May to 9 July 2020.	Qualitative*; telephone interview.<br><br>**only the qualitative findings met the review inclusion criteria*	Population based; for the overall study, patients with confirmed positive tests in the Netherlands or Belgium - identified via drive-through testing sites, healthcare worker screening, hospital emergency visits, primary care physicians or preoperative screening - received a flyer; 22 households; 81.8% response rate).	≥18 years old; living with someone with a confirmed positive test of COVID-19.	55.6%; 43y (range 25-77); na.

**Wessely 2022**	Germany; 12 December 2020 to 6 January 2021.	Cross sectional; online survey.	Population based; taken from the CoCo-Fakt cohort study, identified through government register of infected people and their close contacts*, N=8,075 included; 22.7%.<br><br>**data from the same study as Joisten et al. (2022), but reporting different outcomes.*	>16 years of age*<br><br>Exclusion criteria: noncompliant people; deceased patients; those who were in medical or nursing facilities or quarantined for other reasons (e.g., travel returnees)<br><br>**16 and 17 year olds were included, although the number was not reported, the mean age and SD suggests that they were a small minority and the study was therefore included*	61.5%; 41.6y (14.2);<br>Infected N=3,208<br>Contacts N=4,867.
**Xu 2020**	China; 8 to 21 February 2020.	Cross sectional; online survey.	Population based; survey was 'posted on the Internet' using convenience sampling; N=328; not reported.	≥18 years old; resident in mainland China.	39.0%; 31.0y (6.8) range=18-56; na.

S5.2 Table. Isolation characteristics*

Citation	Reason for isolation/quarantine (infected, suspected to be infected, close contact)	Location (make this home if stated, assumed to be home if not); duration of isolation/quarantine; lockdown context	Assessment points; amount of time between isolation/quarantine and data collection
**Aaltonen 2022**	Infected, close contact.	Home; the mean quarantine duration for the quarantined sample was 10.8 days (SD=3.4); not reported.	Baseline = cases were telephone interviewed about at 1 week (baseline) since the onset of the quarantine and the controls within 10 days since PCR testing.<br>Follow up = interviews were conducted mean 15.6 [SD 2.4] days since the onset of quarantine and mean 4.9 [SD 3.5] days since the expiration.
**Abir 2021**	Infected, close contact.	Assumed to be home; not reported; mandatory lockdown was in place in parts of Bangladesh, before the vaccine rollout.	'currently in self-quarantine since past 7 days'
**Aloba 2021**	Infected.	Home; mean duration = 8.79 days (SD=2.71) range=1-14; not reported.	Participants given a code for the online survey at the treatment centre, days before completing survey not reported
**Aslaner 2022**	Infected, suspected to be infected, close contact.	Home; not reported; not reported.	Not reported
**Bonsaksen 2020**	Infected, close contact.	Assumed to be home; not reported; Norwegian authorities imposed a lockdown of society on 12 March 2020. While restrictions were lifted gradually during the following months, people were encouraged to shelter in place. Kindergartens, schools, and universities were closed, as were non-vital businesses requiring physical proximity, and cultural events and travels were cancelled.	Not reported: 'have been in quarantine/isolation'
**Chakeri 2020**	Infected.	Home; not reported; not reported.	Pre-intervention = the start of isolation (day 0)<br>Follow up = day 21
**Daly 2021**	Infected, suspected to be infected, close contact.	Assumed to be home; 10-14 days; Data collection was approximately one month after the initial peak of the COVID-19 pandemic in Canada and coincides with the time during which many jurisdictions began their initial phases of "re-opening", following approximately two months of widespread restrictions.	1 assessment point; retrospectively reported 'since the start of the pandemic', which was ~6-8 weeks before data collection.

Citation	Reason for isolation/quarantine (infected, suspected to be infected, close contact)	Location (make this home if stated, assumed to be home if not); duration of isolation/quarantine; lockdown context	Assessment points; amount of time between isolation/quarantine and data collection
**Domenghino 2022**	Infected.	For the whole sample: 92.8% at home, 2.1% at someone else's home, 4.9% in hospital, 0.2% at a social institution, 0.1% in a hotel, 2.1% 'other', 1.3% missing data. Additionally, 55 participants isolated in more than one place, in most cases (N=41) first at hospital and then at home; Retrospectively recruited group: median number of days in isolation 10.0, range 2.0-25.0 (N=276, 25%, had missing data for this), prospective group: not reported; not reported.	Retrospective group completed baseline measures a median of 7.2 months after diagnosis; prospective group completed baseline measures 'upon or shortly after diagnosis' Both groups completed follow-up measures two weeks and four weeks after baseline
**Flores-Torres 2021**	Suspected to be infected , close contact.	Assumed to be home; not reported; during national stay-at-home directives.	Self-isolation in the past week
**Gok 2022**	Infected.	Home; not reported; not reported.	Not reported
**Havlioglu 2022**	Infected.	Home; 14 days; 'Some of the measures ... not leaving the house except for necessities, closing schools, and telecommuting in public offices'.	Not reported
**Isherwood 2022**	Close contact.	Home; 10 or 14 days; not reported.	Not reported
**Jagadeesan 2022**	Infected.	Home; 15 days; not reported.	Pre-intervention: start of isolation (day 0) Post-intervention: end of isolation (day 16)
**Jang 2022**	Infected, close contact.	Assumed to be at home; not reported; not reported.	Not reported
**Jesmi 2021**	Infected.	Assumed to be at home; not reported; not reported.	Not reported
**Joisten 2022**	Infected, close contact.	Home; mean = 11.8 days, SD = 4.6 days; not reported.	Not reported
**Ju 2021**	Infected.	Home vs hotel; two weeks; not reported.	Baseline = discharge day Follow up = two weeks after discharge, when isolation complete, days before completing the survey not reported
**Kopilas 2021**	Close contact.	Home; two weeks; lockdown and non-lockdown contexts.	During the isolation period
**Kowalski 2021**	Infected.	Home; 14 days; not reported.	During isolation (M=12.7 days, SD=4.6)
**Lohiniva 2021**	Infected, close contact.	Home; not reported; not reported.	Interviews were all conducted at least 28 days after the household index's PCR confirmation; PCR confirmed cases occurred during February-April 2020 and interviews took place during April – May 2020
**Maric 2022**	Close contact.	Assumed to be at home; not reported; between pandemic peaks, limited restrictions in place and substantial vaccination rates.	During interview; not reported

Citation	Reason for isolation/quarantine (infected, suspected to be infected, close contact)	Location (make this home if stated, assumed to be home if not); duration of isolation/quarantine; lockdown context	Assessment points; amount of time between isolation/quarantine and data collection
**Mohamed 2021**	Infected.	Home vs hospital; not reported; not reported	Not reported
**Navas 2022**	Infected.	Home vs hotel; 14 days; during data collection, Spain was the country with the second highest number of infected people and the third in number of deaths. A number of restrictive measures had been in place for two months.	One assessment point; during isolation treatment
**Oginni 2021**	Suspected to be infected.	Assumed to be home; not reported; during national lockdown.	Not reported
**Opakunle, 2022**	Infected.	Home; not reported; not reported.	Not reported; Ct values taken from PCR test at presentation to treatment centre
**Paz 2020**	Infected, suspected to be infected.	Assumed to be at home; not reported; not reported	Not reported
**Petrocchi 2021**	Infected.	Home vs hospital; not reported; during national lockdown	Quarantine during 22 March to 11 May 2020; 5-7 months before data collection
**Pheh 2020**	Not reported.	Home; not reported; during nationwide movement control order (i.e. lockdown).	Baseline (at recruitment into study)<br>Post treatment (same day as intervention)<br>Follow-up (2 weeks after the end of treatment)
**Plesea-Condratovici 2022**	Infected.	Home; 14 days; not reported	GP daily phone monitoring during isolation
**Rajagopalan 2022**	Infected.	Home; 14 days; not reported	Pre-intervention – start of isolation (day 0)<br>Post-intervention – end of isolation (day 15)
**Ripon 2020**	Infected.	Home vs instituition; 14 days; National lockdown had been extended to cover the data collection period	Within 5 days of end of quarantine
**Rossi 2020**	Infected, close contact.	Assumed to be home; not reported; during nationwide lockdown 'included travel restrictions, the mandatory closure of schools, nonessential commercial activities and industries. People were asked to stay at home and socially isolate themselves to prevent being infected' and 'the investigated timeframe corresponds to the contagion peak in Italy, according to epidemiological data confirmed by the World Health Organization'.	Currently in quarantine (i.e. self-isolation)
**Schluter 2022**	Close contact, suspected to be infected, infected, travel/health.	Assumed to be home; not reported; 1 year after first reported case, 'Selection of countries for inclusion was based on ensuring global continent diversity and … capturing different demographics, health systems and policies, and COVID-19 burdens and response'.	Not reported; within 12 months following the earliest reported COVID-19 case.

Citation	Reason for isolation/quarantine (infected, suspected to be infected, close contact)	Location (make this home if stated, assumed to be home if not); duration of isolation/quarantine; lockdown context	Assessment points; amount of time between isolation/quarantine and data collection
**Verberk 2021**	Close contact, infected.	Home; 10-14 days (mean=11.8 days, SD=4.6); not reported.	1 assessment point; up to 11 months
**Wessely 2022**	Close contact.	Home; at least 7 days and 24 hours symptom free; not reported.	7-15 days after the COVID-19 diagnosis of the index case
**Xu 2020**	Close contact.	Home; not reported; not reported.	1 assessment point, during quarantine

S5.3 Table. Quantitative outcomes by research aim*

Citation	Measures of psychological wellbeing	Impact of self-isolation on wellbeing: Test (relationship analysed) *bold indicates significance *italicised indicates predictor (isolation type)*	Factors associated with wellbeing:<br><br>Test (relationship analysed) *bold indicates significance *italicised indicates wellbeing outcome*	Intervention outcome on wellbeing<br><br>Test (relationship analysed) *bold indicates significance *italicised indicates wellbeing outcome*
**Aaltonen et al., 2022**	CORE-OM Psychic wellbeing and distress symptom scores and subscales<br><br>Patient Health Questionnaire (PHQ-9; depression symptoms)<br><br>Overall Anxiety Severity and Impairment Scale (OASIS; anxiety symptoms)<br><br>Binarised using established thresholds indicating a clinical diagnosis or severity classification	Multivariate linear regression<br>Overall psychological wellbeing symptom score at baseline (7-10 days after PCR/start of quarantine) in the whole sample (*being quarantined was not significantly associated*)<br><br>Kruskal-Wallis one way ANOVA CORE-OM – Subjective wellbeing<br>CORE-OM – Problems/symptoms<br>**CORE-OM – Life functioning (*all quarantined/isolating > controls*)**<br>CORE-OM – Risk/harm<br>Chi-square test (unadjusted)<br>CORE-OM – Distress<br><br>**only adjusted estimates are reported in the summary table (Aim 1 ROB relates to the adjusted analysis)*	Chi square tests (unadjusted) comparing reason for quarantine (infected vs close contacts) at 2-week follow-up<br>*Overall psychological wellbeing clinical outcome*<br>***Depression clinical outcome* (infected > close contac**t)<br>*Anxiety clinical outcome*<br><br>Kruskal-Wallis one way ANOVA (unadjusted) comparing reason for quarantine (infected vs close contacts) at follow-up)<br>CORE-OM – Total score<br>CORE-OM – Subjective wellbeing<br>CORE-OM – Problems/symptoms<br>CORE-OM – Life functioning<br>CORE-OM – Risk/harm<br>Chi-square test (unadjusted)<br>CORE-OM – Distress	Not assessed
**Abir et al., 2021**	Impact of Event Scale Revised (IES-R, psychological impact)<br><br>Binarised: mild to severe vs none	Logistic regression (adjusted odds ratio)<br><br>**Psychological impact (*quarantine > no quarantine*)**	Not reported	Not reported
**Aloba & Opakunle, 2021**	Wellbeing outcome: Insomnia Severity Index (ISI)<br><br>Predictors:	Not reported	Multivariate linear regression:<br><br>*Insomnia:*<br>Age	Not reported

	Generalized Anxiety Disorder-7 scale (GAD-7; anxiety symptoms)<br>Patient Health Questionnaire (PHQ-8; depression symptoms)<br>Suicidal ideation (item 9 of the PHQ-9)<br>Brief Symptoms Rating Scale (BSRS-5; general psychological symptoms).		Viral load<br>Anxiety<br>Depression<br>**General psychological symptoms (positive association)**<br>**Suicidal ideation (positive association)**<br>**Duration of isolation (negative association)**	
**Aslaner et al., 2022**	Coronavirus Anxiety Scale (CAS)<br><br>Death Anxiety Scale (DAS), how categorised into y/n is not reported	Not reported	Logistic regression (adjusted odds ratio)<br>*Death anxiety*<br>**Gender (male > female)**<br>**Positive PCR (yes > no)**<br>**Presence of symptoms (no > yes)**	Not reported
**Bonsaksen et al., 2020**	PTSD Checklist (PCL-5; PTSD) to measure symptoms, binarised using DSM-5 diagnostic guidelenes to categorise y/n	Logistic regression (adjusted odds ratios)<br><br>**PTSD (*been in quarantine/isolation > not been in quarantine/isolation*)**	Not reported	Not reported
**Chakeri et al., 2020**	The Iranian version of the Spielberger Anxiety Inventory (SAI; anxiety symptoms)	Not reported	Not reported	Independent t-test<br><br>*Anxiety symptoms following the intervention* **(pre > post)**<br><br>*Anxiety symptoms at follow up* **(Controls > intervention)**
**Daly et al., 2021**	Subjective report: 'compared to before the COVID-19 pandemic, how would you say your mental health is now?' 5 point likert scale, slightly and significantly worse were collapsed into outcome 'worse mental health'<br><br>Suicidal thoughts and self harm, separate items: 'as a result of the COVID-19 pandemic, in the previous two weeks, have you experienced either suicidal thoughts/feelings' or 'deliberately hurt yourself'	Chi-square test:<br>**Rates of subjective worse mental health (*isolated for any reason (EXCLUDING isolate for travel) > those who had not isolated*)**	Not reported	Not reported

**Flores-Torres et al., 2021**	The Spanish version of the 7-item Center for Epidemiologic Studies Depression scale (CESD-7), categorised to be clinically significant y/n<br><br>The Spanish version of the Generalized Anxiety Disorder-7 scale (GAD-7), categorised to be clinically significant y/n<br><br>Subjective item: has worry or stress related to the coronavirus had a negative impact on your mental health? (binarised y/n)	Logistic regressions (adjusted odds ratio)<br><br>**depression (*self-isolated in past week > not self-isolated in past week*)**<br>anxiety<br>mental health impact	Not reported	Not reported
**Havlioglu et al., 2022**	The Turkish version of the Padua Inventory-Revised (Padua; Obsessive-compulsive symptoms)	Not reported	Individual ANOVAs and post hoc Tukey tests for pairwise group differences<br><br>*Obsessive compulsive symptoms:*<br>Gender<br>Age<br>**Education (degree > high school/elementary school)**<br>SES<br>**Comorbid disease (yes > no)**<br>Time of year (season)<br>**Psychiatric illness (yes > no)**	Not reported
**Isherwood et al., 2022**	Subjective self-reported mental health difficulties (one item) and loneliness (one item)	Not reported	Logistic regression (adjusted odds ratios)<br><br>*Mental health difficulties*<br>**Age (younger > older)**<br>**Gender (female > male)**<br>Living alone<br><br>*Loneliness*<br>**Age (younger > older)**<br>Gender<br>**Living alone (no > yes)**	Not reported
**Jagadeesan et al., 2022**	Depression Anxiety and Stress Scale-21 (DASS-21; total symptom score,	Not reported	Not reported	Paired t-test (unadjusted)

	depression score, anxiety score, stress score)<br><br>Pittsburgh Sleep Quality Index (PSQI; sleep quality)<br><br>WHO Quality of Life (WHOQ OL-BREF; psychological subscale)			***Total symptoms* (baseline > follow up)**<br>***Depression* (baseline > follow up)**<br>***Anxiety* (baseline > follow up)**<br>***Stress* (baseline > follow up)**<br>***Insomnia* (baseline > follow up)**<br>***Quality of life* (follow up > baseline)**
**Jang et al., 2022**	Patient Health Questionnaire-2 (PHQ-2; depression symptoms) binarised 'subject had depressive symptoms' y/n	Not reported	One logistic regression model (adjusted odds ratios)<br><br>*Depression symptoms:*<br>**Age (<40y > 40-64y and 65y+)**<br>Gender<br>**Education (<middle school > college)**<br>**Income (highest > all lower categories)**<br>**Changes in daily life due to COVID-19 (no > yes)**<br>Employment<br>**Poor physical health (yes > no)**<br>Married<br>Living alone<br>**Assistance with isolation (no > yes)**<br>**Counselling for depression (yes > no)**	Not reported
**Joisten et al, 2022**	Psychological stress<br><br>Five items adapted from the COVID-19 Snapshot Monitoring (COSMO) questionnaire from the University of Erfurt<br><br>In detail, item 1 from the generalized anxiety disorder (GAD-7) items 6, 8 and 14 from the Allgemeine Depressionsskala (ADS) and item 19	Not reported	Test not reported.<br><br>**Infected > close contact**	Not reported

	from the IES-R24 (impact of event scale). *<br><br>**The review authors retrieved this assessment from the study protocol*			
**Ju et al., 2021**	Chinese version of the 9-item Patient Health Questionnaire (PHQ-9; depression symptoms)<br><br>Chinese version of the 7-item Generalized Anxiety Disorder scale (GAD-7; anxiety symptoms)	Not reported	General linear model with repeated measures to examine the effects of time by isolation type on depression, anxiety. Covariates (demographics, disease-related, counselling during isolation) did not predict the outcome in the mulitivariate model and were removed from the final model.<br><br>*Depression symptoms:*<br>**Time (baseline > follow up)**<br><br>**Interaction time x isolation type**.<br>Post hoc analysis showed a **decrease of depression scores in the home group but not in the hotel group**.<br><br>*Anxiety symptoms:*<br>**Time (baseline > follow up)**<br><br>**Interaction time x isolation type**.<br>Post hoc analysis showed a **decrease of anxiety scores in the home group but not in the hotel group.**	Not reported
**Kopilas et al., 2021**	Depression Anxiety Stress Scale-21 (DASS-21; depression and anxiety)<br><br>Impact of Event Scale-Revised (IES-R; distress subscales)<br><br>Positive and Negative Affect Schedule (PANAS; affect)<br><br>UCLA Loneliness Scale (ULS; loneliness)	One-way MANCOVA to examine effect of isolation type with post hoc tests for group differences.<br><br>*Isolation (exposed) compared to not in isolation (unexposed):*<br>Depression symptoms<br>Anxiety symptoms<br>Stress symptoms<br>Distress: Intrusion	Not reported	Not reported

		**Distress: Hyperarousal (*isolation* < *not in isolation*)**<br>Distress: Avoidance<br>Positive affect<br>Negative affect<br>Loneliness		
**Kowalski et al., 2021**	Psychological burden = reaching at least one of the cut-off scores of PHQ-8, GAD-7, or SSD-12:<br><br>Patient Health Questionnaire 8 (PHQ-8; depression).<br><br>Generalized Anxiety Disorder Scale 7 (GAD-7; anxiety).<br><br>Somatic Symptom Disorder-B Criteria Scale (SSD-12; distress).	Not reported	Mann-Whitney U-test (unadjusted),<br><br>*Psychological burden*<br>**Perceived stress (high > low)**<br>**Covid symptoms (high > low)**<br>**Poor physical health (high > low)**<br>**Perceived stressors (disgrace, job fear, social restrictions, infectiousness: (high > low)**<br><br>**Coping strategies (in general and in relation to COVID-19; low > high)**<br>**Social support (high > low)**	Not reported
**Maric et al., 2022 [21]**	Mini International Neuropsychiatric Interview (MINI Standard 7.0.2; mood disorder, anxiety disorder, substance use disorder).<br><br>Patient Health Questionnaire-9 (PHQ-9; depression and anxiety symptoms).<br><br>General Anxiety Disorder-7 (GAD-7: anxiety symptoms)<br><br>Binarised using established cutoffs of symptom intensity	Logistic regression (odds ratios)<br><br>Any disorder<br>Mood disorder<br>Anxiety disorder<br>Substance use disorder	Not reported	Not reported
**Mohamed & Yousef, 2021**	Arabic version of the Hospital Anxiety and Depression Scale (HADS; depression symptoms and anxiety syptoms)		Binary logistic regression (adjusted odds ratios)<br><br>*Anxiety symptoms*<br><br>***Depression symptoms* (home > hospital)**	Not reported

	Arabic version of the Davidson Trauma Scale (DTS; PTSD symptoms).		***PTSD symptoms* (home < hospital)**	
**Navas et al., 2022**	Impact of Events Scale – Revised (IES-R), adapted items (not reported) to create:<br><br>Frustration<br>Anger/irritability<br>Feeling isolated<br>Anxiety<br><br>Rated on a scale of 0 to 5. The variables were coded as 'present' if it had been given a score of 2 to 5 (sometimes, often, almost always or always).	Not reported	Binary logistic regression (adjusted odds ratio):<br><br>***Frustration* (home > hotel)**<br>***Anger/irritability* (home > hotel)**	Not reported
**Oginni et al., 2021**	14-item Hospital and Anxiety Scale (HADS; depression and anxiety subscales)	Multivariate linear regression<br><br>Depression symptoms (in both men and women; *isolation yes* |*no*)<br>Anxiety symptoms (in both men and women; *isolation yes* |*no*)	Multivariate linear regression<br><br>*Depression symptoms* (men | women)<br>*Anxiety symptoms* (men | women)	Not reported
**Opakunle, 2022**	Generalized Anxiety Disorder (GAD-7; anxiety symptoms)<br><br>Patient Health Questionnaire (PHQ-9; depression symptoms)<br><br>1 item from PHQ-9 was used to assess suicidal ideation<br><br>Insomnia Severity Index (ISI-7; insomnia)<br><br>Brief Self Rating Scale (BSRS-5; overall psychological symptoms)<br><br>All categorised using predeterminded thresholds	Not reported	Binary logistic regressions (adjusted odds ratios):<br><br>***Psychological symptoms* (high viral load > low viral load)**<br>***Suicidal ideation* (high viral load > low viral load)**<br>*Anxiety*<br>*Depression*<br>*Insomnia*	

**Paz et al., 2020**	Patient Health Questionnaire (PHQ-9; depression symptoms)<br><br>Generalized Anxiety Disorder (GAD-7; anxiety symptoms)<br><br>Binarised using established cutoff scores indicating severity	Not reported	Logistic regression (adjusted odds)<br><br>*Depression*:<br>**Gender (female > male)**<br>**Region (coastal > other)**<br>**Regular schedule (no > yes)**<br>**Regular exercise (no > yes)**<br>**Time spent on COVID-19 information (1h < none; 1 < more than 1 h)**<br><br>*Anxiety:*<br>**Gender (female > men)**<br>Region<br>**Regular schedule (no > yes)**<br>Regular exercise<br>**Time spent on COVID-19 information (1h < none; 1 < more than 1 h)**	Not reported
**Petrocchi et al., 2021**	The Italian version of the NCCN Distress Thermometer (distress)<br><br>The Generalized Anxiety Disorder 7-item Scale (GAD-7; anxiety symptoms)<br>The Patient Health Questionnaire-9 (PHQ-9; depression symptoms).<br><br>For GAD-7 and PHQ-9, participants indicated how often they had been troubled during lockdown by each symptom, using a four-point Likert scale ranging from 0 (“Not at all”) to 3 (“Nearly every day”).	Not reported	ANOVAs (separate unadjusted models for each outcome) and post hoc Duncan tests for group comparisons<br><br>*Depression* **(COVID pos home > COVID neg home > COVID neg home)**<br><br>*Anxiety* **(COVID pos hospital > COVID neg home)**<br><br>*Distress* (**COVID pos hospital > COVID neg home**)	Not reported
**Pheh et al., 2020**	Subjective Unit of Distress Scale (SUDS; distress)<br><br>The Generalized Anxiety Disorder 7-item Scale (GAD-7; anxiety symptoms)	MANCOVA to examine effect of self-isolation measured at baseline (yes/no) on psychological wellbeing outcomes at follow-up<br><br>Distress<br>**Anxiety (self-isolation > no self-isolation)**	Not reported	Not reported*<br><br>**did not meet inclusion criteria for this review*

	World Health Organization-Five Well-Being Index (WHO-5; general wellbeing)	General wellbeing		
**Plesea-Condratovici et al., 2022**	The Hospital Anxiety and Depression Scale (HADS; anxiety symptoms)	Not reported	Spearman's correlations (unadjusted)<br><br>*Anxiety*<br>**Gender (female > male)**<br>**Rural setting (yes < no)**<br>**Garden (yes < no)**<br>**Preexisting diagnosis of anxiety (yes > no)**<br>**No pulse oximeter (yes > no)**	Not reported
**Rajagopalan et al., 2022**	Depression Anxiety and Stress Scale-21 (DASS-21; total symptom score, depression score, anxiety score, stress score)<br><br>Pittsburgh Sleep Quality Index (PSQI; sleep quality)<br><br>WHO Quality of Life (WHOQOL-BREF; psychological subscale)	Not reported	Not reported	Paired t-test (unadjusted)<br><br>**Total symptoms (baseline > follow up)**<br>**Depression (baseline > follow up)**<br>Anxiety<br>Stress<br>**Insomnia (follow up > baseline)**<br>**Quality of life (follow up > baseline)**
**Ripon et al., 2020**	Impact of Event Scale-Revised (IES-R; PTSD scores ≥20)<br><br>Center for Epidemiologic Studies–Depression scale (CES-D; Depression diagnosis scores ≥16)	Not reported	Binary logistic regressions (adjusted odds ratios)<br><br>*Depression*<br><br>***PTSD* (institutional > home)**	Not reported

**Rossi et al., 2020**	The Global Psychotrauma Screen, post-traumatic stress symptoms subscale (GPS-PTSS; PTSS = 3/5)<br><br>The 9-item Patient Health Questionnaire (PHQ-9; severe depression symptoms ≥15)<br><br>The 7-item Generalized Anxiety Disorder scale (GAD-7; severe anxiety symptoms ≥15)<br><br>The 7-item Insomnia Severity Index (ISI; severe insomnia ≥22)<br><br>The 10-item Perceived Stress Scale (PSS; stress = higher quartile)<br><br>The International Adjustment Disorder Questionnaire (IADQ; adjustment disorder symptoms = if associated with COVID-19)	Binary logistic regression (adjusted odds ratios)<br><br>**PTSS (*currently in quarantine > not in quarantine*)**<br>Depression<br>**Anxiety (*currently in quarantine > not in quarantine*)**<br>Insomnia<br>Stress<br>**Adjustment disorder symptoms (*currently in quarantine > not in quarantine*)**	Not reported	Not reported
**Schluter et al., 2022**	Generalized anxiety disorder (GAD-7; anxiety disorder ≥10)<br><br>Patient Health Questionnaire-9 (PHQ-9; major depressive episode ≥10)	Multivariate multilevel mixed-effects Poisson regression model (adjusted relative risk). No isolation as reference category.<br><br>**Probable anxiety disorder and/or depression disorder (*each quarantine/isolation group (contact, symptoms, diagnosis) > no isolation)***	Pairwise comparisons (adjusted)<br><br>*Probable GAD and/or MDE* no significant difference identified between the isolation groups (contact, symptoms, diagnosis, travel)	Not reported
**Wessely et al., 2022**	Wellbeing outcome: Alcohol use disorder identification test-consumption (AU-DIT-C)<br><br>Predictors:<br>Psychological burden – 5 items taken from the COVID-19 Snapshot Monitoring (COSMO) study	Not reported	Logistic regression. (adjusted odds ratios)<br><br>*Increased alcohol consumption since before the pandemic*<br>**Isolation group (contacts > infected)**<br>**In partnership (yes > no)**<br>**At risk drinking behaviour (yes > no)**<br>**Coping strategies (no > yes)**<br>Age	Not reported

	Coping strategies – 6 items taken from the COSMO study		Gender<br>Education<br>Psychological burden	
**Xu et al., 2020**	Wellbeing outcome:<br>Zung's Self-Rating Anxiety Scale (SAS; anxiety symptoms)<br><br>Predictors:<br>The Chinese version of the Perceived Stress Scale (CPSS-14; stress, not reported how binarised)<br><br>The Cognitive Reappraisal subscale of the Emotion Regulation Questionnaire (EAIM; cognitive reappraisal, not reported how binarised)	Not reported	Moderation analysis using multivariate linear regression models.<br><br>*Anxiety*<br>**Stress (direct effect, positive association)**<br>**Cognitive reappraisal (direct effect, negative association)**<br>**Interaction (cognitive reappraisal moderates the association between stress and *anxiety*)**	Not reported

S5.4 Table. Qualitative outcomes by research aim

Citation	Measures	Method of analysis	Impact of self-isolation on wellbeing	Factors perceived to be associated with wellbeing	Other potentially relevant data
**Domenghino et al., 2022**	Questionnaire sections included medical history, details of SARS-CoV-2 (e.g. reason for testing, symptoms, severity), mental health prior to infection, isolation experience, and current physical and mental health; each section included a 'free text' space for participants to describe additional issues not captured by the survey responses	Comments concerning isolation or mental health were assigned to preliminary categories: circumstances of isolation, positive and negative aspects, mental health burden, and 'various based on the quantitative themes of the analysis'; specific words which came up frequently were also quantified through text search; the authors report 'themes that were repeatedly mentioned or that were considered especially impactful or important from a public health perspective'	Participants reported feelings of depression, loneliness, and aggression due to isolation, as well as feeling sad and that their mental health had worsened. Over '23 comments' (unclear whether this was 23 different people, or multiple comments from the same people) described isolation as being like a prison, torture, solitary confinement, or punishment<br><br>Feared stigma and workplace consequences<br><br>Relationships suffered: e.g. some participants reported conflicts with partners if their views on social contact differed, or feeling more aggressive due to isolation	Those already struggling with mental health pre-isolation felt that it worsened their symptoms<br><br>Worried about others they may have infected, especially their families<br><br>Perceived to be easier to cope with isolation if managers at work were understanding (vs. those who felt their managers 'blamed' them for getting sick or insisting they worked while sick, which added to their stress); managers calling frequently to check how they were doing also helped participants feel more positive<br><br>Not knowing the consequences of infection caused feelings of fear, panic, wondering if they would die<br><br>Isolation described as draining, tiring and discouraging, with participants describing somatic pain due to lack of physical activity and social contact; the more time they spent in isolation, the worse they felt physically<br><br>Participants who worked in health care were particularly concerned about 'abandoning' their colleagues at a time of need, and did not feel protected in the workplace	Phone calls and WhatsApp helped for a while but were no substitute for real contact<br><br>Some used the time to organise their homes, take up a new hobby, learn a language, read books, listen to podcasts, do yoga and home workouts

Citation	Measures	Method of analysis	Impact of self-isolation on wellbeing	Factors perceived to be associated with wellbeing	Other potentially relevant data
				Worries about financial troubles and job security were not frequent, but for those who experienced such worries, they were highly stressed<br><br>Those with children reported that the most stressful parts of isolation were being separated from children and the conflict between home office and childcare<br><br>Participants felt especially 'dissatisfied and alone' if they received contrary isolation instructions from different authorities<br><br>Positive aspects: more time with family, more time to relax, the ability to 'slow down' all helped participants to refocus on important things in life and appreciate what they had	
**Gok, 2022**	Study-specific qualitative survey with 4 questions: how did you feel when you learned you were diagnosed with COVID-19; how did staying at home [in] quarantine affect you when you were diagnosed with COVID-19; when you are diagnosed with COVID-19, can you	Content analysis	115 females and 39 males described psychological effects. Most frequently used codes in the qualitative descriptions of how participants felt about home quarantine: 'negative' (31.3% of 115 females, 23.1% of 39 males); 'loneliness' (13% of females, 15.4% of males); 'boring' (20% of females, 10.2% of males); 'disturbing' (16.5% of females, 15.4% of males). Other codes included 'stressful', 'like a prison', 'despair' and 'sad'	N/A	Aspects of quarantine noted to be particularly difficult:<br><br>35 females and 19 males discussed fears for their family, namely fear of their children being orphaned (51.5% of females, 68.5% of males) and loss of family members (48.5% of females and 31.5% of males)<br><br>110 females and 33 males discussed physiological effects being the 'worst case scenario' they thought about during

Citation	Measures	Method of analysis	Impact of self-isolation on wellbeing	Factors perceived to be associated with wellbeing	Other potentially relevant data
	describe the worst-case scenario you thought of while in quarantine; how did you cope with the uncertainty you were in when you were diagnosed with COVID-19?		14 females and 5 males discussed 'physiological effects', with home quarantine described as 'relaxing' by 64.3% of the females and 0 males, 'exhausting' by 35.7% of females and 60% of the 5 males, and 'difficult' by 0 females and 40% of the males.<br><br>The author also discusses the code 'ineffective' which was discussed by 23 females and 12 males. Home quarantine 'did not affect' 95.7% of these females and 8.3% of these males, and was described as 'mixed' by 4.3% of the females and 91.7% of the males – it is not clear what this means		home quarantine, namely 'death' (31% of females, 21.2% of males), 'shortness of breath' (16.3% of females, 24% of males), 'being admitted to intensive care' (14.5% of females, 12% of males), 'transmitting the virus to others' (15.5% of females, 18% of males), 'serious damage to the body' (17.2% of females, 18% of males) and 'getting sick while in quarantine' (5.4% of females, 6% of males)<br><br>Codes emerging which related to coping with uncertainty included 'positive perspective' (encompassing 'positive thinking', acceptance, and 'trying to be strong' – 57 females and 28 males); 'maintaining a daily routine' (encompassing cleaning/watching movies/reading and healthy eating - 22 females and 9 males); 'social support' (encompassing communication with family/friends, partner support, 'getting strength from the presence of children' and getting information from healthcare professionals – 30 females and 12 males); and 'spirituality' (encompassing prayer, taking refuge in God and

Citation	Measures	Method of analysis	Impact of self-isolation on wellbeing	Factors perceived to be associated with wellbeing	Other potentially relevant data
					being patient – 19 females and 4 males)
**Jesmi et al., 2021**	Semi-structured interviews including questions such as: describe your experience of COVID-19 infection; describe your feelings when informed of your test result; compare your COVID-19 experience with what others and the media describe; describe the experience of a day dealing with the disease; what comes to mind when you hear 'COVID-19 infection'?; what were your expectations from those around you?; 'what comes to mind when you think of a problem?'	Colaizzi phenomenological approach	Mental strains described by participants were divided into 3 categories: concerns, fears and isolation<br>Main fears were of death and disability/dependence on others<br>Most participants reported experiencing loneliness and boredom while quarantined at home	Concern about job loss was reported more frequently by males, self-employed participants and those with no job security<br>Daily media reports of COVID-19 deaths were perceived to contribute to fears<br>Experiencing shortness of breath was perceived to contribute to fear<br>Most participants reported similar concerns: worrying about worsening of symptoms (especially shortness of breath), losing their jobs, future of their children (e.g. what would happen to their children if their parent died), persisting complications, and disease disclosure (fear of telling families and causing them to be worried). Concerns were reported to lead to anxiety, tension, and sleep disorders (insomnia and nightmares)	Coping strategies included: religious activities / beliefs; complementary therapies such as vitamins, soup and herbal tea; relying on family support (including both mental and physical care)
**Lohiniva et al., 2021**	Interviews including questions such as: describe any negative experiences with people during quarantine/isolation or any experiences of stigma; 'tell me about your time in	Thematic analysis; data on stigma were analysed using a health stigma framework which entailed identifying codes and categories within each construct of the framework (drivers, facilitators,	Households with mild COVID-19 symptoms tended to describe quarantine as boring and monotonous<br>Some respondents described quarantine as having created tension at home whilst others saw positive developments, such as getting closer to their partner,	Stigma and self-stigma were perceived to be related to poorer quality of life: see next column<br>Households with severe or prolonged symptoms, or where household members had difficulty accessing testing or being admitted to hospital, tended to describe their quarantine	Stigma:<br>Facilitators of stigma: fear of contracting coronavirus, blame for contracting coronavirus<br>Manifestations of stigma: blame for being irresponsible, gossiping, being overly curious about their COVID-19

Citation	Measures	Method of analysis	Impact of self-isolation on wellbeing	Factors perceived to be associated with wellbeing	Other potentially relevant data
	quarantine'; how did quarantine influence your life?; what was difficult for you during quarantine?	manifestations, outcomes, and impacts of stigma) whereas data on quarantine experiences were analysed based on an inductive coding process	having more family time, or having more time to relax	experience as revolving around symptom management, worries about health and fears of death<br><br>For those isolating due to sick family members, caring for sick household members was considered a heavy responsibility consuming all their time and energy<br><br>Concerns were reported about health, symptoms, death<br><br>Some respondents with children worried their children were being ignored while they managed the illness among other household members<br><br>Knowing someone hospitalised due to COVID-19 and seeing media reports about high infection rates and fatalities abroad were perceived to worsen thoughts/worries about death (many described media coverage as exhausting and stressful but others felt it was helpful and allowed them to prepare themselves for having COVID-19)<br><br>Respondents with active and outgoing lifestyles pre-quarantine were 'particularly bothered' by the restrictive quarantine (vs. those who had little social contact outside of the home and/or worked from home pre-quarantine, who	experience, reluctance to interact<br><br>Stigma outcomes: fear of disclosing COVID-19 status<br><br>Stigma impact: negative influence on social contacts resulting in reduced quality of life<br><br>Self-stigma:<br><br>Facilitators: different and conflicting information about COVID-19, symptoms continuing after quarantine, uncertainty about immunity, uncertainty about having contracted COVID-19<br><br>Manifestations: stressed when outside the home, nervous to meet people, fear of being blamed for leaving the house<br><br>Outcomes: reluctance to meet people, prolonging quarantine/isolation periods<br><br>Impacts: psychological distress leading to reduced quality of life<br><br>Other quarantine experiences:<br><br>Peer support groups (e.g. WhatsApp groups for people who attended an event where they contracted the virus, or group chats with friends who also had COVID-19) were seen by some participants as very

Citation	Measures	Method of analysis	Impact of self-isolation on wellbeing	Factors perceived to be associated with wellbeing	Other potentially relevant data
				did not feel their lives had changed very much as a result of quarantine)<br><br>Worries about the health of family members and guilt at feeling they had infected family members	valuable and empowering and they found it helpful to talk to others going through similar experiences, who understood them; some also said this communication helped reduce their worries about their own health and that of their families; such groups were also seen as a good source of information; however, other participants felt that continuous discussions about the illness in these groups made the illness highly present in their lives and a source of stress
**Verberk et al., 2021**	Semi-structured interviews covering the following topics: experience of home care, transmission prevention practices, impact (of household members' COVID-19 diagnosis) on daily life	Researchers summarised data directly from the audio recordings using an a priori framework that captured key areas of interest based on the research questions	Emotional burden: participants described living with and caring for a relative with COVID-19 as having a significant emotional impact. Some described feelings of helplessness and lack of control whilst others described acceptance and 'taking it day by day'<br><br>Some worried about catching the virus (and potential health impacts of that) and transmitting the virus to others<br><br>Many had experienced an initial reaction of anger towards the infected person; however, participants also expressed feelings of solidarity within the household	Participants expressed concerns about having no clear instructions on how to monitor their infected loved ones; the potential for symptoms to suddenly worsen; and practicalities such as finding childcare if they needed to go to hospital<br><br>Participants perceived that it was more difficult to be the non-infected person in the household because those who had been infected could return to work after symptoms had been resolved for at least 14 days, whereas contacts often found their quarantine duration extended as different household members contracted the virus and some had to quarantine for two months	Stigma: participants described fear of reactions of others who were scared of being infected themselves; they felt judged and seen as contagious and felt people were afraid to be near them<br><br>Participants stressed the importance of staying positive and accepting the situation. Coping strategies included talking to family members and wider support networks, finding time to relax, and making plans for post-quarantine

Citation	Measures	Method of analysis	Impact of self-isolation on wellbeing	Factors perceived to be associated with wellbeing	Other potentially relevant data
			Participants described feelings of boredom, lack of control and feeling 'depressed'	Participants described confusion over inconsistent guidelines and felt that rules were not communicated efficiently. Multiple sources of differing information could create information overload and confusion<br><br>Quarantine was frustrating because they had no clear idea of how long they would need to quarantine for, and some were frustrated at having to enter quarantine just as lockdown measures relaxed	
