# Supplementary material for: The impact of self-isolation on psychological wellbeing in adults and how to reduce it: A systematic review
Source: PLoS One. 2025 Mar 28;20(3):e0310851. doi: 10.1371/journal.pone.0310851 (PMC11952258; doi:10.1371/journal.pone.0310851)
Supplement: S6 Appendix — (PDF) [file pone.0310851.s006.pdf]

Supplementary materials 6

The impact of self-isolation on psychological wellbeing and how to reduce it: a systematic review

Alex F. Martin[1, 2*], Louise E. Smith[1, 2], Samantha K. Brooks[1, 2], Madeline V. Stein[1], Rachel Davies[1], Richard Amlôt[2, 3], Neil Greenberg[1, 2], G James Rubin[1,2]

[1] King's College London, Institute of Psychiatry, Psychology and Neuroscience, London, UK

[2] NIHR Health Protection Research Unit in Emergency Preparedness and Response, London, UK

[3] UK Health Security Agency, Chief Scientific Officer's Group, UK

Contents

S6 APPENDIX: RISK OF BIAS SUMMARIES 2

S6.1 Figure. Quality appraisal for Aim 1 using ROBINS-E for exposure studies, summary by study (left) and domains (right) 2

S6.2 Figure. Quality appraisal for Aim 2 using ROBINS-E for exposure studies 3

S6.3 Figure. Quality appraisal for Aim 2 using ROBINS-I for non-randomised intervention studies 4

S6.4 Table. Quality appraisal – CASP checklist for qualitative studies 5

S6 Appendix: Risk of bias summaries

S6.1 Figure. Quality appraisal for Aim 1 using ROBINS-E for exposure studies, summary by study (left) and domains (right)

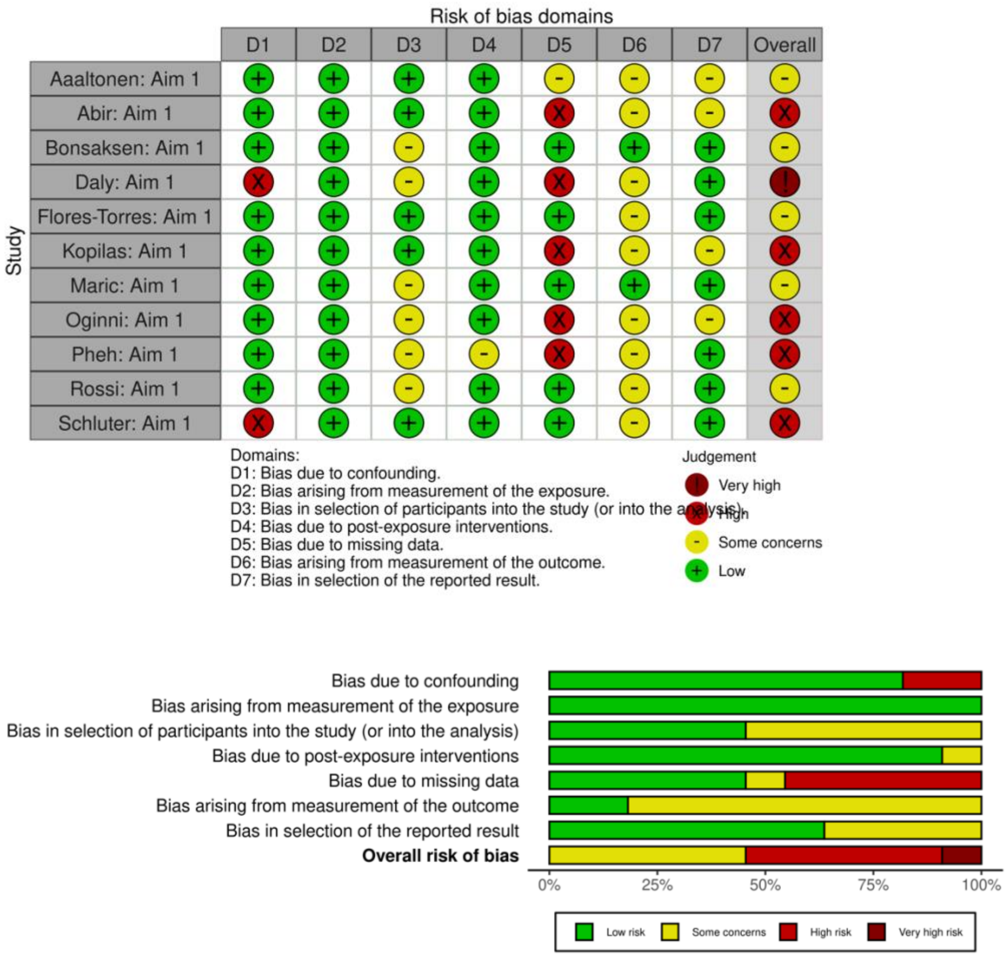

Note. Data visualisation created using McGuinness, L. A., & Higgins, J. P. T. (2020). Risk-of-bias VISualization (robvis): An R package and Shiny web app for visualizing risk-of-bias assessments. *Research Synthesis Methods*. https://doi.org/10.1002/jrsm.1411

S6.2 Figure. Quality appraisal for Aim 2 using ROBINS-E for exposure studies

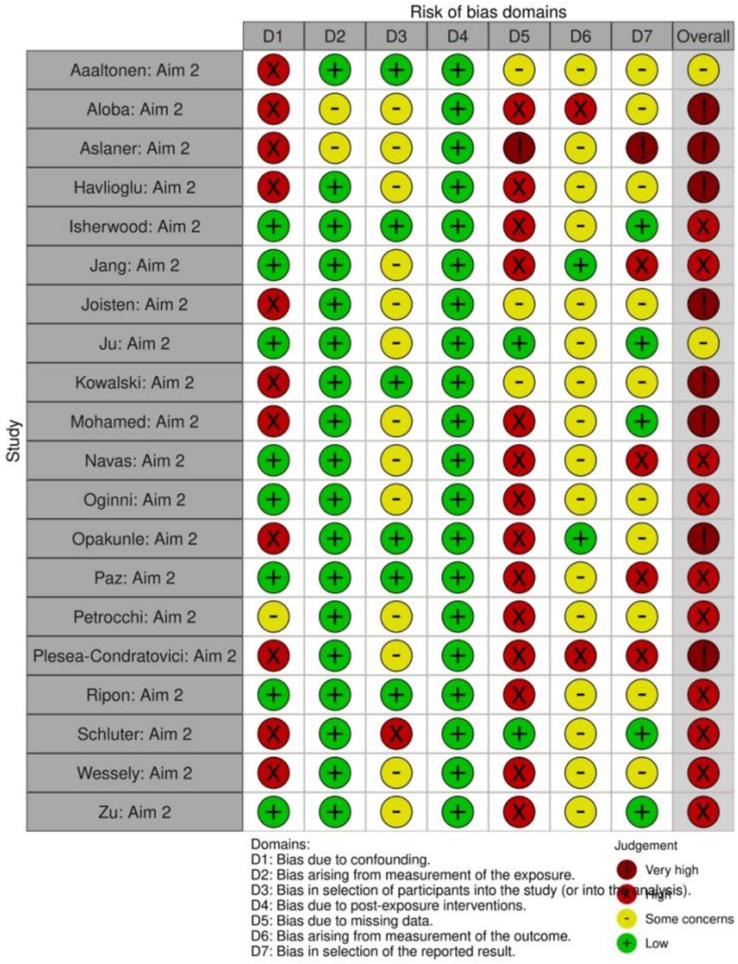

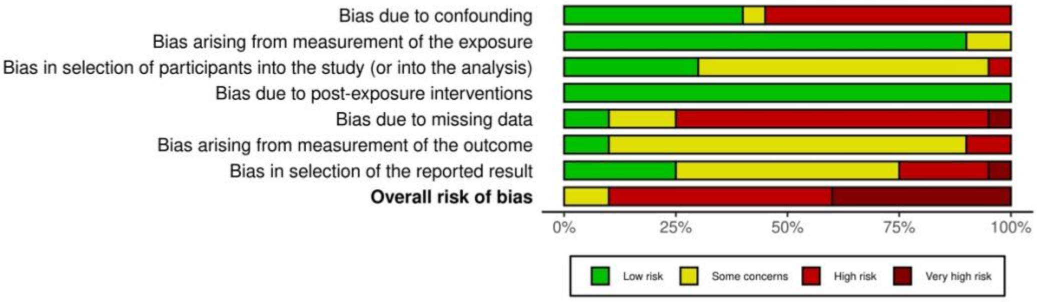

Note. Data visualisation created using McGuinness, L. A., & Higgins, J. P. T. (2020). Risk-of-bias VISualization (robvis): An R package and Shiny web app for visualizing risk-of-bias assessments. *Research Synthesis Methods*. https://doi.org/10.1002/jrsm.1411

S6.3 Figure. Quality appraisal for Aim 2 using ROBINS-I for non-randomised intervention studies

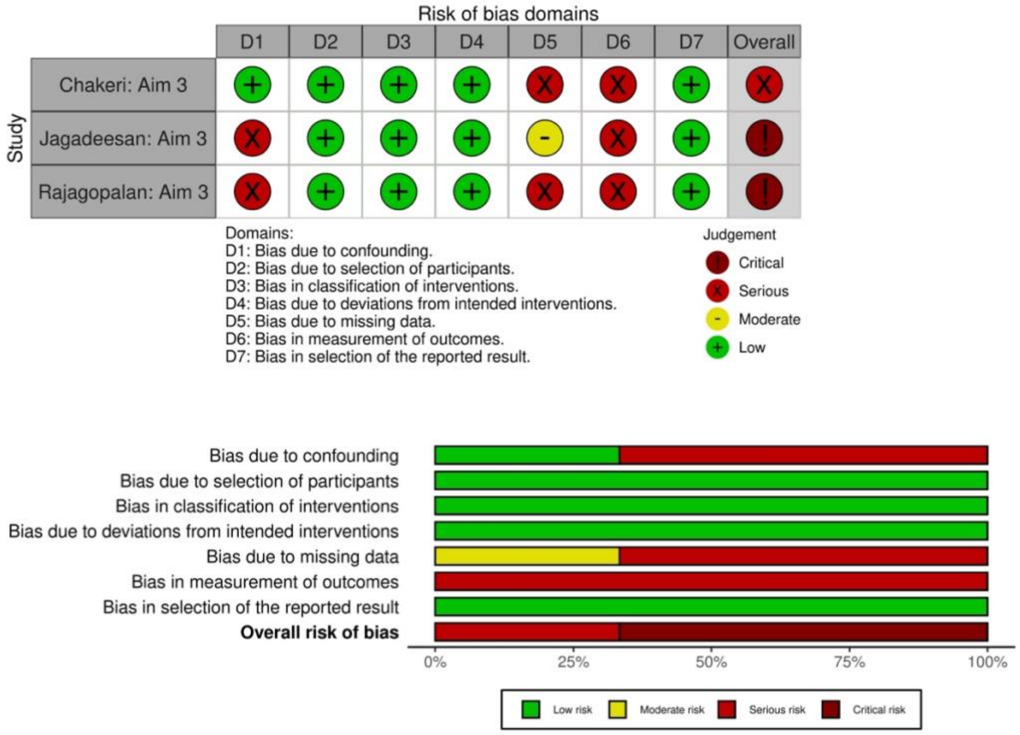

Note. Data visualisation created using McGuinness, L. A., & Higgins, J. P. T. (2020). Risk-of-bias VISualization (robvis): An R package and Shiny web app for visualizing risk-of-bias assessments. *Research Synthesis Methods*. https://doi.org/10.1002/jrsm.1411

S6.4 Table. Quality appraisal – CASP checklist for qualitative studies

Citation	Q1	Q2	Q3	Q4	Q5	Q6	Q7	Q8	Q9	Q10	% of 'yes' answers	Other comments
Domenghino et al., 2022 [8]	Yes	Yes	No	No	No	No	Yes	No	No	Yes	40%	- Analysis not described well and does not appear to be rigorous<br>- Data were categorised into pre-defined categories according to what the authors expected to find / found important<br>- Not reported how many qualitative comments there were or from how many people<br>- Participants who did not meet the study's inclusion criteria were included (e.g. lowest age was 17, but 17-year-olds should have been excluded)
Gok, 2022 [10]	Yes	Can't tell	No	No	No	No	Yes	No	No	No	20%	- Population size is reported differently in different places in the paper (202 vs 212)<br>- Date of data collection is reported differently in different places in the paper<br>- Wording of Q4 on the survey is leading<br>- Codes are not well-explained<br>- Quotes presented do not always relate well to the 'codes' they have been given<br>- Unclear why the %s always add up to 100% in the tables – could participants not give more than one answer? This is not explained<br>- Author describes rates being 'higher in females' but they do not perform any statistical analysis to know whether this is a significant finding<br>- Very little discussion of implications of the study
Jesmi et al., 2021 [15]	Can't tell	Can't tell	Can't tell	Can't tell	Yes	No	No	No	Yes	No	20%	- Aim is very vague ('lived experiences' of those with COVID-19)

											- Ethical approval was granted but only 8/14 participants completed informed consent forms
Lohiniva et al., 2021 [20]	Yes	Yes	Yes	No	No	No	Yes	No	Yes	Yes	60%	
Verberk et al., 2021 [34]	Yes	Yes	Yes	Can't tell	Yes	No	Yes	No	Yes	Yes	70%	- Explanation of how analysis was done is insufficient; potential for bias due to the a priori framework based on 'areas of interest'

Key:

- **Q1: Was there a clear statement of the aims of the research?**
- **Q2: Is a qualitative methodology appropriate?**
- **Q3: Was the research design appropriate to address the aims of the research?**
- **Q4: Was the recruitment strategy appropriate to the aims of the research?**
- **Q5: Was the data collected in a way that addressed the research issue?**
- **Q6: Has the relationship between researcher and participants been adequately considered?**
- **Q7: Have ethical issues been taken into consideration?**
- **Q8: Was the data analysis sufficiently rigorous?**
- **Q9: Is there a clear statement of findings?**
- **Q10: Do the authors discuss the value of the research in terms of implications and contributions to literature? (please note Q10 has been reworded from 'How valuable is the research?' to enable yes/no answers**
