# Supplementary material for: The impact of self-isolation on psychological wellbeing in adults and how to reduce it: A systematic review
Source: PLoS One. 2025 Mar 28;20(3):e0310851. doi: 10.1371/journal.pone.0310851 (PMC11952258; doi:10.1371/journal.pone.0310851)
Supplement: Attachment — Submitted filename: Response to Reviewers.docx [file pone.0310851.s007.docx]

**The impact of self-isolation on psychological wellbeing in adults and how to reduce it: a systematic review**

**Response to reviewers**

We are grateful to the reviewers for their feedback and insightful comments that have considerably improved the manuscript, particularly around clarity and consistency, accessibility, and readability. Please find our responses below.

**Reviewer 1**

1. One weakness was inconsistencies between the stated conclusions and the paper’s findings. For instance, the conclusion states that self-isolation impacts psychological wellbeing, but the quantitative data appear far more mixed on isolation’s actual effects outside of its impacts on PTSD.

Thank you for this important point, we completely agree. We have revised the text accordingly in the conclusion on p22:

‘Self-isolation can impact psychological wellbeing, especially PTSD symptoms, but quantitative data shows mixed results for other wellbeing outcomes. Self-isolating at home may reduce the risk of PTSD symptoms, but more and better-quality evidence is needed across all wellbeing outcomes. When implementing self-isolation directives in the future, public health officials should make it a priority to support vulnerable individuals with pre-existing physical and mental health conditions, a lack of support, or those who face additional life stressors. Clinicians and healthcare workers can play a key role in identifying and supporting those most at risk. Interventions should focus on addressing loneliness, worries, and misinformation, improving coping strategies, and monitoring and identifying individuals who need additional support.’

Some more minor issues to address:

2. The authors may want to justify only searching UK agencies and organisations, as this may introduce bias toward sources in the UK context

Please find the method updated accordingly on pp.4-5:

‘A Google search and searches of the websites for relevant UK agencies and organisations (e.g. the UK Testing Initiatives Evaluation Board, the UK Office for National Statistics), and direct inquiries with UK Government agencies were used to identify other potentially relevant sources. We searched only UK agencies due to our team’s experience. Combined with the UK-based Google search, this may have resulted in missing some grey literature from outside the UK (although we note that these searches did not find any studies for inclusion).’

3. The authors should clarify earlier in the study that the review only considered adults isolating at home, since this limits the review’s applicability

We agree and have updated the manuscript in several places to reflect this:

The title on p.1: ‘The impact of self-isolation on psychological wellbeing in adults and how to reduce it: a systematic review’

The abstract on p.2: ‘**Objective:** To synthesise evidence on the impact of self-isolation at home on the psychological wellbeing of adults in the general population during the COVID-19 pandemic.’

The introduction on p.3: ‘Most studies during the COVID-19 pandemic focused on the impact of "lockdown" measures (broad population stay-at-home orders) on psychological and emotional* wellbeing (hereafter *wellbeing*) [2]. However, the impact of home-based self-isolation on adult wellbeing globally has not yet been systematically reviewed.**

This is important given that a) studies carried out in other infectious disease contexts indicate that self-isolation may be associated with psychopathology symptoms and broader wellbeing outcomes in adults, such as insomnia and substance use.’

**Emotional wellbeing was added in response to reviewer 2, point 4.*

*** The last sentence has been amended in response to reviewer 2, point 6.*

4. The authors note that the groupings were not defined a priori (line 159-162). The authors may want to specify how these groupings were chosen

Thank you, we have updated the manuscript to specify how groups were chosen on p.8:

‘For aim 1, we compared outcomes for those in self-isolation with those not self-isolating. For aim 2, we also grouped studies by factor (isolation, demographic, COVID-19, and mental/physical health characteristics) and associations between factors and self-isolation were reported. These groupings were not defined a priori, but the themes emerged during the process of extracting the factors and were revised iteratively as we synthesised the findings.’

5. The description of study characteristics would flow better if the authors resynthesized the information in a table, which would be more helpful in assessing bias

We have now summarised the study characteristics in Table 1 (pp10-11) and moved the full version of the table to the appendix (S4.1 Table).

6. The methods section notes that the review included adults who isolated at home and excluded children, healthcare workers, and those in managed facilities. However, some of the included studies do not explicitly have age as an inclusion criteria listed in table 1 (for example, Chakeri et al., 2020 and Plesea-Condratovici et al., 2022). The authors should either update the table to reflect whether the data only included adults or otherwise justify those studies’ inclusion

This is a very good point, thank you. We have updated Table 1 – now appendix (S4.1 Table) - to show why studies were included if age was not specified in the study inclusion criteria (5 studies).

7. Table 2, specifically panel B, is somewhat difficult to follow. On first reading, it was not evident whether the arrows indicated difference in effect sizes comparing the groups mentioned to some control or the observed effects among that specific group. In addition, the colours in table 2 differed from that presented in the note. The authors should specify what the arrows entail by either updating the panel’s title or altering the note.

We have updated the note for Table 2 (p.13) so that it shows more clearly what the arrow direction indicates in each panel. As suggested by reviewer 2 (point 1), we have also updated the colour scheme so that it is accessible for those with colour-blindness:

*‘Note.* Panel A = aim 1 the impact of self-isolation on wellbeing, up/down arrow indicates a significant increase/decrease of symptoms in the isolating group, horizontal arrow indicates no effect identified; Panel B = aim 2 factors associated with wellbeing, up/down arrow indicates a significant increase/decrease of symptoms in the group in parenthesis, horizontal arrow indicates no effect identified; Panel C = aim 3 wellbeing interventions during isolation, down arrow indicates a significant decrease of symptoms following the intervention; effect arrows are ordered by risk of bias (risk of bias = ROBINS-E/I [Exposure/Intervention]) dark blue colour = some concerns, red colour = high risk, black colour = very high risk’

Reference: [The best charts for color blind viewers | Blog | Datylon](https://www.datylon.com/blog/data-visualization-for-colorblind-readers)

8. Despite a consistent association between viral load and an increase in psych symptoms, the authors do not address this further.

This is an interesting point and viral load is already included in the results. We have now added the following to the discussion on p.20:

Self-isolation was found to associate with wellbeing differently, depending on individual factors and context. There was good evidence that people with greater mental and physical health needs [24, 27, 30, 35, 42, 45, 52], who experienced COVID-related stressors including inadequate support [24, 30, 31, 35, 36, 42, 50], and had reduced coping strategies [35, 51, 52], were most at risk of adverse outcomes. Of interest, the two studies that examined viral load reported an increase in general psychological symptoms (but not other symptoms) during self-isolation [35, 41]. Previous research found that viral load is associated with disease severity and duration, particularly in older adults, suggesting it may present a useful biomarker of risk during infection mitigation strategies, warranting further investigation [57].

9. The decision to focus on adults as opposed to young people is a large deviation from the registered protocol that may warrant mentioning in the body of the paper.

We have now included this in the method on p.4:

The protocol was prospectively registered on PROSPERO ([CRD42022378140](https://www.crd.york.ac.uk/PROSPERO/display_record.php?RecordID=378140)). The biggest change from the protocol was to only include studies of adults in this review, due to the number of studies identified in the initial search, deviations from the protocol are reported in full in S2 Appendix.

**Reviewer 2**

1. Colour Blindness Accessibility in Table 2 - I suggest using an intuitive yet accessible color scheme for Table 2 to accommodate readers with color blindness. This ensures that all readers can interpret the data effectively.

Thank you for this important point. After considering several recommendations for accessibility, we have changed the arrows in Table 2 (p.13) to contrasting colours, using dark blue, red, and black. Reference: [The best charts for color blind viewers | Blog | Datylon](https://www.datylon.com/blog/data-visualization-for-colorblind-readers)

2. Demographic Characteristics - Including a short discussion on the demographic characteristics of all study participants would help identify who these results may be applicable to or not. This consideration is important for understanding the generalizability of the findings.

We have discussed the point, and it is not clear how we can present the demographic characteristics of study participants, because a) we are not pooling all studies as in a meta-analysis, and b) different countries will have different representative socio-demographics. We have updated the text to make this limitation clearer:

Results (p.9): ‘Studies investigated the general population, rather than sub-groups within the population.’

Limitations (p.22): ‘Limitations of the review were first, seeing as we synthesised results from studies conducted around the globe, the sample included in individual studies may be representative of the general population of that country, but may not be representative of other countries. Second, we were unable to formally analyse publication bias…’

3. Exclusion of Social Isolation - The authors do not explain why social isolation is excluded from the scope of the study. Since social isolation and self-isolation could overlap, both involving reduced social interactions, providing a rationale for this exclusion would enhance the manuscript's clarity.

Social isolation was excluded from the search parameter related to the identification of self-isolation, because it affected the integrity of this element of the search (i.e. to capture studies on self-isolation as per our study definition). We have updated the text to make this point clearer in the method, p.5:

‘The search strategy included terms for COVID-19 AND isolation and quarantine (combined with NOT social isolation) AND psychological wellbeing. Social isolation was excluded as a self-isolation search term because it generated a large number of citations that did not meet our definition of isolation or quarantine.’

4. Clarification of "Wellbeing" - "wellbeing" is broad. Clarifying which aspects of wellbeing are being examined would be beneficial. For example, specifying "The impact of self-isolation during the COVID-19 pandemic on psychological and emotional wellbeing" would provide clearer context.

Thank you, we have added this clarification where we provide:

- the abstract (p.2) **Objective:** To synthesise evidence on the impact of self-isolation at home on the psychological and emotional wellbeing of adults in the general population during the COVID-19 pandemic.
- the definition of self-isolation in the introduction (p.3) ‘Most studies during the COVID-19 pandemic focused on the impact of "lockdown" measures (broad population stay-at-home orders) on psychological and emotional wellbeing (hereafter *wellbeing*).’
- the discussion (p.19) ‘This systematic review summarises the literature on self-isolation and psychological and emotional wellbeing during the COVID-19 pandemic.’

5. Definition of Self-Isolation - The definition of self-isolation could be clearer. The current definition mixes the concepts of isolation and quarantine. Consider rephrasing for better clarity: "Self-isolation in this study is defined as both isolation (separating those who are sick from those who are well) and quarantine (separating those at risk of illness from those who are well)."

We have updated the text accordingly on p.3:

‘Self-isolation in this study is defined as both isolation (separating those who are sick from those who are well) and quarantine (separating those at risk of illness from those who are well).’

6. The rationale in the introduction "How self-isolation specifically impacted wellbeing is less clear” could be reworded to improve clarity and enhance the overall comprehension of the manuscript.

Thank you for highlighting this important point of clarification. The text on p3. Has been updated as follows:

‘However, the impact of home-based self-isolation on adult wellbeing globally has not yet been systematically reviewed.’
